# miRNA expression profiling and zeatin dynamic changes in a new model system of *in vivo* indirect regeneration of tomato

**DOI:** 10.1371/journal.pone.0237690

**Published:** 2020-12-17

**Authors:** Huiying Cao, Xinyue Zhang, Yanye Ruan, Lijun Zhang, Zhenhai Cui, Xuxiao Li, Bing Jia

**Affiliations:** College of Biological Science and Technology, Liaoning Province Research Center of Plant Genetic Engineering Technology, Shenyang Key Laboratory of Maize Genomic Selection Breeding, Shenyang Agricultural University, Shenyang, China; University of Tsukuba, JAPAN

## Abstract

Callus formation and adventitious shoot differentiation could be observed on the cut surface of completely decapitated tomato plants. We propose that this process can be used as a model system to investigate the mechanisms that regulate indirect regeneration of higher plants without the addition of exogenous hormones. This study analyzed the patterns of trans-zeatin and miRNA expression during *in vivo* regeneration of tomato. Analysis of trans-zeatin revealed that the hormone cytokinin played an important role in *in vivo* regeneration of tomato. Among 183 miRNAs and 1168 predicted target genes sequences identified, 93 miRNAs and 505 potential targets were selected based on differential expression levels for further characterization. Expression patterns of six miRNAs, including sly-miR166, sly-miR167, sly-miR396, sly-miR397, novel 156, and novel 128, were further validated by qRT-PCR. We speculate that sly-miR156, sly-miR160, sly-miR166, and sly-miR397 play major roles in callus formation of tomato during *in vivo* regeneration by regulating cytokinin, IAA, and laccase levels. Overall, our microRNA sequence and target analyses of callus formation during *in vivo* regeneration of tomato provide novel insights into the regulation of regeneration in higher plants.

## Introduction

Tissue culture established over 150 years ago continues to play an important role in plant propagation, and continues to be utilized in both basic and applied plant research, including gene transformation and molecular breeding [1,2]. In-depth studies into mechanisms of regulation of regeneration of higher plants using *in vitro* culture techniques, identified several proteins and transcription factors such as WUSCHEL (WUS), SHOOT MERISTEMLESS (STM), BABY BOOM (BBM), and MONOPTEROS (MP) [3–6]. However, both direct regeneration and indirect regeneration via an intermediate callus phase are introduced by various plant growth regulators supplemented media in traditional tissue culture. Yin [7] reported that 157 unique proteins were significantly differentially expressed during callus differentiation in rice when treated with different relative concentrations of the hormones cytokinin and auxin. Additionally, even though somatic embryogenesis (SEG) has been proposed to be a model system of plant embryogenesis, the expression of gene families such as those of miR397 and miR408 was detected in somatic embryos (SE), but greatly decreased in zygotic embryos (ZE) in conifer species [8].

An interesting phenomenon has been observed that *in vivo* adventitious shoots can be regenerated from cut surfaces of stems or hypocotyls after removal of both apical and axillary meristems in some species such as Cucurbita pepo [9], tomato [10], and poinsettia [11]. In tomato, the surface of cut stems regenerates plenty of shoots via callus formation. This *in vivo* generation does not depend on the presence of exogenous hormones. We propose that this phenomenon is particularly useful as a model system to study the innate molecular mechanisms of plant regeneration.

MicroRNAs (miRNAs) are a class of small noncoding-RNAs (20–24 nt) that regulate gene expression at post-transcriptional levels by directly binding to their targets [12,13]. In the past 20 years, miRNAs have been shown to play key roles at each major stage of plant development [14–17]. Furthermore, recent studies have shown that miRNAs are involved in callus initiation, formation and differentiation. For example, the expression levels of miR408, miR164, miR397, miR156, miR398, miR168, and miR528 were up-regulated during maize SE induction [18]. Another study demonstrated that over-expression of miR167 inhibited somatic embryo formation by inhibiting the auxin signaling pathway in *Arabidopsis* [19]. In citrus, the ability of the callus to form SEs was significantly enhanced by either over-expression of csi-miR156a or by individual knock-down of its two target genes, *CsSPL3* and *CsSPL14* [20].

Recently, the large scale application of next-generation sequencing has proved to be a useful tool to identify the patterns of miRNA expression during plant regeneration. Genome-wide miRNAs and their targets have been analyzed during explant regeneration in vitro in wheat [21], rice [22,23], cotton [24], peanut [25], sweet orange [26], coconut [27], larch [28], maritime pine [8], Norway Spruce [29], longan [30], yellow-poplar [31], radish [32], Lilium [33], and Tuxpeno maize [34]. However, all these studies were performed on *in vitro* specimens, which relys on the presence of exogenous hormones to regenerate plantlets. The aim of the present study was to identify the pattern of miRNA expression during callus formation in *in vivo* regeneration in tomato through sequencing. We assessed 92 known miRNAs and identified 91 novel miRNAs, of which several were found to be developmentally regulated. We also analyzed dynamic changes in cytokinin levels during *in vivo* regeneration of tomato.

## Materials and methods

### Plant materials

The tomato cultivar micro-TOM was used in this study. Seeds were placed on moistened filter papers for approximately 3 to 4 d until the seeds sprouted. The germinated seeds were seeded into a tray of 72 cells filled with a mixture of nutrient soil, matrix, vermiculite and perlite (2:2:1.5:0.5(v/v/v/v)), and grown in a culture room with temperature ranging from 23 to 28°C and 16/8 h light/dark photoperiod. When the seedlings had grown 6 to 8 true leaves, the primary shoot was decapitated horizontally. All axillary buds that appeared after decapitation were resected at the base (dx.doi.org/10.17504/protocols.io.bn5xmg7n).

### HPLC analysis of trans-zeatin

Cutting surfaces of stems (3 mm long) were sampled at 0, 9, 12, 15, 18, 21, 24 and 30 d after decapitation inthree biological replicates. Each biological replicate was from a pool of 3–5 tomato plants, and weighed to obtain the fresh weight. The samples were immediately frozen in liquid nitrogen and stored at –80°C. Trans-zeatin was extracted with 80% methanol from samples and standard substance, and analyzed using HPLC (Agilent 1100, Agilent Technologies, CA, USA) connected to an UV detector (λ = 274 nm). The HPLC of each sample was repeated three times.The passing fraction was further purified by Sep-pak C18 column (Waters, Milford, MA, USA). Gradient elution was with a mixture of water-methanol (75:25 (v/v)) with an elution rate of 1.0 mL/ min at a column temperature of 35°C. The absorbing material was Agilent C18 with a particle size of 5 μm loaded into a stainless steel column (250 × 4.6 mm).

### Lovastatin addition during the tomato regeneration *in vivo*

A lovastatin (Sigma-Aldrich, St. Louis, MO, USA) stock solution at a concentration of 0.124 M was prepared by dissolving in DMSO and applied at a final concentration of 123.6 μM. A solution of 20 μL lovastatin and 1 μL Tween-20 was applied on the cut surfaces of 10 tomato stems after decapitation. An additional equal number of tomato plants were treated with water as control. The number of regenerated adventitious buds was counted on 0, 30, 37, 44, 51 and 58 d after decapitation, respectively. We used ANOVA to test the difference between the treatment with and without lovastatin added.

### sRNA library construction and RNA sequencing

Total RNA was extracted from decapitated stem at 0 and 15 d in three biological replicates. Each biological replicate was from a pool of 8–10 tomato plants. RNA samples of the three biological replicates were mixed in equal amount and used for the construction of libraries. The small RNA library was constructed using 3 μg total RNA from each treatment respectively as input materials. The sequencing library was generated by NEBNext^®^ Multiplex Small RNA Library Prep Set for Illumina^®^ (NEB, Ipswich, MA, USA), with added index codes to attribute sequences of each sample as recommended by the manufacturer. Briefly, the NEB 3ʹ SR Adaptor was connected to 3ʹ end of miRNA, siRNA and piRNA directly. After the 3ʹ ligation reaction, the single-stranded DNA adaptor was transformed into a double-stranded DNA molecule by hybridization of the SR RT Primer with excess of 3ʹ SR Adaptor (kept free after the 3ʹ ligation reaction). This step significantly reduced the formation of adaptor-dimers. In addition; dsDNAs were not the T4 RNA Ligase 1-mediated-substrates, and therefore were not ligated to the 5ʹ SR Adaptor in the following ligation step. The 5ʹ ends adapter was connected to the 5ʹ ends of miRNAs, siRNA, and piRNA. Reverse transcription reaction was performed using M-MuLV Reverse Transcriptase (RNase H^–^) after ligation with adapters, and Long Amp Taq 2X Master Mix, SR Primer for Illumina and index (X) primer was used for PCR amplification. An 8% polyacrylamide gel was used for purifying PCR products, small RNA fragments approximately 140–160 bp were recovered and dissolved in elution buffer. Finally, the quality of library was evaluated on the 2100 system of Agilent Bioanalyzer using DNA High Sensitivity Chips. TruSeq SR Cluster Kit v3-cBot-HS (Illumina, San Diego, CA, USA) was used to evaluate index-coded samples on a cBot Cluster Generation System. After clustering, the library preparations were sequenced on an Illumina Hiseq 2500 platform, and 50 bp single-end reads were generated.

### miRNA identification and target prediction

All sequenced data were firstly filtered with the removal of N% >10% reads, length <18 nt or >30 nt, with 5ʹ adapter contamination, 3ʹ adapter null or insert null and low quality reads to obtain clean reads. The remaining clean reads were mapped to the reference sequence by Bowtie to obtain unique reads and analyze the length distribution and expression of unique sRNAs. Unique reads were mapped to miRNA, rRNA, tRNA, snRNA, snoRNA, repeat masker, NAT, TAS, exon, intron, and others. Mapped sRNA tags were used to search for known miRNAs. With miRBase20.0 (http://www.mirbase.org) as reference, the potential miRNAs were identified using modified software mirdeep2 [35] and srna-tools-cli (http://srna-tools.cmp.uea.ac.uk/), and then the secondary structures were drawn. We used miREvo [36] and mirdeep2 to predict novel miRNAs through the precursor structure of each miRNA unannotated in the previous steps, including the analysis of the secondary structure, the dicer cleavage site, and the minimum free energy. We used miFam.dat (http://www.mirbase.org/ftp.shtml) to compare our candidate miRNA families with known miRNA families from other species.

We used psRobot_tar in psRobot [37] to identify the potential gene targets of known and novel miRNAs.

### Differential expression analysis of miRNAs

We used the TPM (transcript per million) value to estimate the differential expression levels of miRNAs between stem and callus [38]. The TPM ratio of miRNAs between stem and callus libraries was computed as log_2_ (callus/stem). miRNAs with p value <0.05 and log_2_ (callus/stem) <−1 or >1 were regarded to have as significantly differential expression levels through the Bayesian method.

### miRNAs quantification by qRT-PCR

Primers were designed by stem-loop method to perform RT-PCR assays according to Chen’s design [39]. A 20 μL final volume Reverse transcription (RT) reaction was carried out to validate the expression levels of selected miRNAs extracted from stem and callus respectively. The final 20 μL reaction system included 1 μL template, 1 μL stem-loop primer, 1 μL dNTP mixture, 4 μL buffer, 1 μL reverse transcriptase and 0.5 μL RNase inhibitor. In the reaction tube, stem-loop primer, dNTP mix and template were added first, and then the template was denatured at 65°C for 5 min to improve the efficiency of reverse transcription. The tube was placed on ice to cool for 2 min, followed by the addition of the buffer, reverse transcriptase and RNase inhibitor. The tube was then incubated at 45°C for 60 min, followed by 95°C for 5 min. The reverse transcription reaction was completed by cooling on ice for 2 min. After reverse transcription, 1 μL of the RT reaction mixture was used for PCR. The PCR system was 25 μL, containing 12.5 μL PCR mix, 1 μL template, 1 μL downstream primer and 1 μL upstream primer, supplemented to 25 μL with nuclease-free water. The PCR conditions were as follows: 94°C for 2 min, 94°C for 30 s, 60°C for 1 min for 35 cycles, followed by a final extension of 72°C for 5 min. Following the PCR assay, gel electrophoresis was used to detect the amplified products.

Quantitative reverse transcription-polymerase chain reaction (qRT-PCR) assays were performed using a C1000 Touch^TM^ Thermal Cycler (Bio-Rad, Hercules, CA, USA). The reaction system included 5 μL SYBR^®^ Premix Ex Taq^TM^ (Takara, China), 1 μL template, 0.2 μL upstream primer, 0.2 μL downstream primer and 3.6 μL nuclease-free water. The reaction conditions were as follows: 94°C for 2 min, 94°C for 30 s, 60°C for 1 min, and final extension at 72°C for 5 min for 35 cycles. The sequences of all primers used in this study are compiled in S1 Table.

## Results

### Phenotypic analysis of *in vivo* regeneration

**Fig 1:**
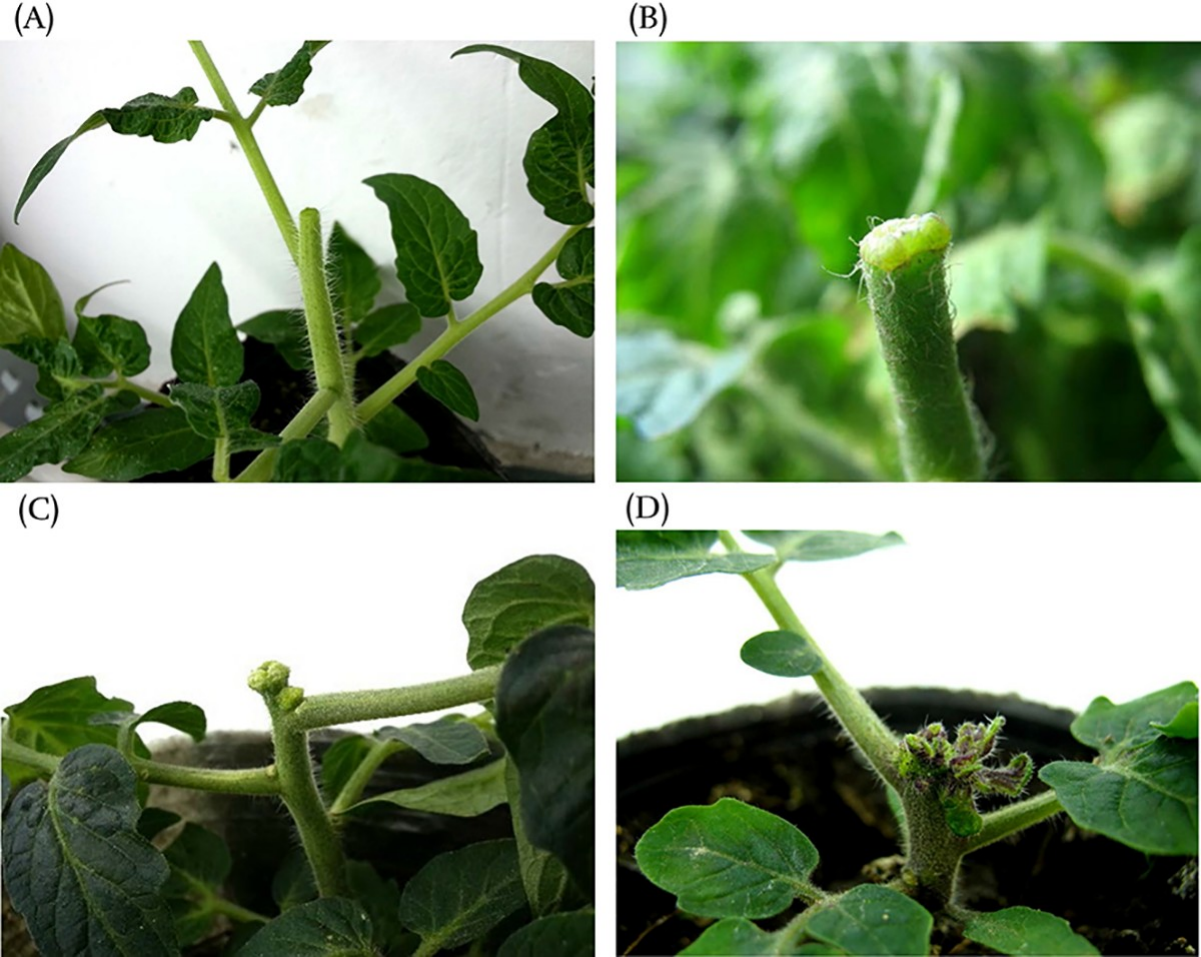
External appearance of the different stages of *in vivo* regeneration in tomato. (A) The decapitated primary shoot; (B),(C) The callus formed on the cutting surface at 15 and 25d after decapitation; (D) The adventitious shoots differentiated from callus.

In tomato, a primary shoot shows apical dominance and inhibits outgrowth of axillary buds. After excising the main shoot apex, the dormant axillary buds began to develop immediately to replace the lost shoot apex. Since all new axillary buds were excised, light-green callus gradually formed at the cut surface of primary shoots and axillary buds followed by progression to the compact and nodular stage with a maximum diameter of up to 1 cm. When the callus entered its differentiation stage, a large number of purple dots appeared on its surface, and finally the shoots appeared to regenerate through callus (Fig 1). It took 30 days to obtain macroscopic shoots after decapitation at 25°C. These features of *in vivo* regeneration were similar to the responses seen in tissue culture [58].

### Analysis of trans-zeatin during *in vivo* regeneration

**Fig 2:**
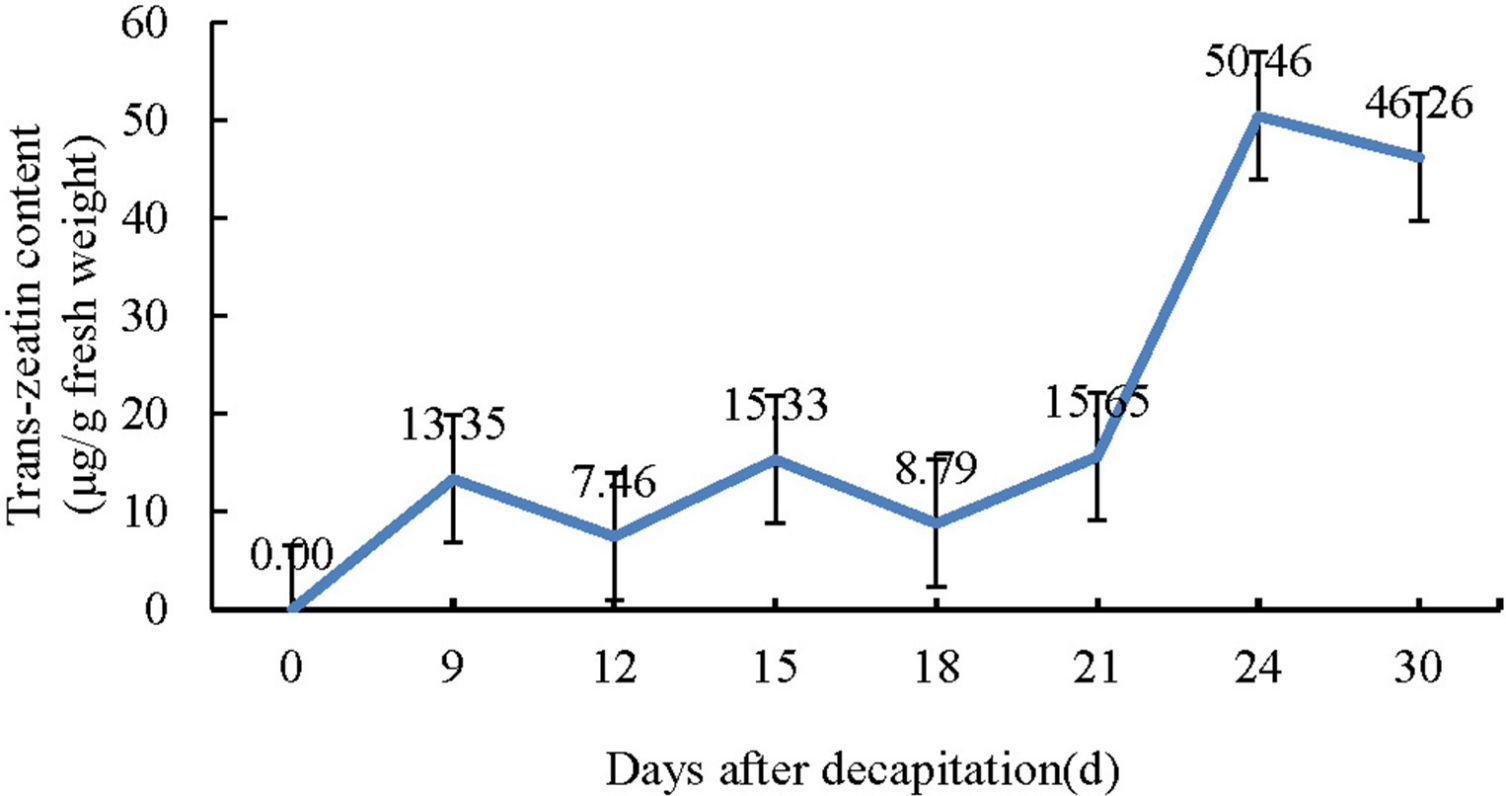
HPLC analysis of trans-zeatin levels during *in vivo* regeneration of tomato micro-TOM.

We used HPLC to determine the standard substance firstly, the standard curve of peak area and trans-zeatin weight was y = 0.0651x–3.3111, R^2^ = 0.9963. Then the peak areas of the samples were detected by HPLC, and the weight of trans-zeatin were calculated and taking the average value for three biological replicates. Finally, the contents of trans-zeatin in the samples were obtained by dividing the weight by the fresh weights of the samples. HPLC analysis of trans-zeatin during *in vivo* regeneration of tomato micro-TOM is presented in Fig 2 and S2 Table. Trans-zeatin was not detected in 0 d stem, but was detected in gradually increasing amounts correlated with the progress of callus initiation, formation and differentiation. These results show that cytokinin plays a key role during *in vivo* regeneration of tomato.

The samples were the cutting surface of stem at 0, 9, 12, 15, 18, 21, 24 and 30 d after decapitation, 3–5 tomato plants were collected in each sample.

**Fig 3:**
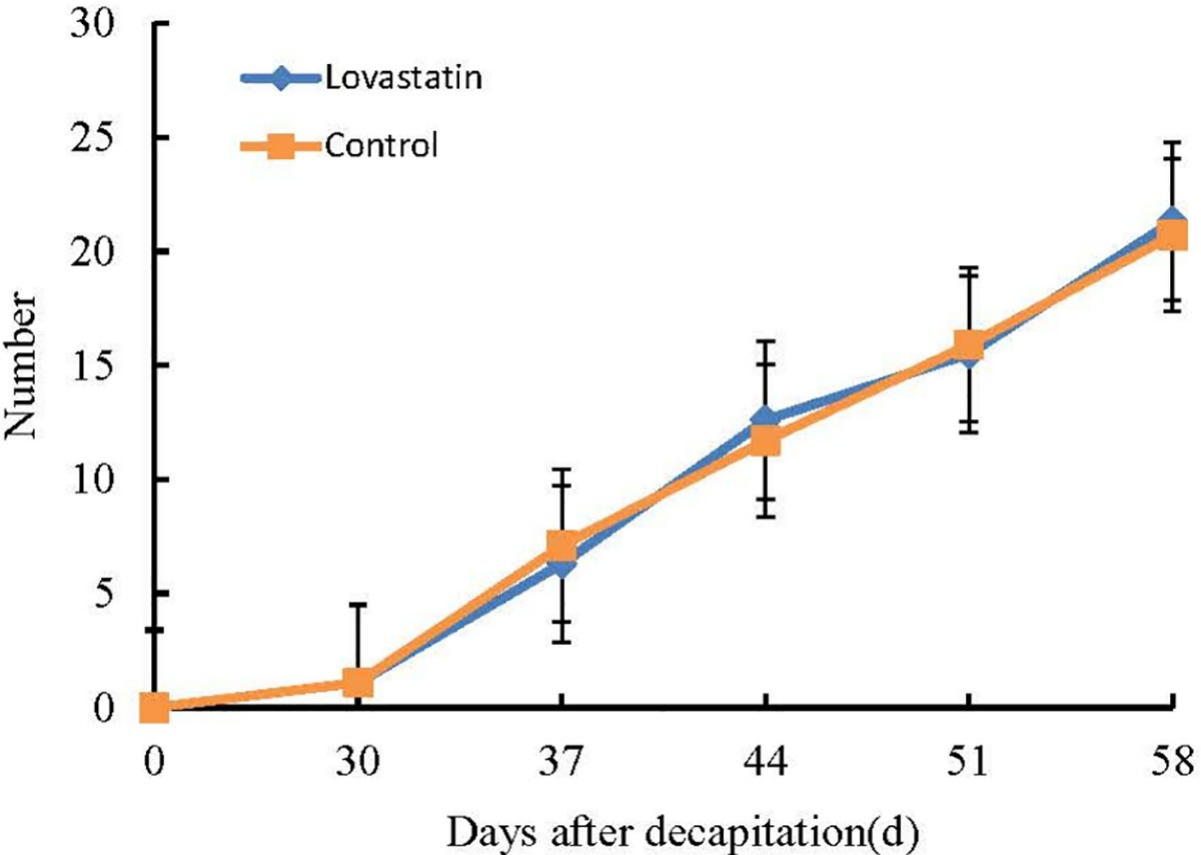
Regenerated adventitious shoots levels in lovastatin and control treated tomato plants.

Cytokinins are a heterogenous group of N6-substituted adenine derivatives [40]. Lovastatin is a potent inhibitor of the mevalonate pathway, and in principle blocks the synthesis of isopentenyl-pyrophosphate and inhibit the biosynthesis of cytokinin [41]. Lovastatin (1 μM) has been shown to completely inhibit the growth of cultured tobacco cells [42]. However, in this study, the addition of high levels lovastatin (123 μM) to the cut surface of decapitated stems did not inhibit tomato regeneration *in vivo*. There was no obvious difference (the P-value was 0.8524) in the number of regenerated adventitious shoots between lovastatin and control treated plants by variance analysis (Fig 3 and S3 Table). Together, these observations suggested that cytokinin was not biosynthesized de novo in the cells at the cut surface or in the callus during *in vivo* regeneration.

### Deep-sequencing of sRNAs in stem and callus

**Fig 4:**
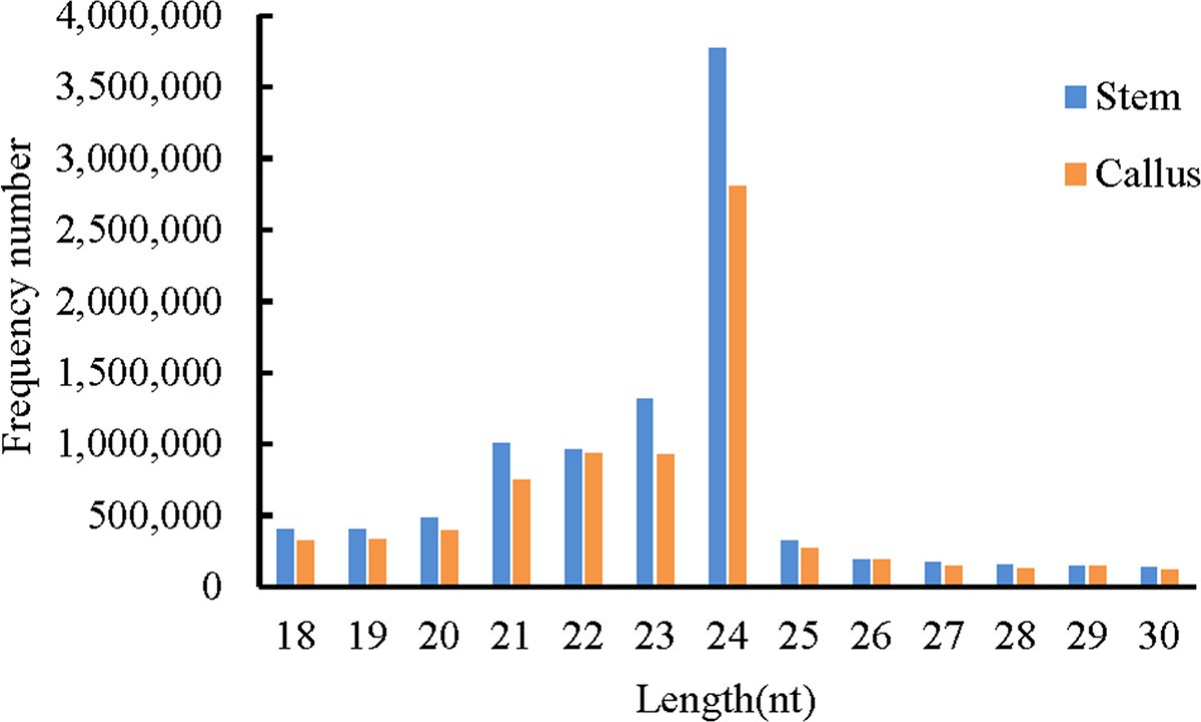
Length distributions of unique sRNAs in stem and callus.

To study the vital role of miRNAs during *in vivo* regeneration, the cut surfaces of stems at 0 and 15 days after decapitation were used to construct two sRNA libraries. Both of these libraries were sequenced with 13.6 and 11.0 million raw reads obtained from stem and callus libraries, respectively (S4 Table). After removal of the low quality reads as described in the Materials and Methods section, 9.5 and 7.5 million clean sRNAs were obtained from the stem and callus libraries. Among these, 7.4 and 6.6 million were unique reads aligned to the reference sequences (Table 1). Unique sRNAs ranged from 18 to 30 nt in length in the two libraries (Fig 4). The most common lengths of unique sequences in each library were 21–24 nt, with 24 nt long reads being the majority, followed by 23 nt.

**Table 1:** Sequencing data filtering of two sRNA libraries produced from stem and callus.

Type	Number of reads (Percentage of reads)	
	Stem	Callus
Raw reads	13,563,211 (100.00%)	10,988,386 (100.00%)
Low quality reads	37,106 (0.27%)	9,399 (0.09%)
N% > 10% reads	722 (0.01%)	197 (0.00%)
Length <18nt and >30nt	3,576,896(26.37%)	3,162,932(28.78%)
5’ adapter contamine	6,440 (0.05%)	7,265 (0.07%)
3’ adapter null or Insert null	466,960 (3.44%)	329,058 (2.99%)
Clean reads	9,475,087(69.86%)	7,479,535(68.07%)
Unique reads	7,385,048 (54.45%)	6,653,378 (60.55%)

Table 2 summarizes the categories of unique reads. High levels of small RNA expression from rRNA and NAT genes were observed in both libraries. The number of miRNAs was more abundant in stem tissue as compared to the callus of tomato, mainly due to the high expression of sly-miR171, sly-miR396 and sly-miR397.

**Table 2:** Reads categories of two small RNA libraries derived from stem and callus.

Type	Number of reads (Percentage of reads)	
	Stem	Callus
Unique reads	7,385,048 (100%)	6,653,378 (100%)
Known miRNA	175634 (2.38%)	128569(1.93%)
rRNA	318166(4.31%)	295130(4.44%)
tRNA	0(0.00%)	0(0.00%)
snRNA	3493(0.05%)	4302(0.06%)
snoRNA	16964(0.23%)	18823(0.28%)
Repeat	525093(7.11%)	521017(7.83%)
NAT	648360(8.78%)	646505(9.72%)
Novel miRNA	28342(0.38%)	21589(0.33%)
TAS	16594(0.22%)	17806(0.27%)
Exon	203176(2.75%)	197359(2.96%)
Intron	357484(4.84%)	323339(4.86%)
Others^a^	5091742(68.95%)	4478939(67.32%)

^a^Others, refers to the number and proportion of the sRNA aligned to the reference sequences but not aligned to the known miRNA, ncRNA, repeat, NAT, novel miRNA, TAS, Exon and Intron.

### Identification of known miRNAs

**Fig 5:**
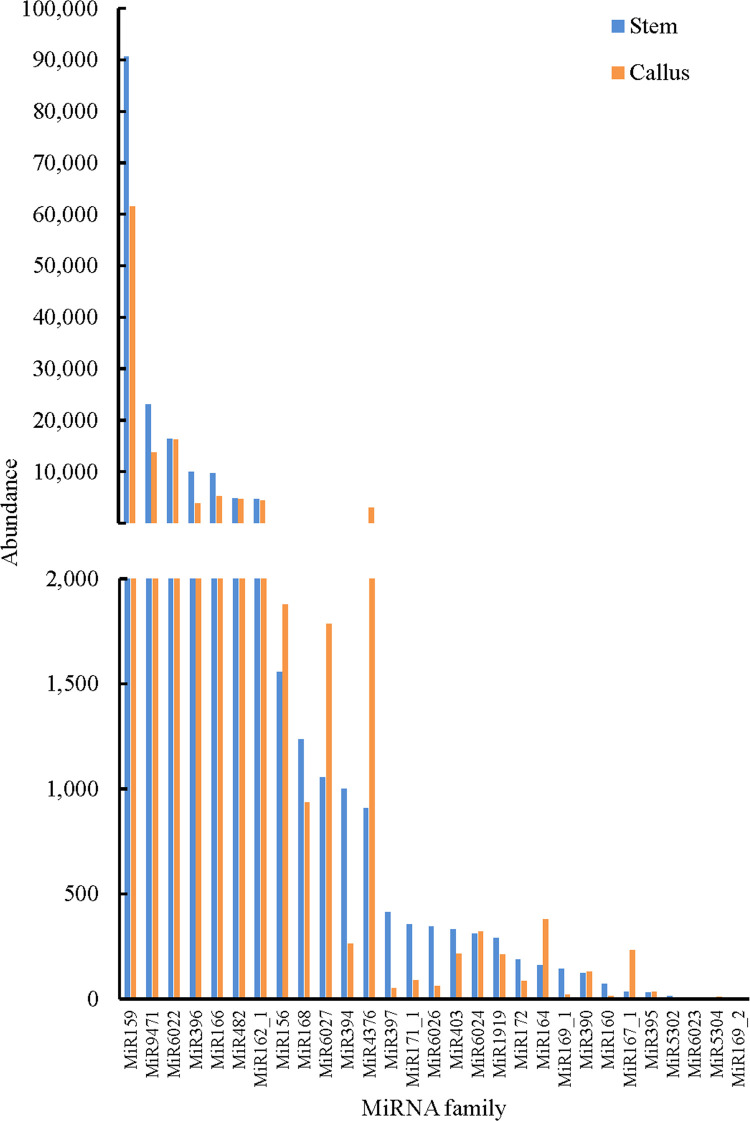
Reads of known miRNA families at stem and callus.

**Fig 6:**
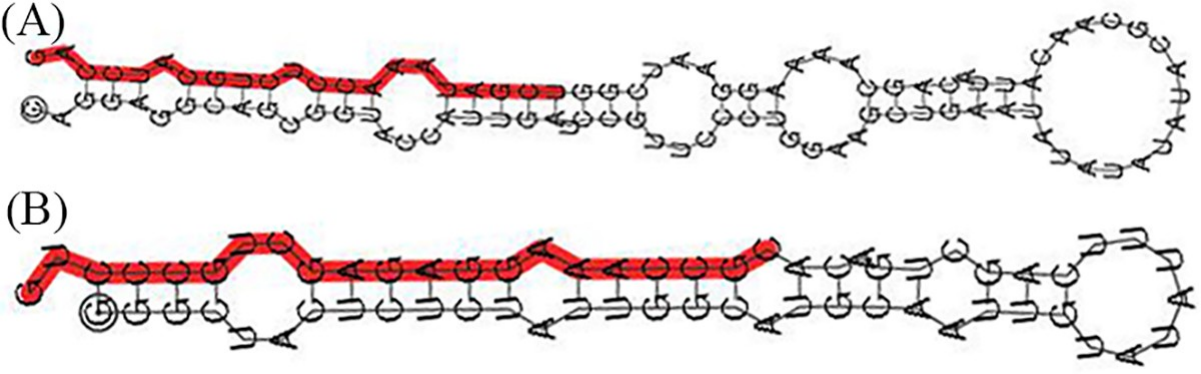
Secondary structure of identified miRNA precursors. The red protrusions are the mature sequences. (A) Known miRNA: sly-miR162; (B) Novel miRNA: novel 101.

To identify known miRNAs, sRNA sequences obtained from deep sequencing were contrasted to other currently annotated miRNAs of known mature plant species in miRBase. A total of 92 known miRNAs were identified, belonging to 29 miRNA gene families in the two sRNA libraries. Overall, 88 and 91 mature miRNAs were identified in the stem and callus tissues, respectively (S5 Table). As shown in Fig 5, the sly-miR159 family was the most abundantly expressed, while sly-miR9471, sly-miR6022, sly-miR396, and sly-miR166 families were moderately abundant. Furthermore, the secondary structures of known miRNAs are shown in Fig 6A and S1 Fig.

### Predicted novel miRNAs

Unannotated miRNAs were used to predict novel miRNAs. We identified 91 novel miRNAs were identified in total, of which 82 were mapped in both libraries (S6 Table). The expression levels of novel miRNAs were distinctly different. Most of them showed comparatively low expression levels (63 novel miRNAs in stem samples and 68 novel miRNAs in callus had less than 120 raw reads). In contrast, two novel miRNAs (annotated as novel 1 and novel 9) in both libraries contained more than 1,000 reads. The most abundantly expressed novel miRNA was novel 1 with a total of 23,938 reads in both libraries. The predicted secondary structures of novel pre-miRNAs are showed in Fig 6B and S2 Fig.

### Identification of differentially expressed miRNAs

**Fig 7:**
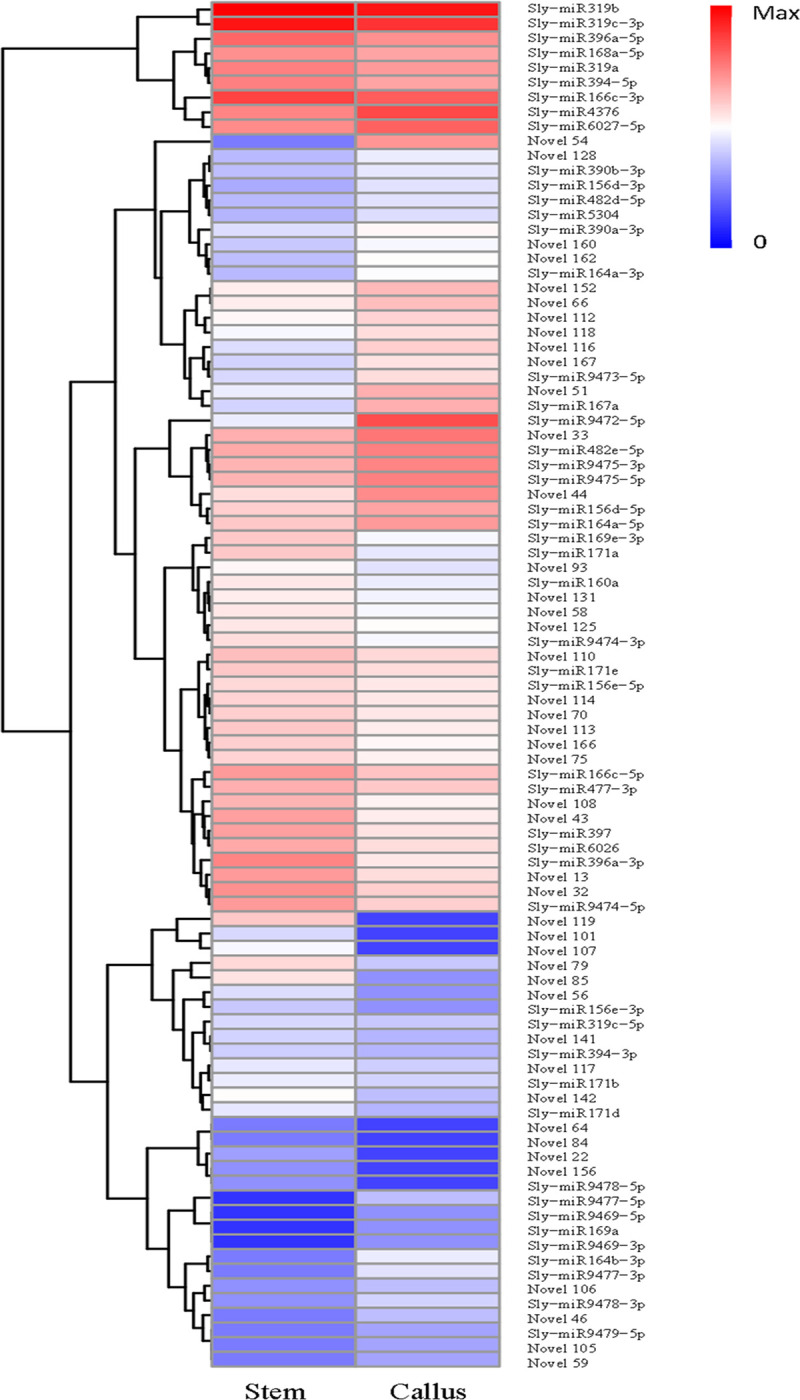
Cluster analyses of differentially expressed miRNAs. Red denotes highly expressed miRNAs, while blue denotes weakly expressed miRNAs. The color is from red to blue, indicating that log_10_ (TPM + 1) is from large to small.

**Fig 8:**
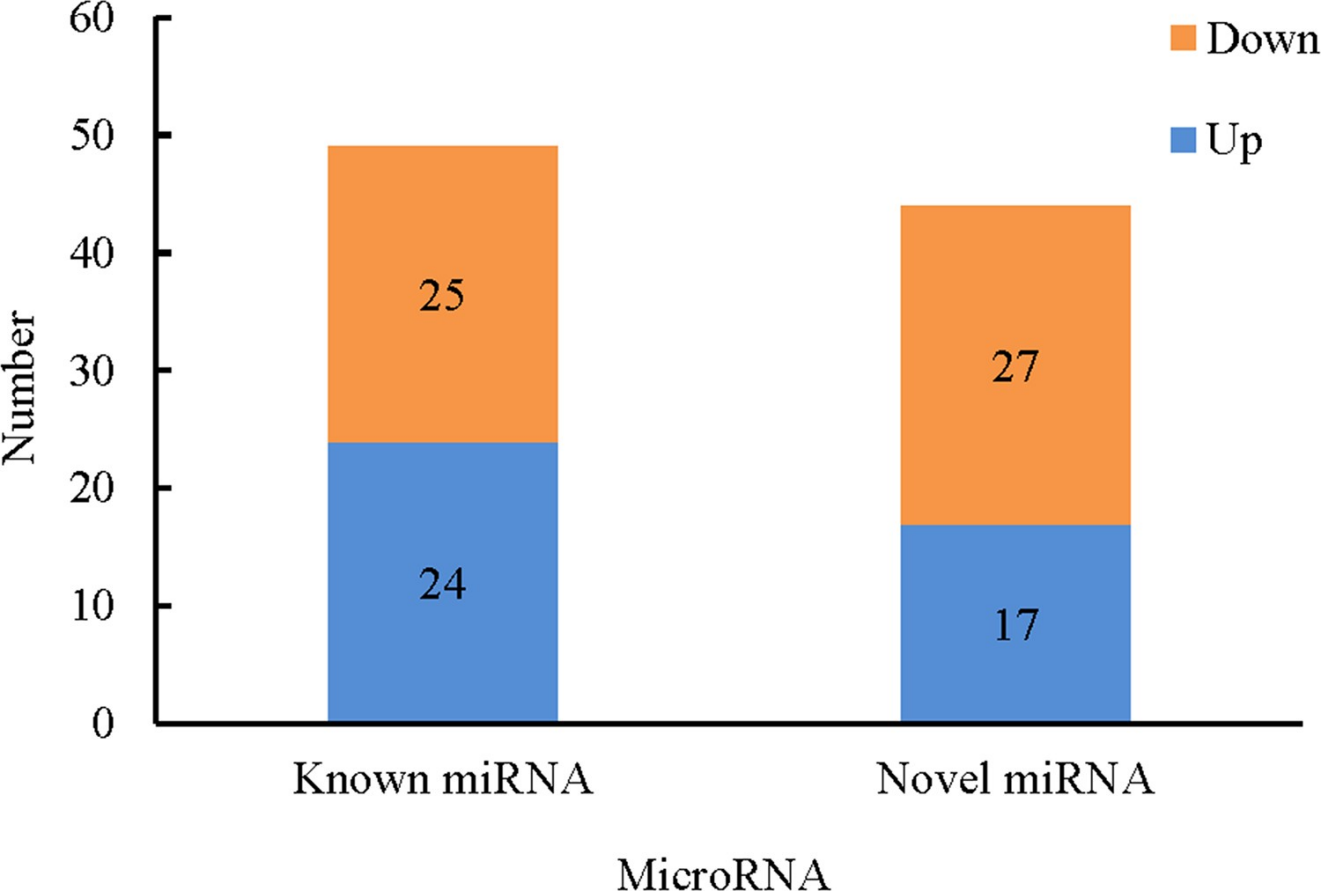
The number of known and novel up- and down- regulated miRNAs in callus vs. stem tissue.

**Fig 9:**
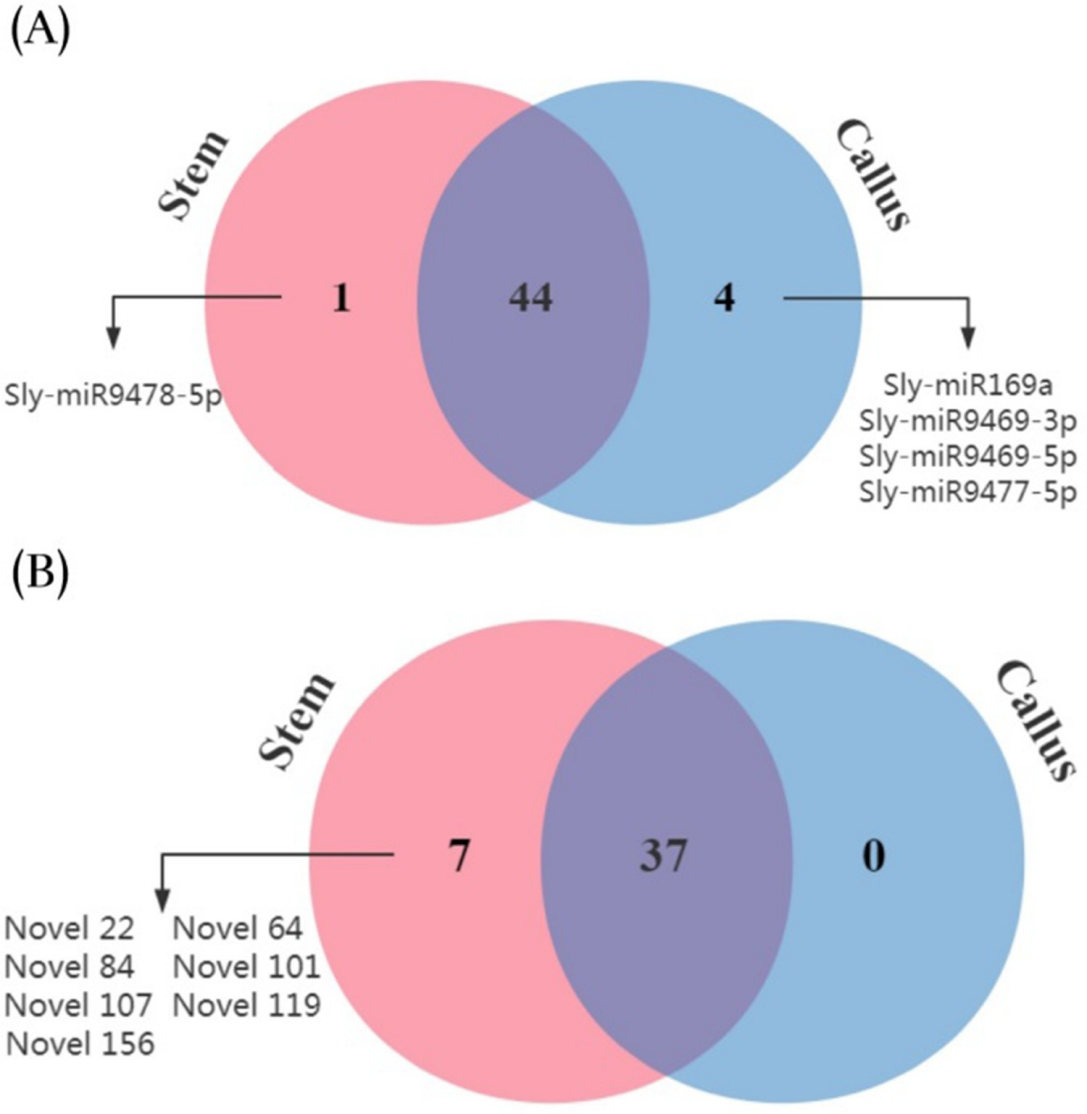
Venn diagram of the number of specifically expressed miRNAs at stem and callus. (A) Known miRNAs; (B) Novel miRNAs.

A total of 49 known and 44 novel miRNAs pertaining to the two libraries were expressed with significant differences with regards to log_2_ (callus/stem) (>1 or<–1) and P-value (<0.05) criteria (Fig 7) (S7 Table). For known miRNAs, 24 miRNAs were up-regulated and 25 miRNAs were down-regulated in callus vs. stem tissue samples. Among the novel miRNAs, 17 were up-regulated and 27 were down-regulated in callus vs. stem tissues (Fig 8). When miRNA distributions were assessed between the two libraries, 44 known defined miRNAs and 37 novel miRNAs were generally expressed in both libraries. The comparison of miRNA expression showed that 1 known and 7 novel miRNAs were expressed only in the stem, while 4 novel miRNAs were expressed solely in callus tissue, respectively (Fig 9).

The miRNAs with lower p-value include sly-miR166 and sly-miR397. The significantly down-regulated expression of sly-miR166 in callus cells could be related to its role in promoting callus formation by down-regulating homeodomain leucine zipper class III (HD-ZIP III) levels [43–45]. The most significantly down-regulated gene in callus tissue was sly-miR397, which is known to play an important role in the accumulation of laccases during callus formation [46,47].

**Fig 10:**
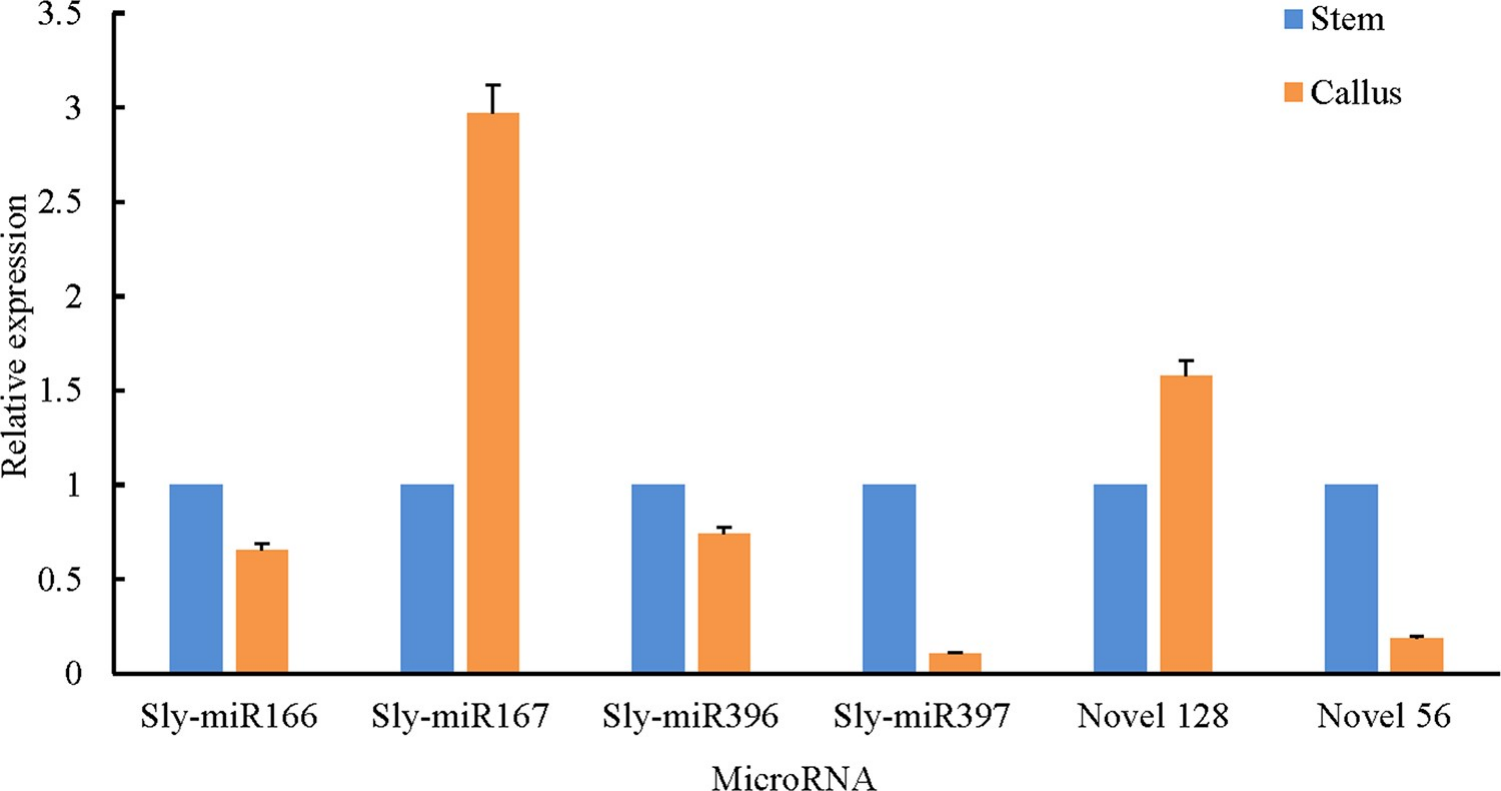
The relative expression levels of 6 (four known and two novel) miRNAs by qRT-PCR.

To confirm miRNAs expression levels in stem and callus and verify the deep-sequencing results, four known and two novel miRNAs were selected randomly for qRT-PCR. These miRNAs expression patterns resembled the deep-sequencing results, suggesting that sRNA sequencing data were reliable (Fig 10).

The bars represents the relative expression and standard deviation of the 6 miRNAs. qRT-PCR value of miRNAs in stem was set to 1, and values of miRNAs in callus were scaled.

### Target prediction

To analyze the biological functions of differentially expressed miRNAs in stem and callus tissues, the psRobot software was used to predict target genes. Among 1186 predicted target genes, a total of 505 known and 6 novel differentially expressed miRNA target genes were identified (S8 Table). Functional annotations of BLAST analysis for predicted target genes indicated that these targets contained mRNA coding regions for zinc finger protein (sly-miR165, sly-miR164, sly-miR391, sly-miR394, sly-miR396, sly-miR477, sly-miR482, sly-miR6027, sly-miR9469, sly-miR9478, and sly-miR9479), and MYB (sly-miR156, sly-miR319, sly-miR9469, and sly-miR9478) protein. Furthermore, some miRNAs were found to target transcription factors, such as SQUAMOSA promoter binding protein-like gene (SPL) (sly-miR156) [48], Auxin response factors (ARFs) (sly-miR160) [18], HD-ZIP III (sly-miR166) [21], NAM (sly-miR164 and sly-miR9478) [49], and MADS (sly-miR396 and sly-miR9477) [50], which are all known to be involved in plant regeneration. Laccase(sly-miR397) was also important in the regulation of plant development and regeneration [22,23]. The target genes of some miRNAs specifically expressed in the callus were CCAAT-binding (sly-miR169a), zinc finger (sly-miR9469-3p and sly-miR9469-5p), SQUAMOSA promoter binding protein (SBP-box), MADS-box and K-box (sly-miR9477-5p). Interestingly, all the target genes of novel 46 (solyc05g015840.2, solyc12g038520.1, solyc10g078700.1, solyc05g015510.2, solyc05g012040.2, and solyc04g045560.2) were the same as those targeted by sly-miR156e-5p, sly-miR156d-5p, and sly-miR156a.

## Discussion

Recently great progress has been made in understanding the role of miRNAs in regulating the transitions between different development stage in plants, such as those from vegetative-to-reproductive, juvenile-to-adult and aerial stem-to-rhizome transitions [51–54]. In the present study, we demonstrate that miRNAs are involved in complex regulatory networks during stem-callus transition during *in vivo* regeneration of tomato. We identified a total of 183 miRNAs (92 conserved and 91 novel miRNAs) by next-generation sequencing. Previous studies on miRNAs, including Xu et al. [30] identified 289 known miRNAs and 1087 novel miRNAs in longan, while Wu et al. [26] reported 50 known and 45 novel miRNAs in citrus. Taken together, these data show that distinct types of miRNAs are expressed at different levels during the process of regeneration in different species.

Cytokinin triggers a complex gene expression program in plant tissue culture that results in adventitious shoot regeneration [55]. The current model for cytokinin signal transduction is a multi-step phosphorelay. First, Arabidopsis histidine kinase (AHKs), the cytokinin receptors in the plasma membrane, perceive the cytokinin signal triggering a multi-step phosphorelay. At the end of this pathway, B-ARR receives the phosphoryl group and becomes active. As transcription factors, B-type ARRs can activate the expression of cytokinin-responsive genes and A-type ARRs. Interestingly, the expression of A-type ARRs interferes with the function of B-type ARR proteins through a negative feedback loop [56,57]. Cytokinin thus plays a vital role during *in vitro* regeneration. It can not only induce adventitious buds alone, but also cooperate with auxin. Many studies have confirmed that miRNA regulate hormone signaling genes involved in regeneration.

### miRNA does not regulate *in vivo* regeneration through cytokinin synthesis

The content of trans-zeatin increased with the *in vivo* regeneration process in our study, which was related well with previous studies that 6-benzyladenine treatment increased the number of adventitious shoot amounts during *in vivo* regeneration of tomato [58]. Furthermore, our findings are also supported by other studies which used zeatin as the only exogenous hormone during *in vitro* regeneration of tomato [59–61]. So we considered that cytokinin played a role in *in vivo* regeneration of tomato.

There was no significant difference in the regeneration of wound between lovastatin with and without, which indicated that cytokinin could not synthesized at the wound. Meanwhile, the target genes of significant differential expression miRNA were not found to be related to cytokinin synthesis. Therefore, we suggested that miRNAs were not involved in cytokinin synthesis but participated in cytokinin signal transduction during *in vivo* regulation.

### miR156/SPL module involved in callus formation by regulating cytokinin signaling pathway

Siddiqui et al. [62] summarized the most common expressed miRNAs during SEG in 11 economically plants. Of these, miRNA156 was found to be most frequently detected in six of the 11 plant species tested. Sequences of miR156 were highly conserved in plants [63]. In the present study, sly-miR156d-5p and sly-miR156e-5p were newly identified and shown to be expressed differently in stem and callus tissue. Promoter binding protein (SBP) domain SQUAMOSA was predicted to be one of the targets of sly-miR156d-5p and sly-miR156e-5p. SBP domain proteins, putative plant-specific transcription factor gene families, have been shown to participate in various plant biological processes and to be involved in vegetative-to-reproductive phase transition [64–66], pollen sac development [67], gibberellins (GAs) signaling network [68] and establishment of lateral meristems [69]. As SBP-box gene family members, 10 of 16 *SPL* genes were shown to be targets of miR156 in *Arabidopsis*, while 10 of the 15 *SPL* genes were proposed to be targets of miR156 in citrus [70,71]. The function of the miR156-SPLs module was confirmed to be crucial in callus production in citrus *in vitro* callus through targeted inhibition of miR156-targeted SPLs and over-expression of csi-miR156a [20]. Therefore, differential expression levels of miR156 during tomato callus generation in the present study, suggest that is likely to play an important role in *in vivo* regeneration.

Zhang et al. [72] demonstrated that miR156 participates in regulation of shoot regeneration *in vitro*. miR156 expression gradually increases with age and suppresses the expression of its target *SPL* genes. Down-regulated SPLs attenuate cytokinin signaling by binding to the B-type Arabidopsis response regulators (ARR) transcription factor. The data presented here show that cytokinin levels increase during *in vivo* regeneration in tomato. However, sly-miR156d-5p and sly-miR156e-5p were found to be up- and down-regulated, respectively. Thus, the regulation of miR156-SPL-ARR module during *in vivo* callus formation and shoot regeneration in tomato needs to be further investigated.

### IAA level regulated by miR166 in callus formation

Low expression levels of sly-miR166c-5p and sly-miR166c-3p were observed during the change from stem to callus stages in this study. Previous research has shown that miR166, together with miR156 and miR396 were down-regulated during callus formation from tea plant stem explants [73]. miR166 was identified to target Class III homeodomain leucine zipper (HD-Zip III) gene family of transcription factors, including REVOLUTA (REV), PHABULOSA (PHB), PHAVOLUTA (PHV), CORONA (CNA), and ATHB8 in *Arabidopsis* [43]. HD-ZIP III proteins play an important role in plant regeneration by regulating the differentiation of stem cells and the establishment of shoot apical meristem (SAM) and RAM [74–76].

More recently, REV was demonstrated to activate genes upstream of several auxin biosynthesis, transport, and response genes. Brandt et al. [77] identified that REV targeted the auxin biosynthetic enzymes TAA1 and YUCCA5(YUC5), and directly affected the levels of free auxin. In *Arabidopsis*, loss-of-function mutants of REV showed lower expression levels of the PIN1 and PIN2 auxin transporters and reduction in the tip-to-base transport of auxin [78]. Additionally, REV function is necessary for polar auxin transport in the shoot [79]. Li et al. [45,80,81] demonstrated that over-expression of La-miR166a and down-regulated of LaHDZIP31-34 genes results in different IAA levels in pro-embryogenic masses of *L*. *leptolepis*. The authors speculated that La-miR166 targeted HD-ZIP III genes likely regulate auxin biosynthesis and response genes. Overall, these results indicated the complex regulaory relationships between miR166 and plant development. Further, Ma et al. [54] reviewed five key microRNAs involved in developmental phase transitions in seed plant, and miR166 was one of them.

### Other miRNAs related to the phytohormone signaling during *in vivo* regeneration

**Fig 11:**
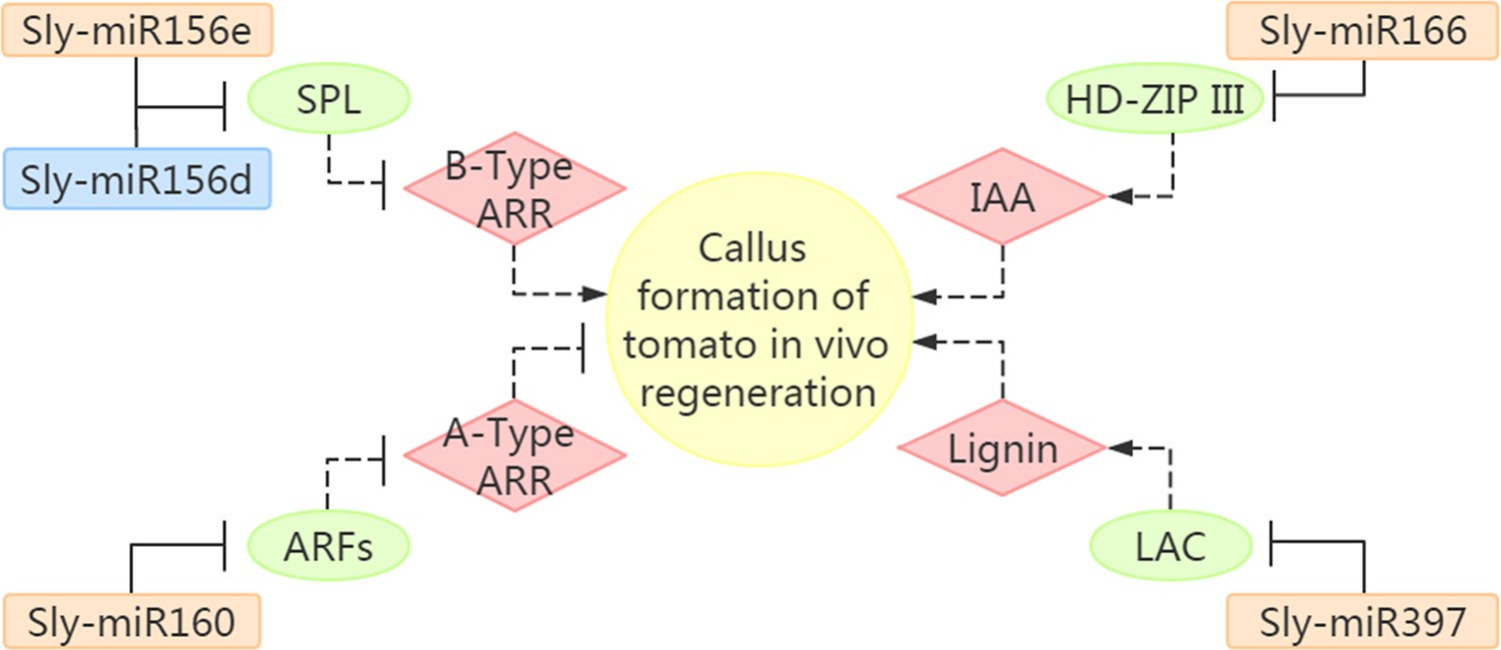
Genetic networks of callus formation during *in vivo* regeneration of tomato regulated by miRNA-target modules together with their downstream targets.

There is no doubt that auxin signaling and transport is a versatile trigger of plant developmental changes incluing regeneration [82]. Based on a number of previous studies, which focussed on miRNAs involved in regulation of the auxin signaling, in *Arabidopsis*, miR393 was shown to contribute to SE, leaf development and antibacterial resistance by repressing auxin signaling [83–85]. Two auxin response factors genes, *ARF6* and *ARF8*, are targeted by miR167 [86]. During callus formation, miR160 was defined as a key repressor by modulating the interplay between auxin and cytokinin. The callus initiation was repressed by over-expression of miR160 or reduced expression of its target *ARF10*. *ARF10* can inhibit cytokinin signaling A-type genes *ARR15* [87]. Although A-type genes *ARR15* and *ARR7* were identified to inhibit callus formation, those of the B-type genes *ARR1* and *ARR21* can enhance its initiation [88,89] (Fig 11). Although the expression of miR160 was low, in many previous studies, the read of miRNA greater than 10 was regarded as expression [2,27,90]. Therefore, we speculated that miR160 may play a role in tomato *in vivo* regeneration.

Arrows represent activation, while lines with a bar represent repression. The solid lines represent the results predicted by this study, and the dotted lines represent the results from references. The up-regulated miRNAs are shown in the blue box, while the down-regulated miRNAs are shown in orange ones. miRNA targets are shown in green oval frames. Cell proliferation relied not only on high levels of auxin but also on low level of cytokinin during in vitro callus induction in *Arabidopsis* [91]. This study showed the down-regulation of sly-miR160 and low concentrations of cytokinin in callus were crucial in callus formation. We predict that a similar interplay between microRNA/phytohormone levels may exist between *in vivo* and *in vitro* regeneration in tomato.

### miR397 repressed callus formation through inhibition of laccases expression

The expression of Sly-miR397 was most significantly down-regulated in callus tissue. MiR397 has been validated to target laccases (*LAC2* and *LAC17* in this study), a group of polyphenol oxidases [46,92]. In higher plants, laccases are associated with lignin and xylem synthesis, and are proposed to play a role in secondary cell wall thickening [23,93,94]. Lignin is an essential component of plant secondary cell walls, which influence plant growth and differentiation [95]. Previous studies indicated that callus tissue first contains lignified parenchyma cells, followed by the formation of short vessels and traumatic resin ducts after plant injury, and the induction of vessels requires the involvement of lignin [47,96]. Overall, we predict that low expression levels of *Sly-miR397* in callus tissue permits the accumulation of laccases, leading to the increase of lignin deposition within the callus.

## Conclusion

We used a new model system to study the dynamic changes in trans-zeatin levels and the regulatory patterns of miRNA expression during *in vivo* regeneration of tomato. The significant changes in trans-zeatin levels at 0, 9, 12, 15, 18, 21, 24 and 30 d after decapitation proves that trans-zeatin plays a crucial role during *in vivo* regeneration in tomato. However, the treatment with excess lovastatin on the cut surface of tomato stems did not inhibit callus formation, which indicated that *de novo* biosynthesis of cytokinin did not occur in the cut surface of tomato stems. A total of 92 known and 91 novel miRNAs were identified from the stem explant and the callus regenerated from the cutting surfaces after decapitation, respectively, of which 49 known and 44 novel miRNAs exhibited differential expression between the two libraries. In addition, a total of 505 known miRNA target genes and 6 novel miRNA target genes were further identified. We predict that these differentially expressed miRNAs and their relevant target genes play an important role in callus formation during *in vivo* regeneration of tomato. Among these, sly-miR156, sly-miR160, sly-miR166, and sly-miR397 are predicted to be involved in callus formation during *in vivo* regeneration of tomato by targeting SPL, HD-ZIP III, ARFs, and LAC proteins, as well as by regulating cytokinin, IAA, and laccase levels. The findings of this study provide a useful resource for further investigation on callus formation during *in vivo* regeneration of tomato.

## Supporting information

S1 Fig The secondary structures of known miRNAs. The whole sequences are miRNA precursors, and the red prominent parts are the mature sequences.
(PDF)

S2 Fig The secondary structures of novel pre-miRNAs. The whole sequences are miRNA precursors, and the red prominent parts are the mature sequences.
(PDF)

S1 Table Primers used in this study for qRT-PCR. (DOCX)

S2 Table The content of trans-zeatin during *in vivo* regeneration of tomato micro-TOM. ^a^ Trans-zeatin weight, calculated by the standard curve y = 0.0651x–3.3111, R2 = 0.9963 of HPLC. ^b^ Trans-zeatin content, obtained by dividing the weight by the fresh weight of the samples.
(DOCX)

S3 Table The number of regenerated adventitious shoots in lovastatin and control treated tomato plants. The samples were the cutting surface of stem at 0, 30, 37, 44, 51 and 58 d after decapitation, 10 tomato plants were contained in each sample.
(DOCX)

S4 Table Quality of raw reads of two sRNA libraries produced from stem and callus. ^a^ Q20, The percentage of bases with Phred value greater than 20 in the total bases. ^b^ Q30, The percentage of bases with Phred value greater than 30 in the total bases.
(DOCX)

S5 Table Detailed information of known miRNAs in two small RNA libraries derived from stem and callus. (DOCX)

S6 Table Novel miRNAs of two small RNA libraries derived from stem and callus. (DOCX)

S7 Table Differentially expressed known and novel miRNAs during callus formation of tomato *in vivo* regeneration. (DOCX)

S8 Table Target genes for differentially expressed known and novel miRNAs. (DOCX)
