# Supplementary material for: miRNA expression profiling and zeatin dynamic changes in a new model system of in vivo indirect regeneration of tomato
Source: PLoS One. 2020 Dec 17;15(12):e0237690. doi: 10.1371/journal.pone.0237690 (PMC7745965; doi:10.1371/journal.pone.0237690)

sly-miR156a_sly-MIR156a

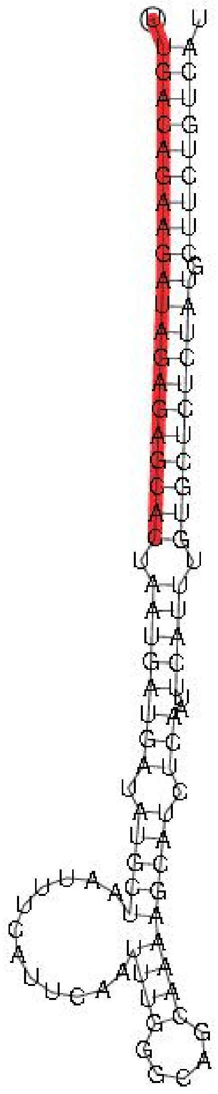

sly-miR156d-3p_sly-MIR156d

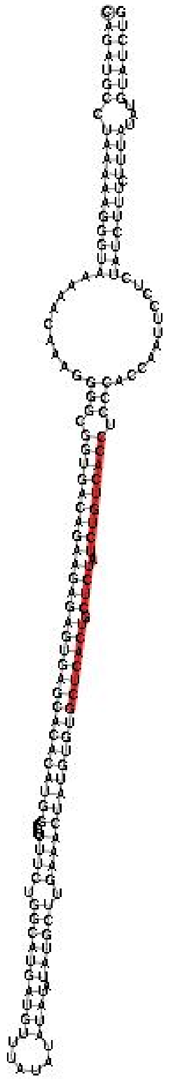

sly-miR156d-5p_sly-MIR156d

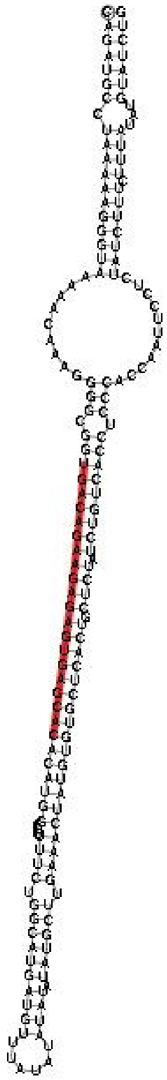

sly-miR156e-3p_sly-MIR156e

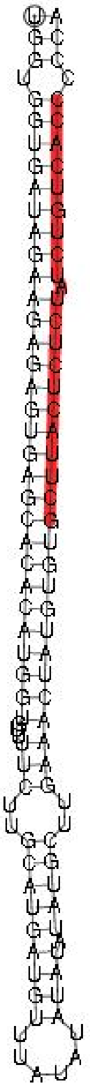

sly-miR156e-5p_sly-MIR156e

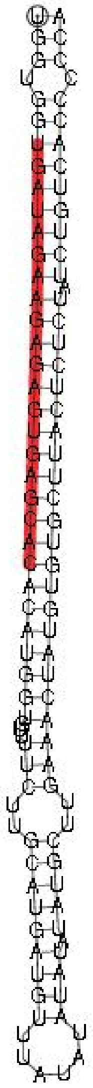

sly-miR159_sly-MIR159

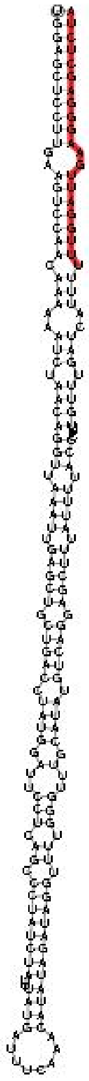

sly-miR160a_sly-MIR160a

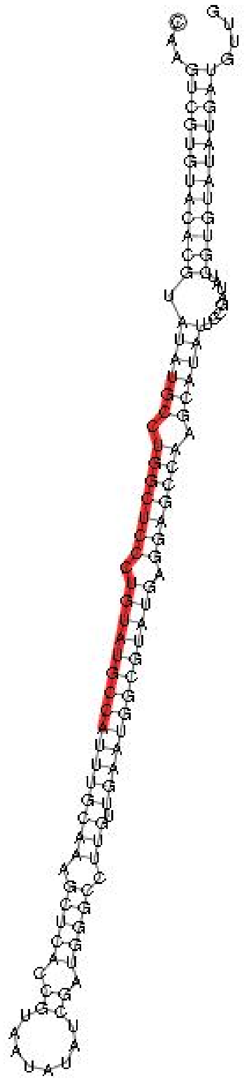

sly-miR162_sly-MIR162

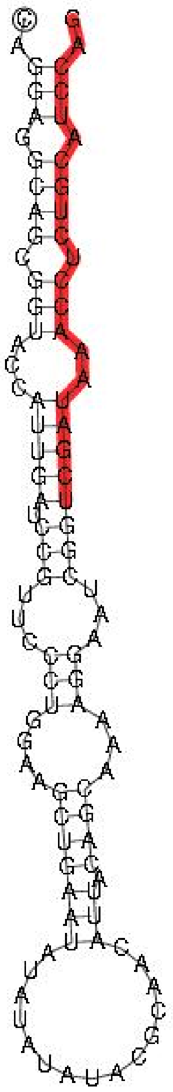

sly-miR164a-3p_sly-MIR164a

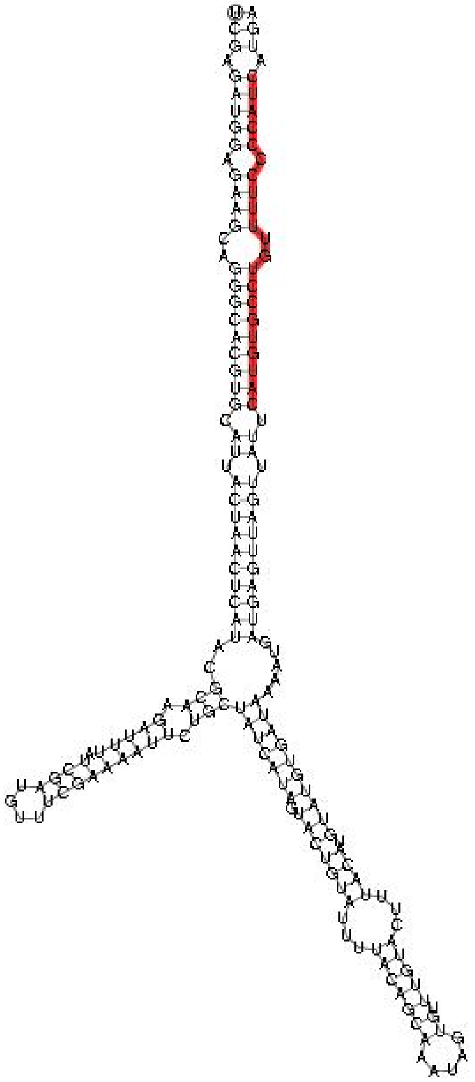

sly-miR164a-5p_sly-MIR164a

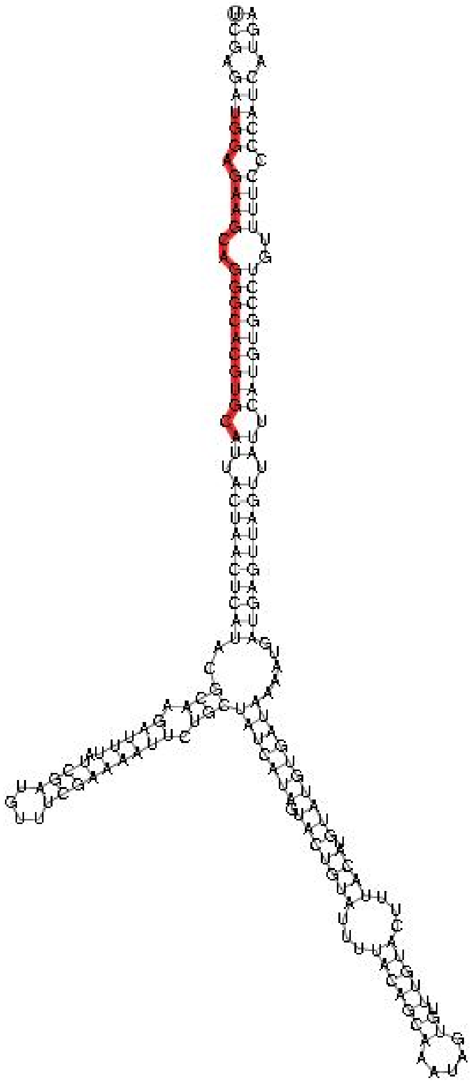

sly-miR164b-3p_sly-MIR164b

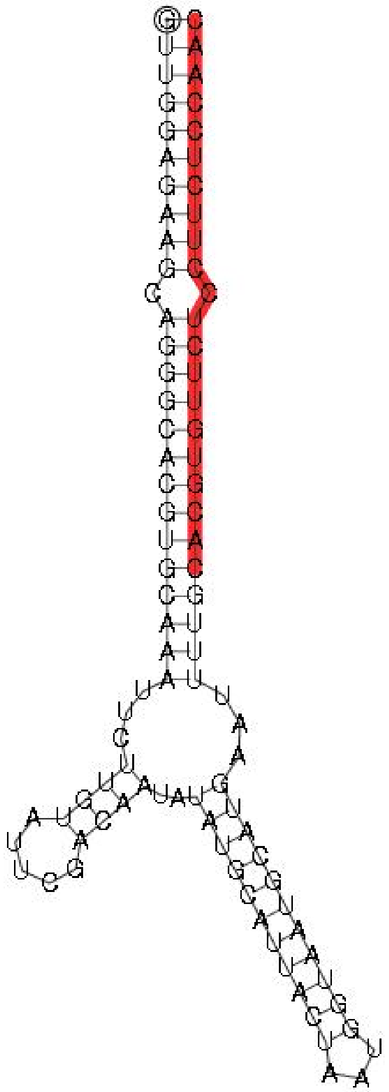

sly-miR166a_sly-MIR166a

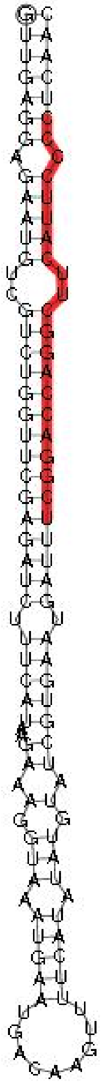

sly-miR166c-3p_sly-MIR166c

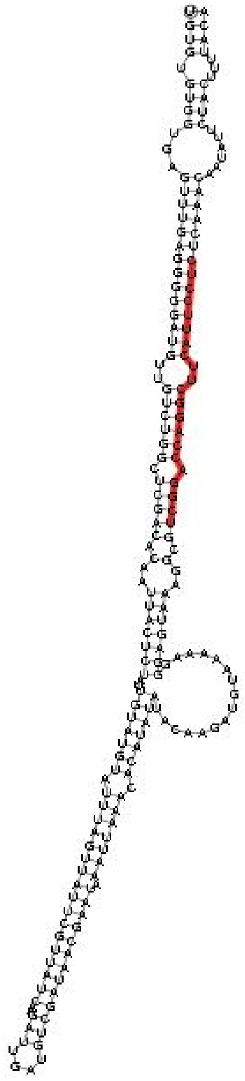

sly-miR166c-5p_sly-MIR166c

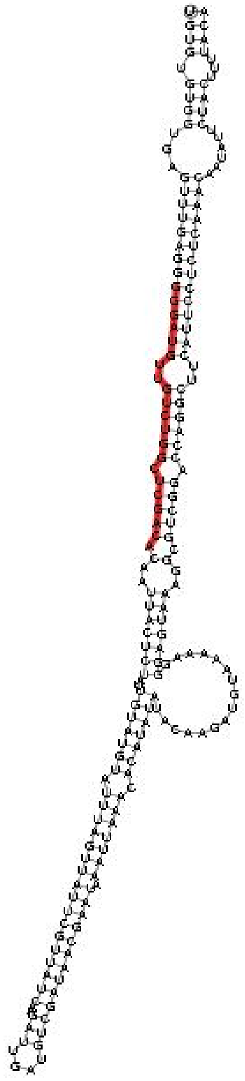

sly-miR167a_sly-MIR167a

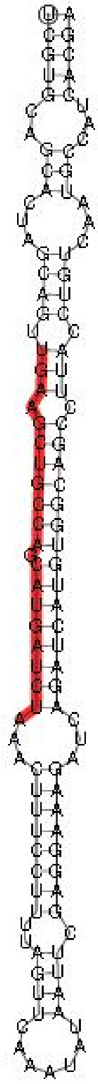

sly-miR167b-3p_sly-MIR167b

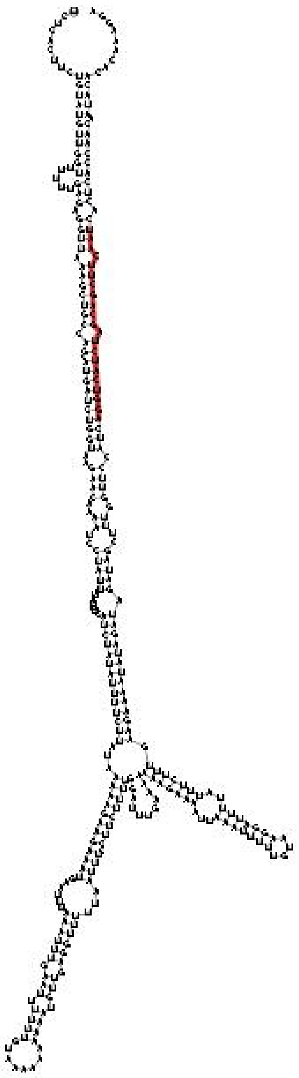

sly-miR167b-5p_sly-MIR167b

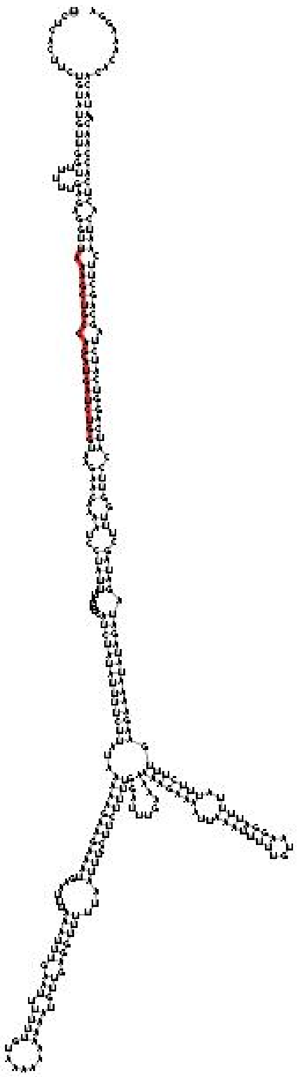

sly-miR168a-3p_sly-MIR168a

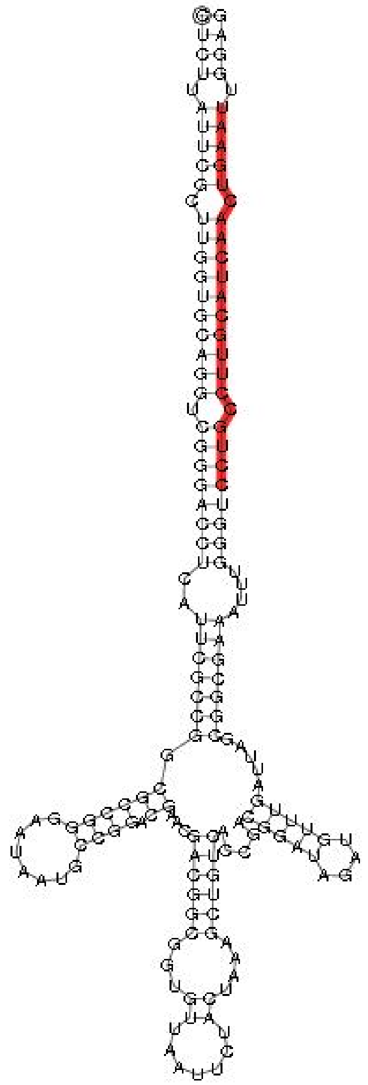

sly-miR168a-5p_sly-MIR168a

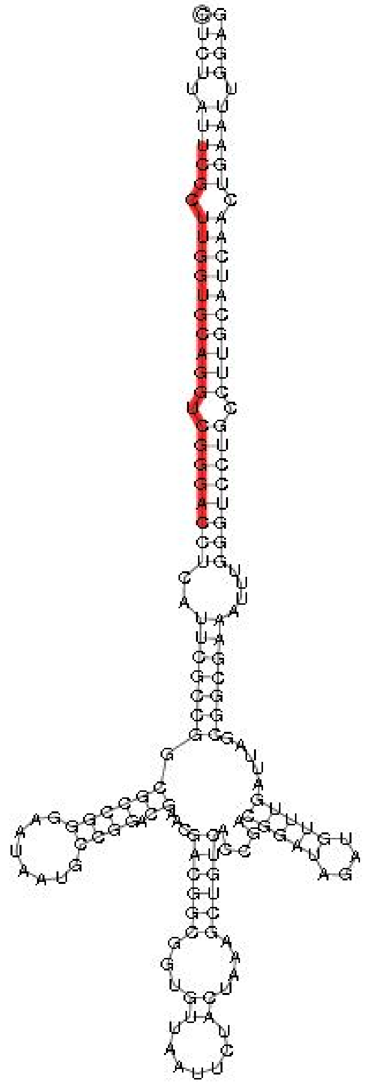

sly-miR168b-3p_sly-MIR168b

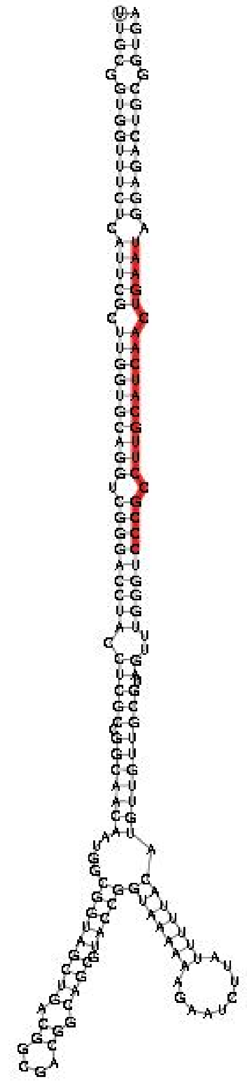

sly-miR169a_sly-MIR169a

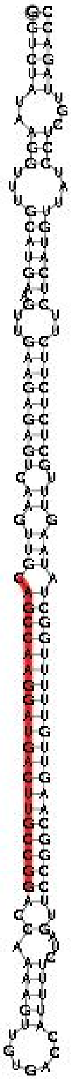

sly-miR169e-3p_sly-MIR169e

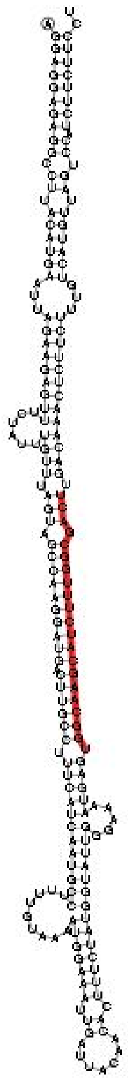

sly-miR171a_sly-MIR171a

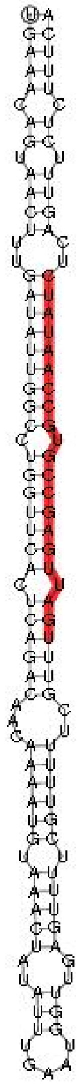

sly-miR171b_sly-MIR171b

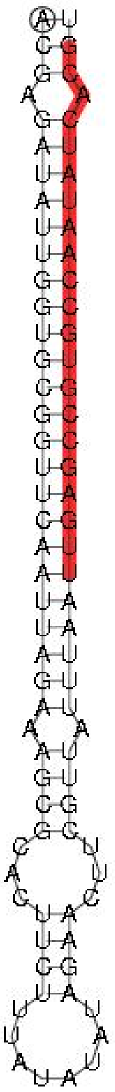

sly-miR171c_sly-MIR171c

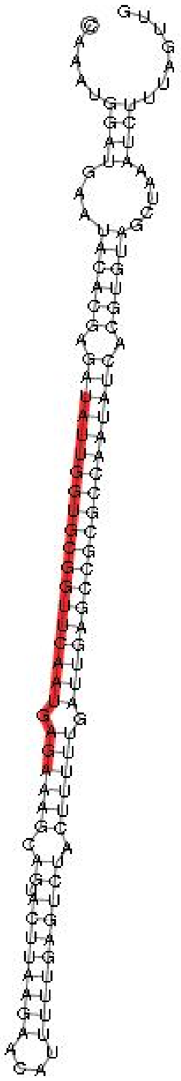

sly-miR171d_sly-MIR171d

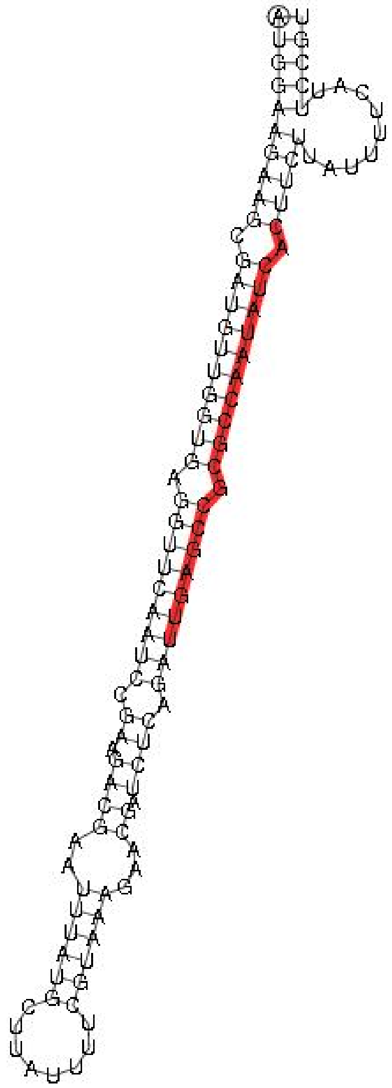

sly-miR171e_sly-MIR171e

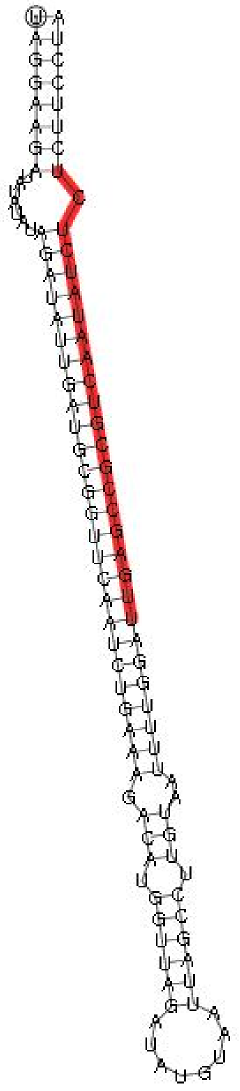

sly-miR172a_sly-MIR172a

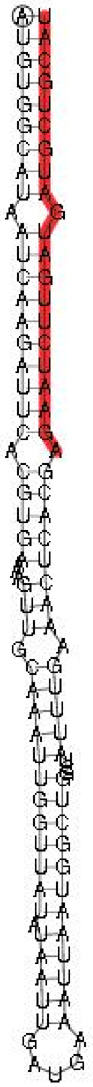

sly-miR319a_sly-MIR319a

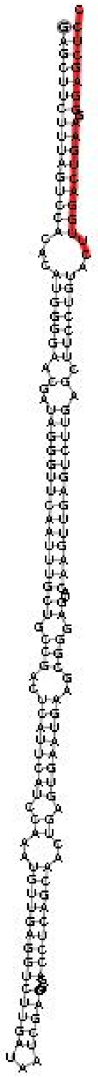

sly-miR319b_sly-MIR319b

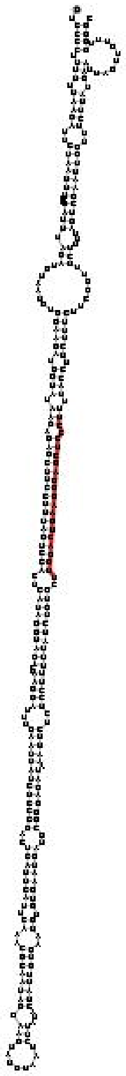

sly-miR319c-3p_sly-MIR319c

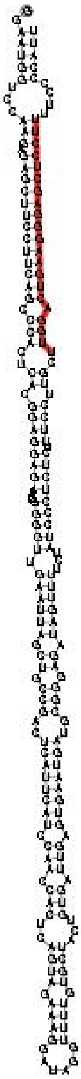

sly-miR319c-5p_sly-MIR319c

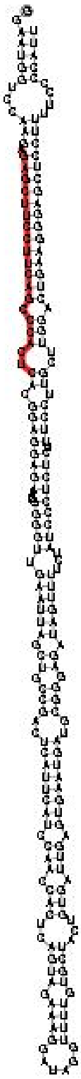

sly-miR390a-3p_sly-MIR390a

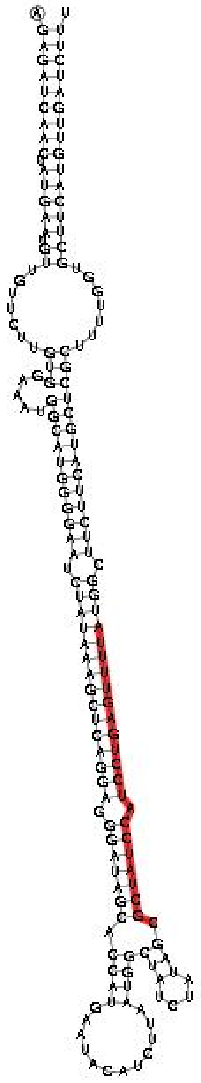

sly-miR390a-5p_sly-MIR390a

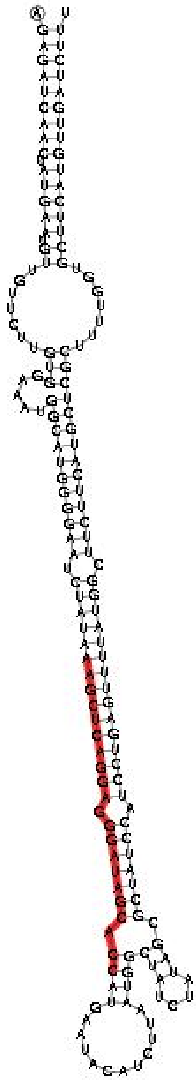

sly-miR390b-3p_sly-MIR390b

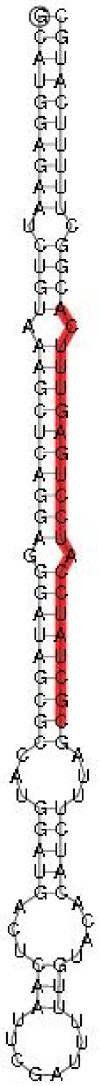

sly-miR390b-5p_sly-MIR390b

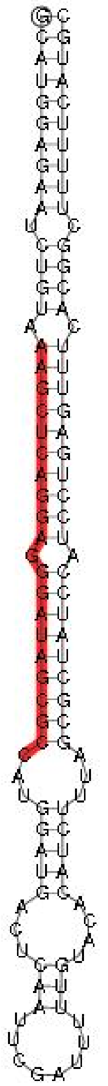

sly-miR394-3p_sly-MIR394

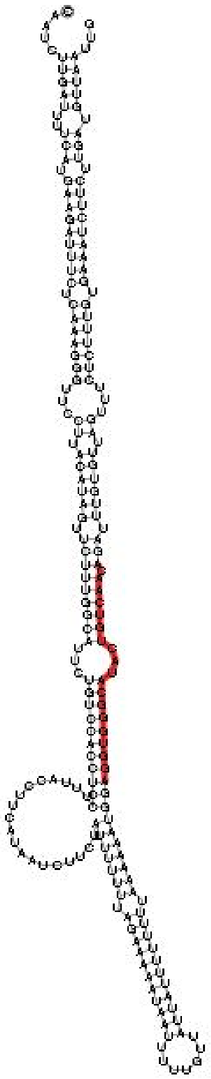

sly-miR394-5p_sly-MIR394

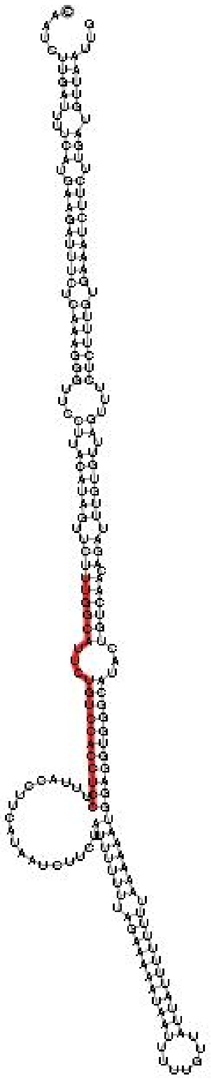

sly-miR395a_sly-MIR395a

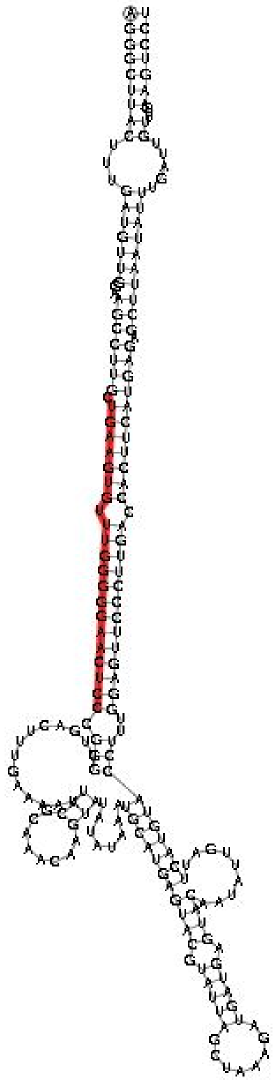

sly-miR396a-3p_sly-MIR396a

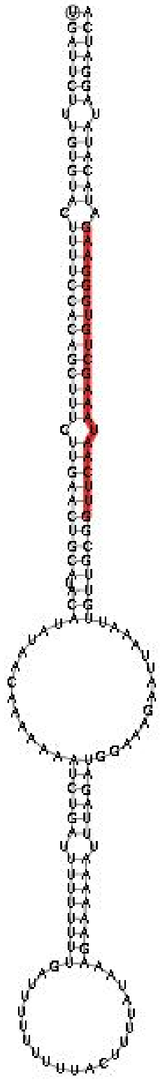

sly-miR396a-5p_sly-MIR396a

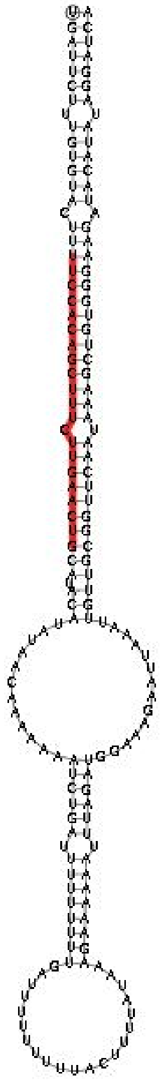

sly-miR396b_sly-MIR396b

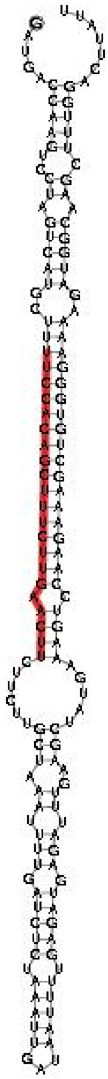

sly-miR397_sly-MIR397

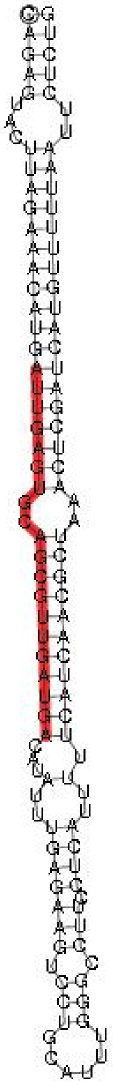

sly-miR403-3p_sly-MIR403

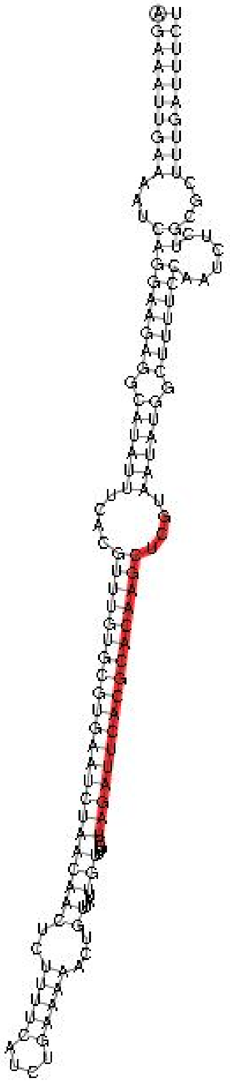

sly-miR403-5p_sly-MIR403

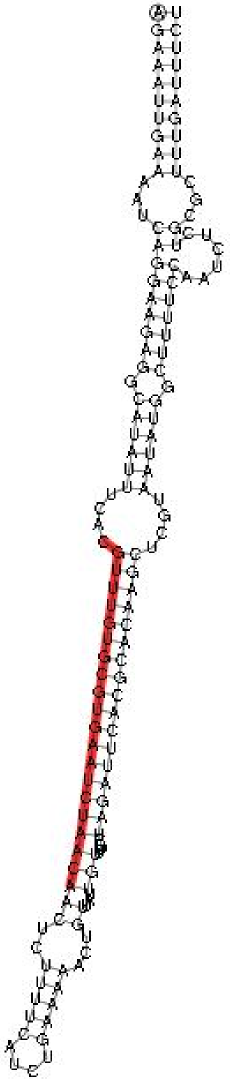

sly-miR477-3p_sly-MIR477

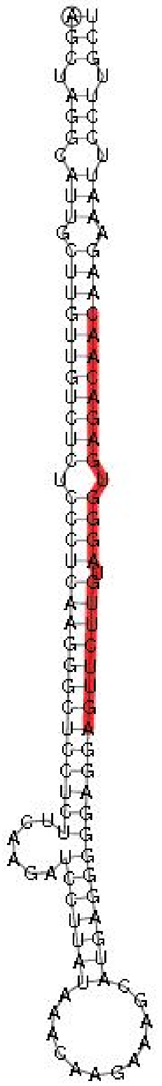

sly-miR477-5p_sly-MIR477

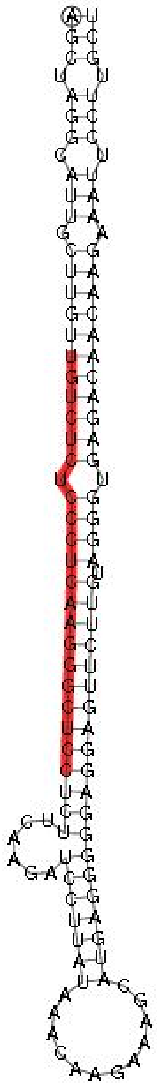

sly-miR482a_sly-MIR482a

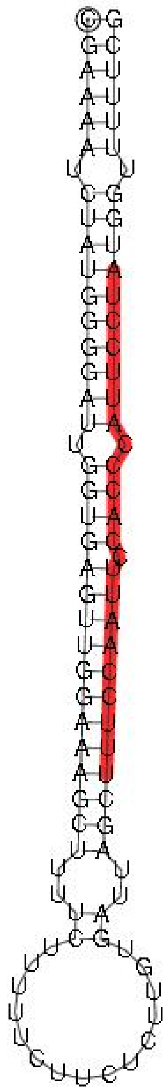

sly-miR482b_sly-MIR482b

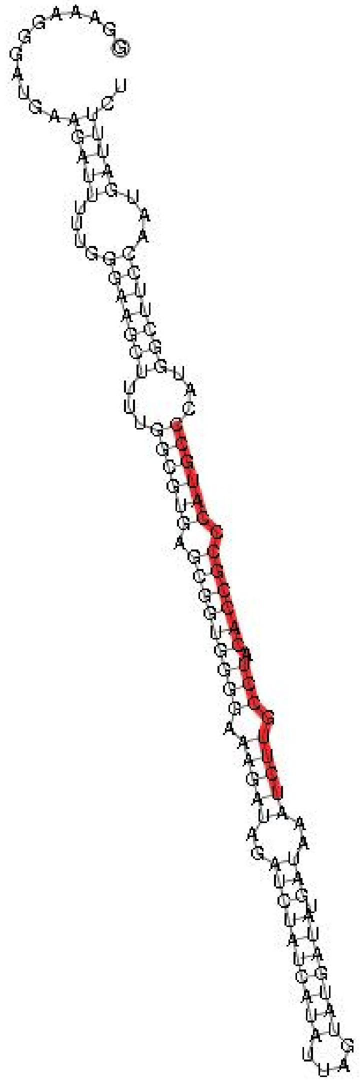

sly-miR482c_sly-MIR482c

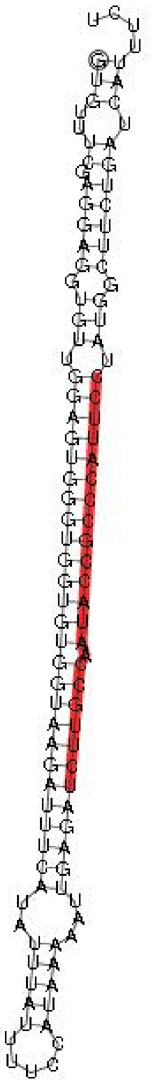

sly-miR482d-3p_sly-MIR482d

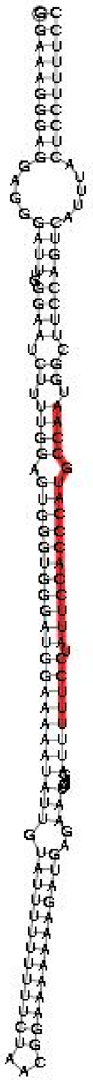

sly-miR482d-5p_sly-MIR482d

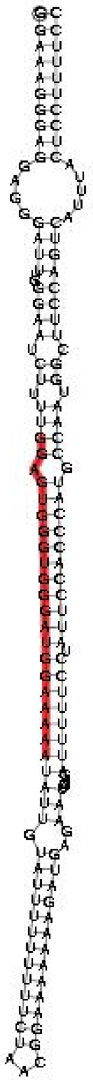

sly-miR482e-3p_sly-MIR482e

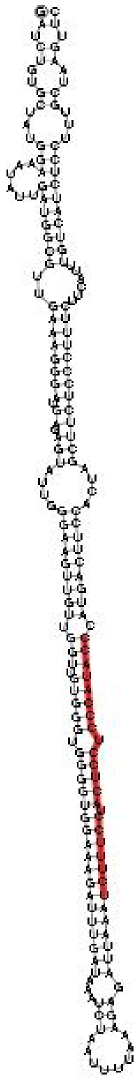

sly-miR482e-5p_sly-MIR482e

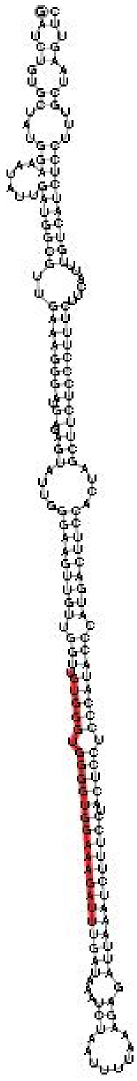

sly-miR1916_sly-MIR1916

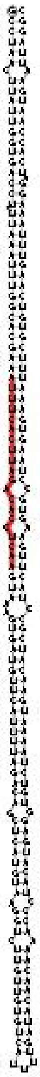

sly-miR1917_sly-MIR1917

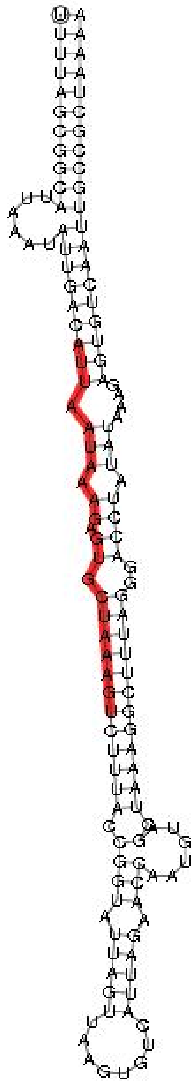

sly-miR1918_sly-MIR1918

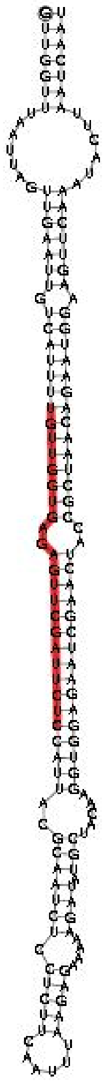

sly-miR1919a_sly-MIR1919a

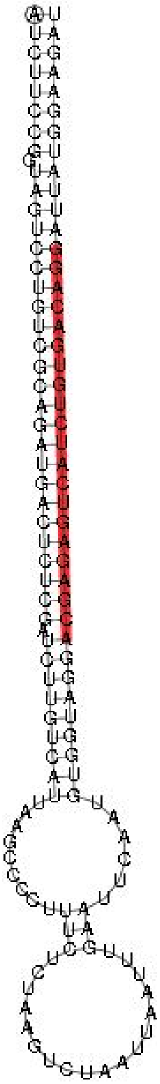

sly-miR1919c-5p_sly-MIR1919c

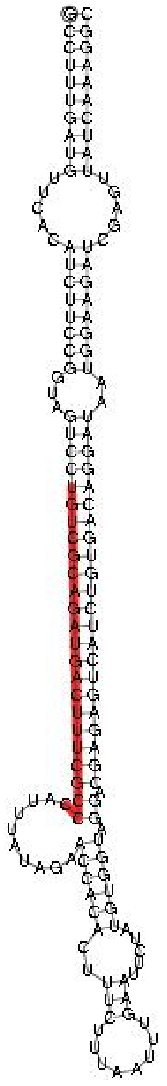

sly-miR4376_sly-MIR4376

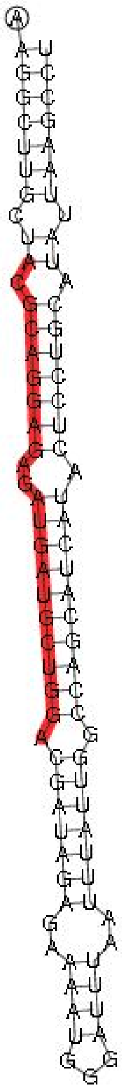

sly-miR5300_sly-MIR5300

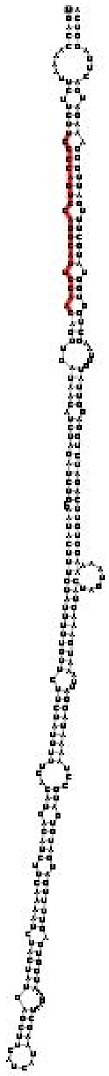

sly-miR5302b-5p_sly-MIR5302b

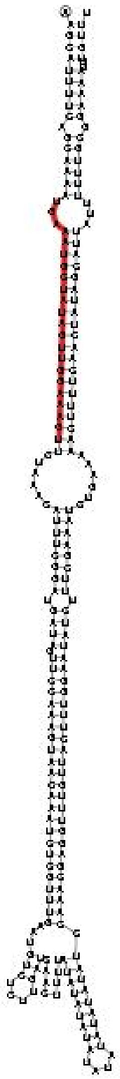

sly-miR5304_sly-MIR5304

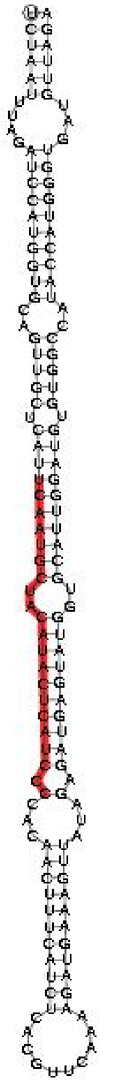

sly-miR6022_sly-MIR6022

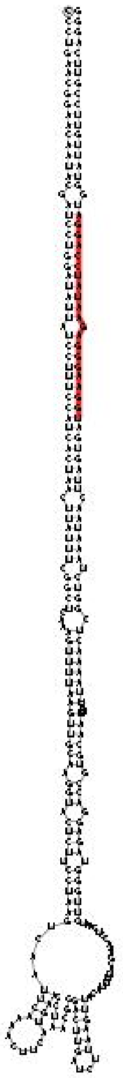

sly-miR6023_sly-MIR6023

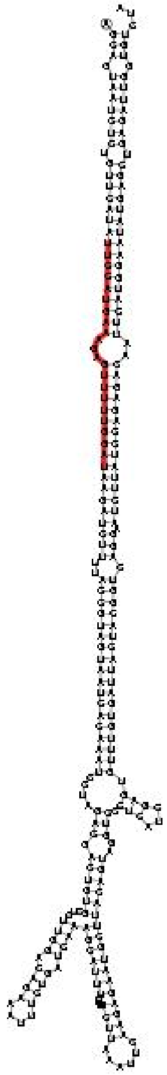

sly-miR6024_sly-MIR6024

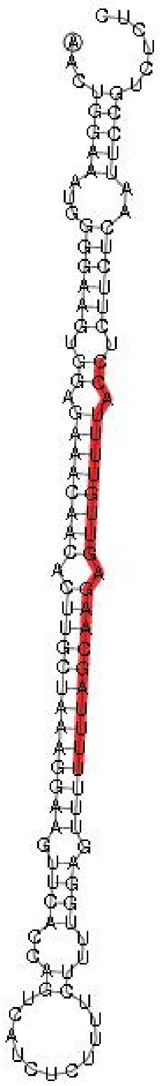

sly-miR6026_sly-MIR6026

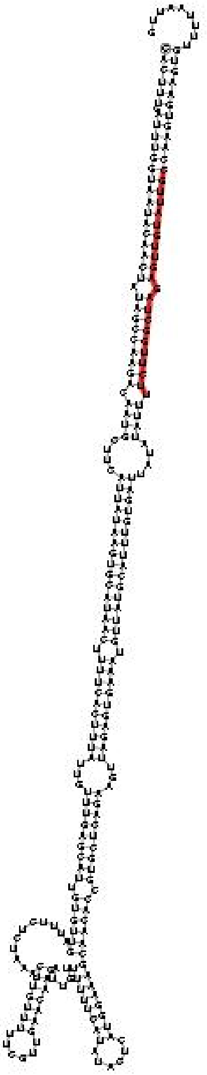

sly-miR6027-3p_sly-MIR6027

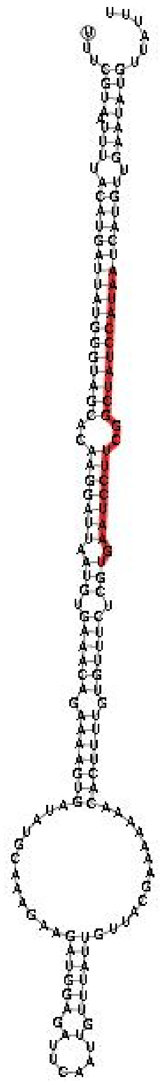

sly-miR6027-5p_sly-MIR6027

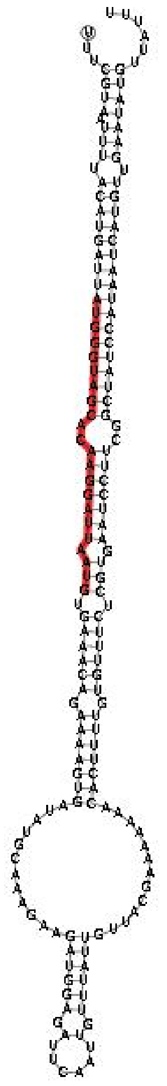

sly-miR9469-3p_sly-MIR9469

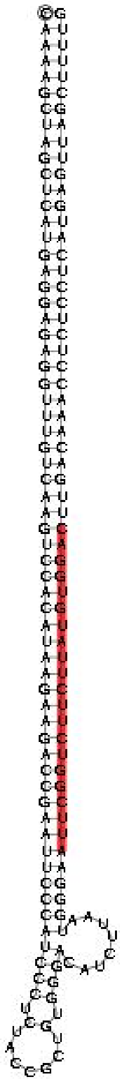

sly-miR9469-5p_sly-MIR9469

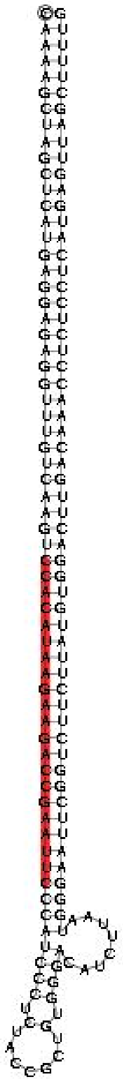

sly-miR9470-3p_sly-MIR9470

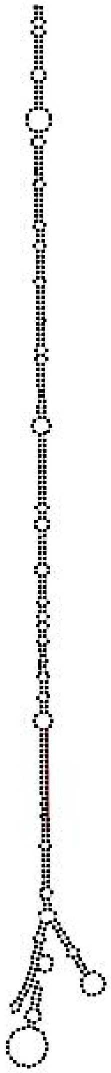

sly-miR9470-5p_sly-MIR9470

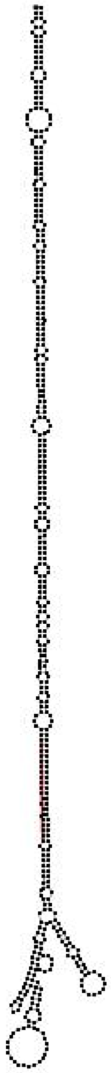

sly-miR9471a-3p_sly-MIR9471a

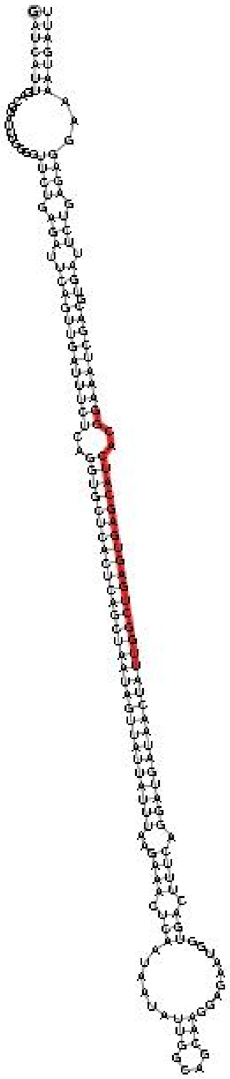

sly-miR9471a-5p_sly-MIR9471a

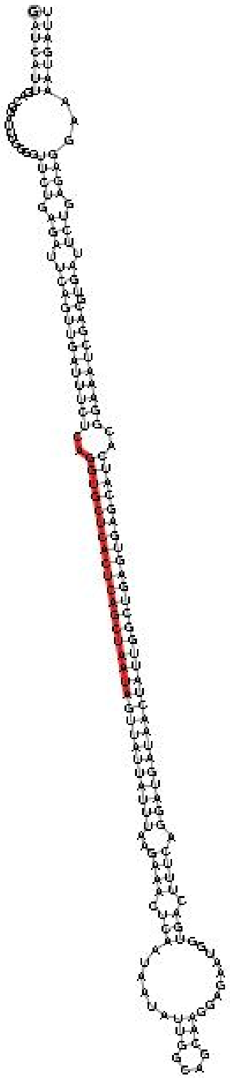

sly-miR9471b-3p_sly-MIR9471b

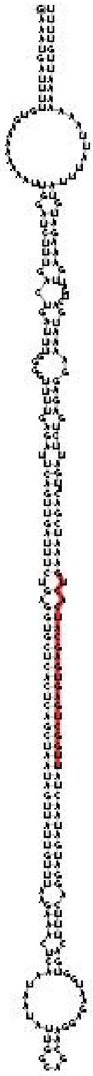

sly-miR9471b-5p_sly-MIR9471b

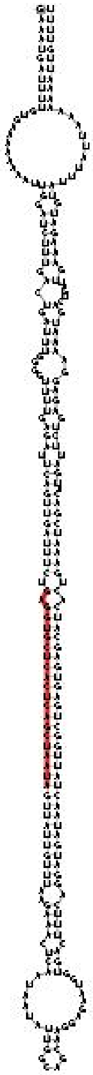

sly-miR9472-3p_sly-MIR9472

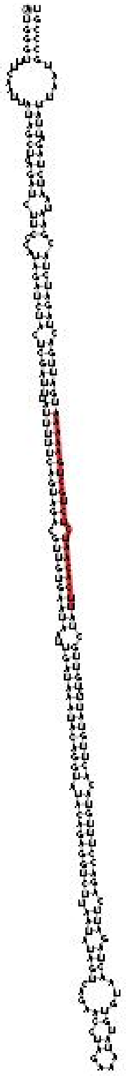

sly-miR9472-5p_sly-MIR9472

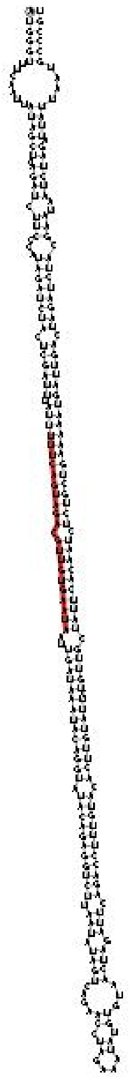

sly-miR9473-5p_sly-MIR9473

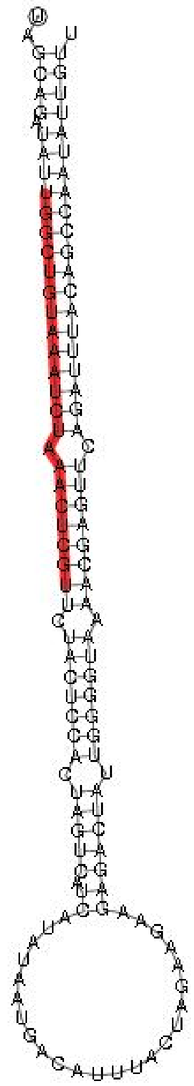

sly-miR9474-3p_sly-MIR9474

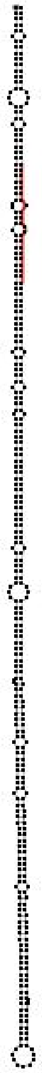

sly-miR9474-5p_sly-MIR9474

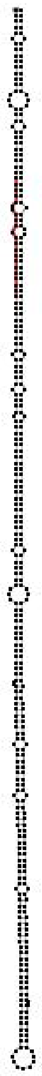

sly-miR9476-3p_sly-MIR9476

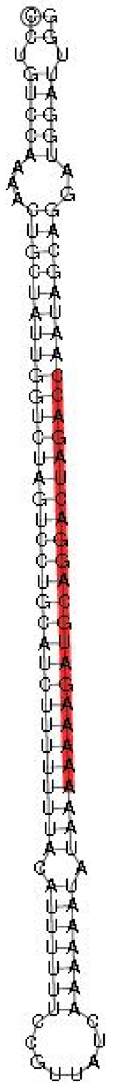

sly-miR9476-5p_sly-MIR9476

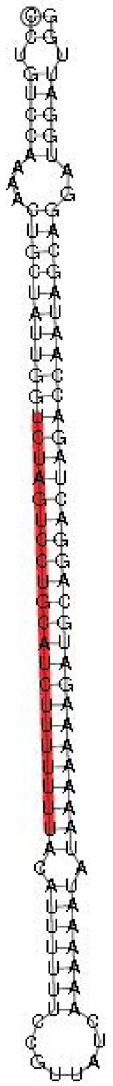

sly-miR9477-3p_sly-MIR9477

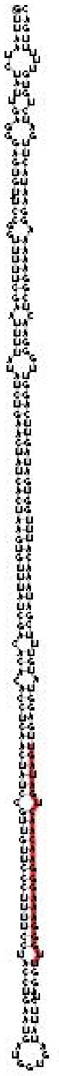

sly-miR9477-5p_sly-MIR9477

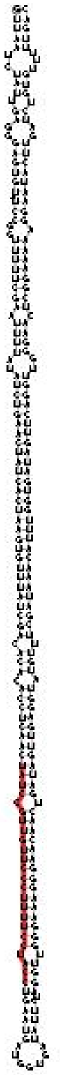

sly-miR9478-3p_sly-MIR9478

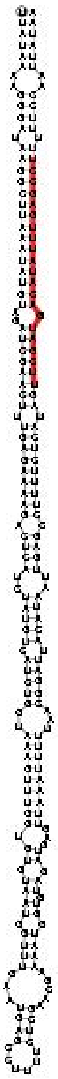

sly-miR9478-5p_sly-MIR9478

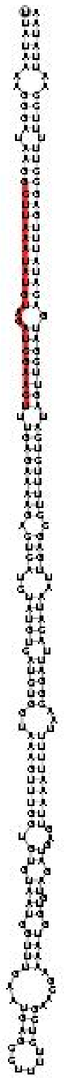

sly-miR9479-3p_sly-MIR9479

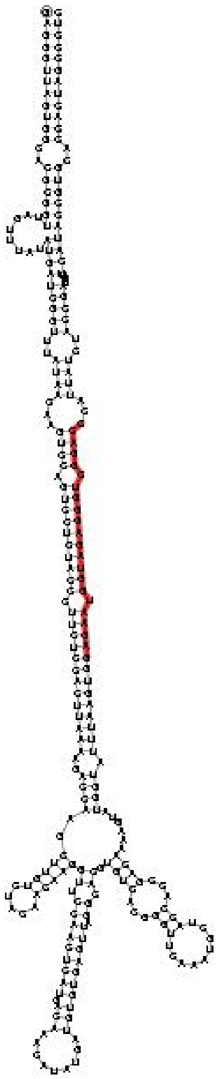

sly-miR9479-5p_sly-MIR9479

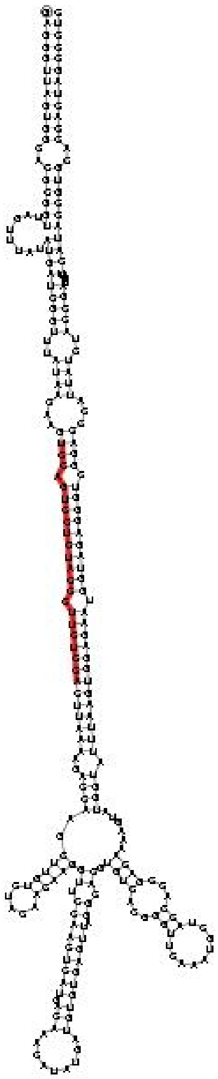

Supplement: S1 Fig — The whole sequences are miRNA precursors, and the red prominent parts are the mature sequences. (PDF) [file pone.0237690.s001.pdf]
