# Supplementary material for: miRNA expression profiling and zeatin dynamic changes in a new model system of in vivo indirect regeneration of tomato
Source: PLoS One. 2020 Dec 17;15(12):e0237690. doi: 10.1371/journal.pone.0237690 (PMC7745965; doi:10.1371/journal.pone.0237690)

novel_1_novel_1

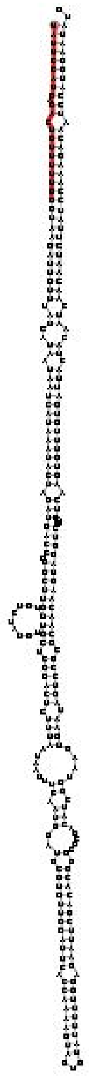

novel_9_novel_9

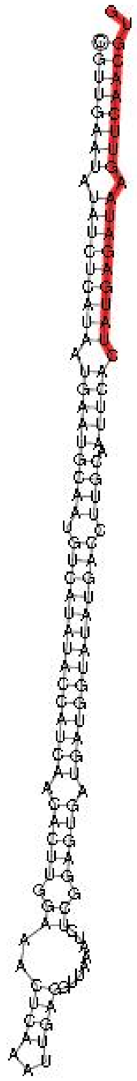

novel_13_novel_13

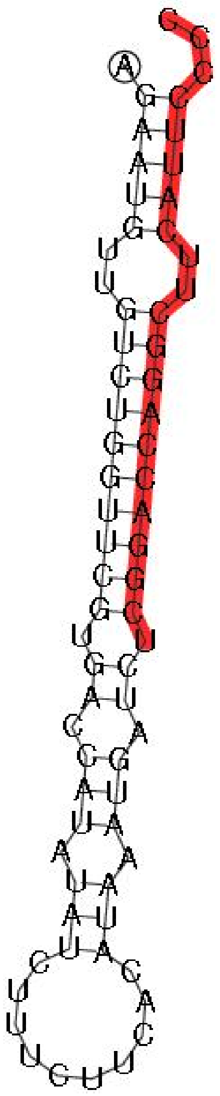

novel_22_novel_22

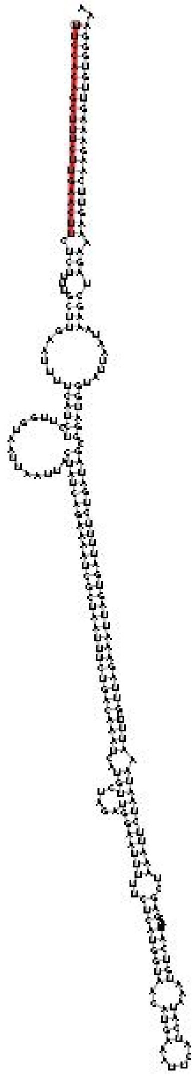

novel_31_novel_31

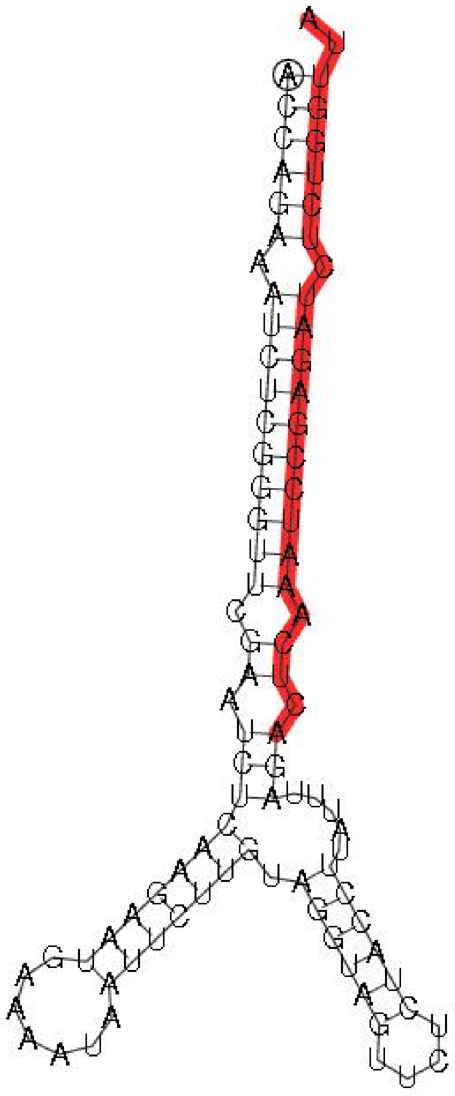

novel_32_novel_32

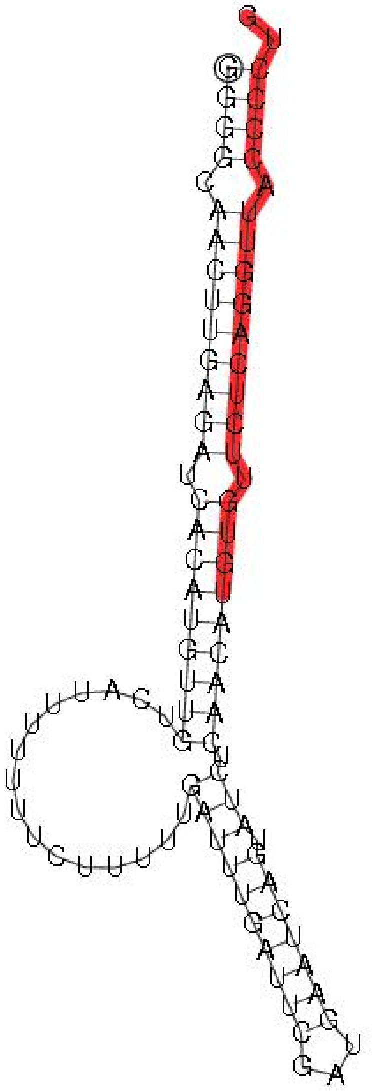

novel_33_novel_33

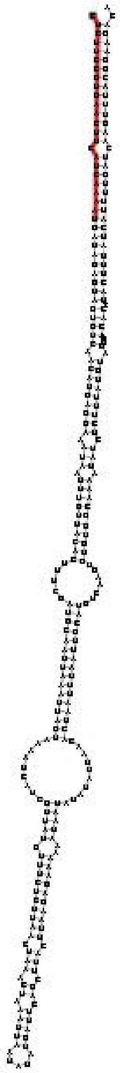

novel_35_novel_35

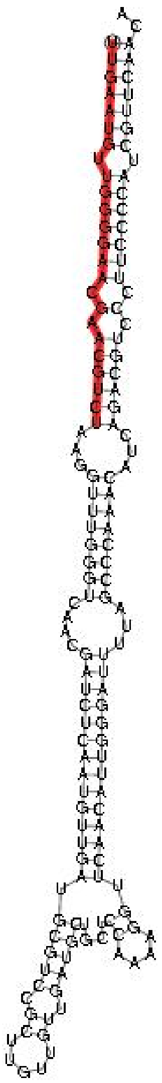

novel_38_novel_38

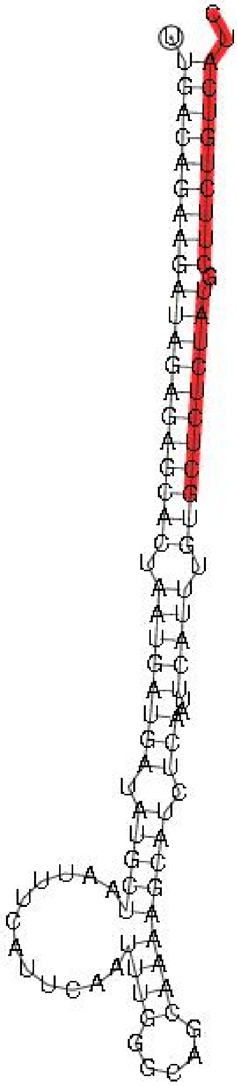

novel_40_novel_40

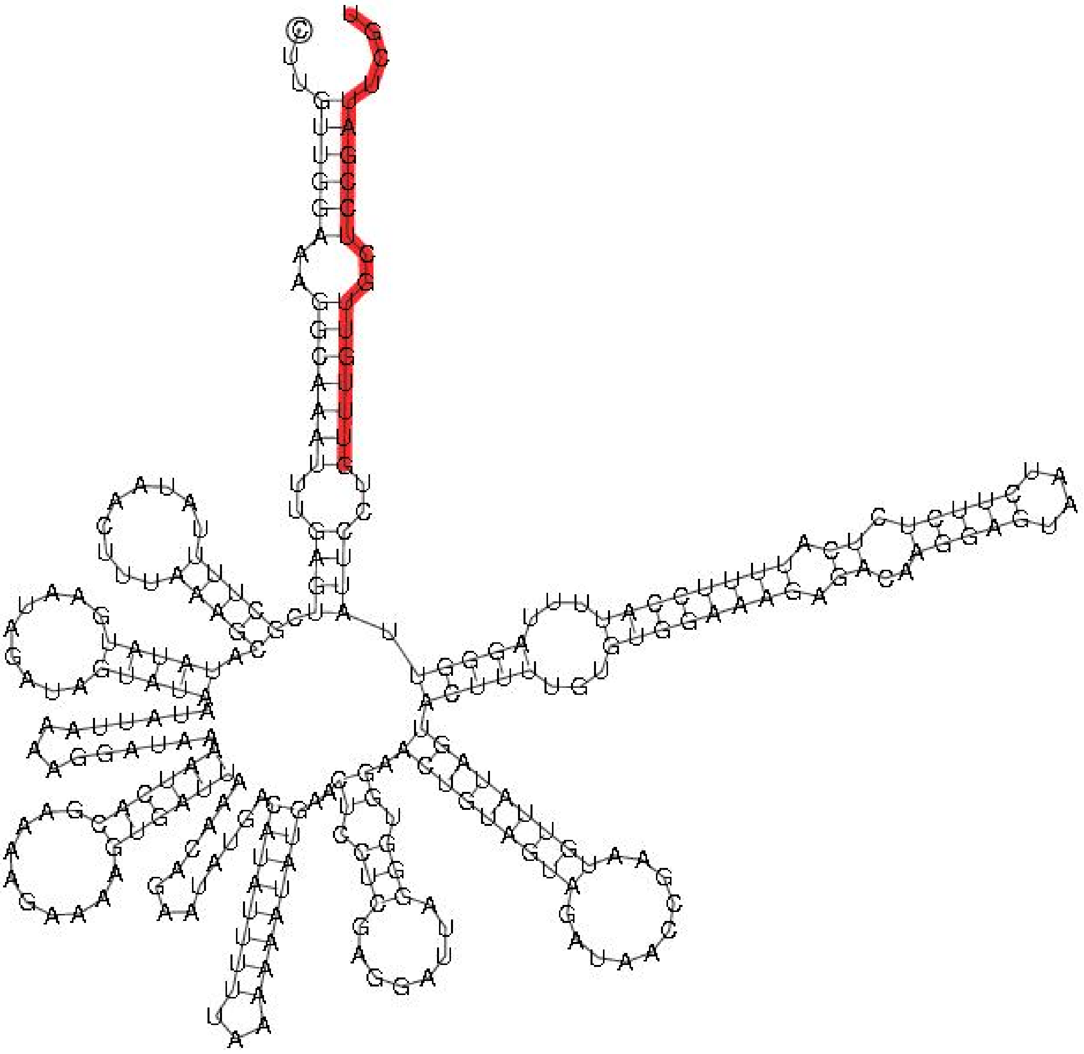

novel_42_novel_42

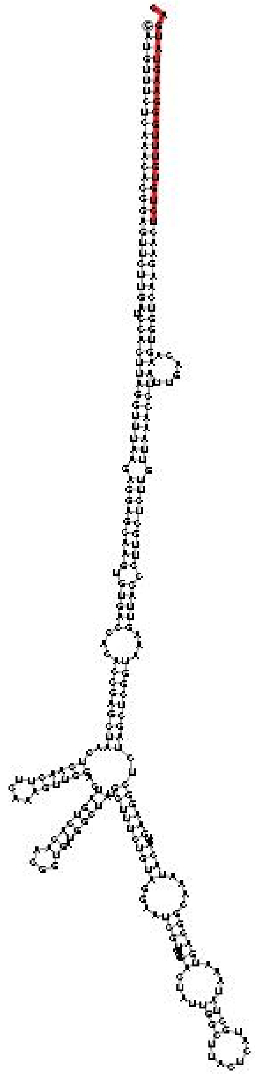

novel_43_novel_43

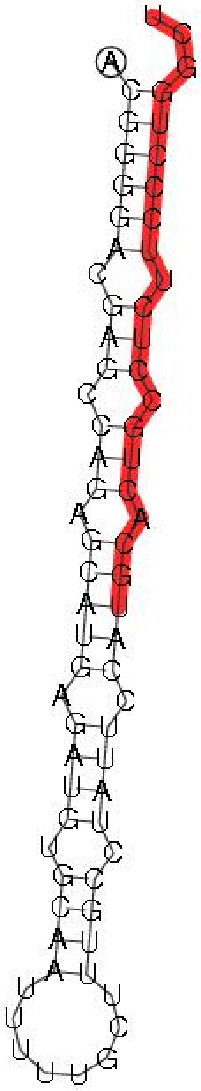

novel_44_novel_44

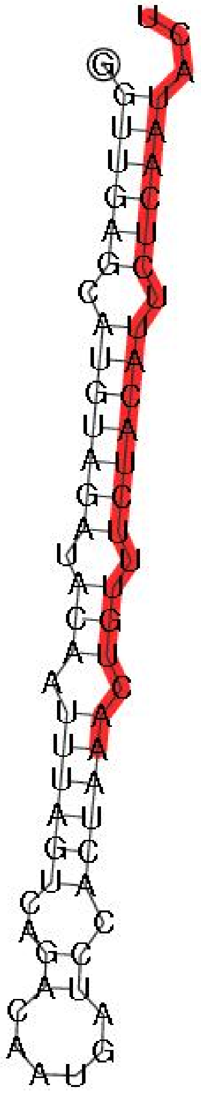

novel_46_novel_46

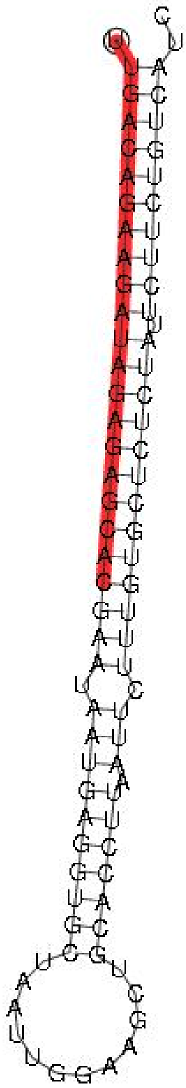

novel_51_novel_51

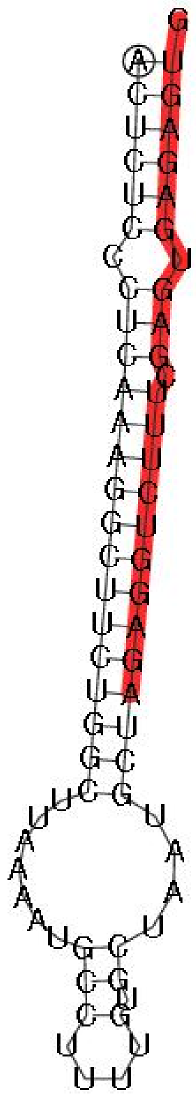

novel_54_novel_54

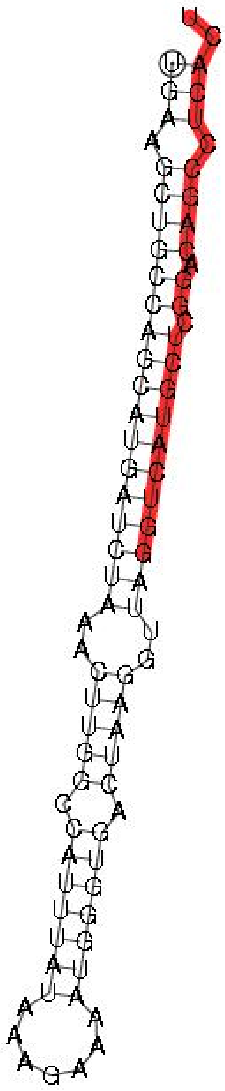

novel_55_novel_55

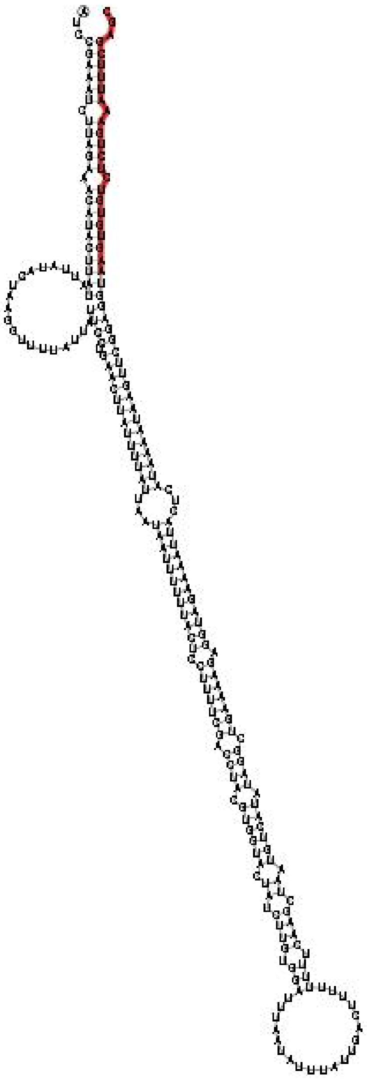

novel_56_novel_56

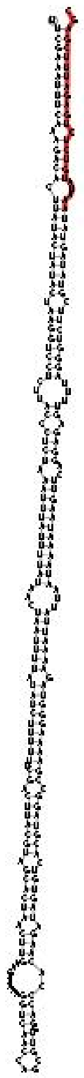

novel_58_novel_58

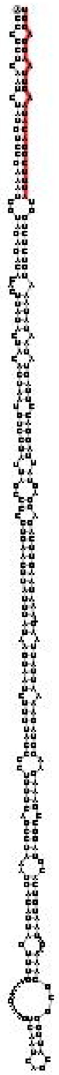

novel_59_novel_59

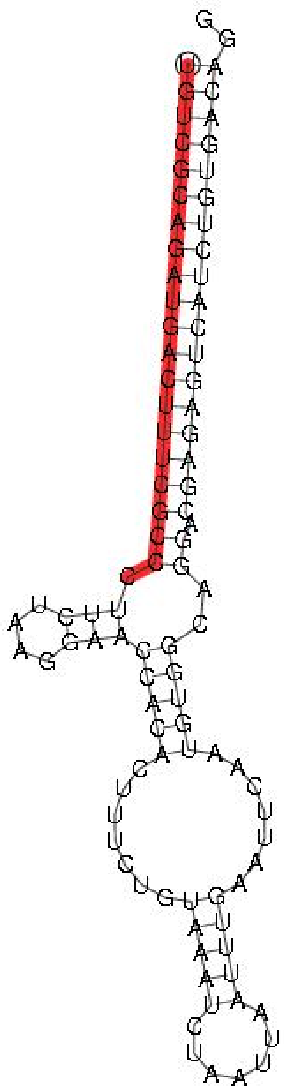

novel_63_novel_63

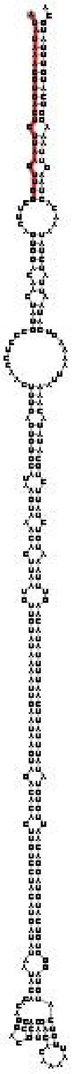

novel_64_novel_64

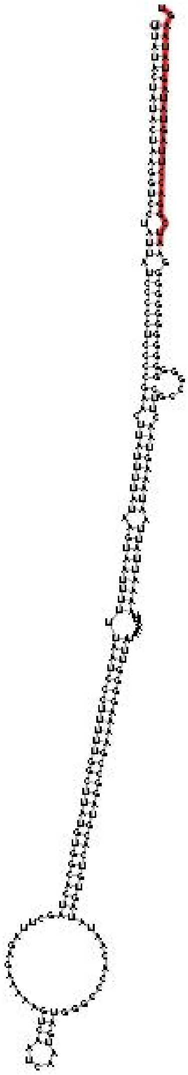

novel_66_novel_66

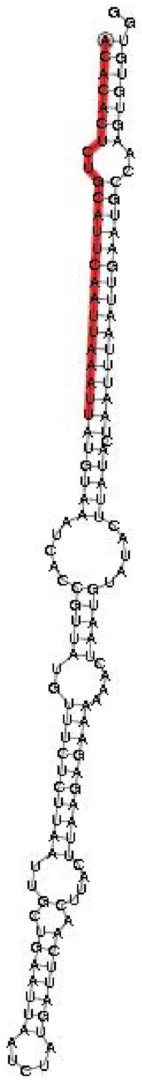

novel_67_novel_67

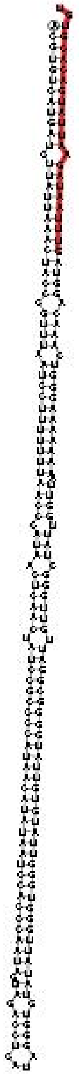

novel_70_novel_70

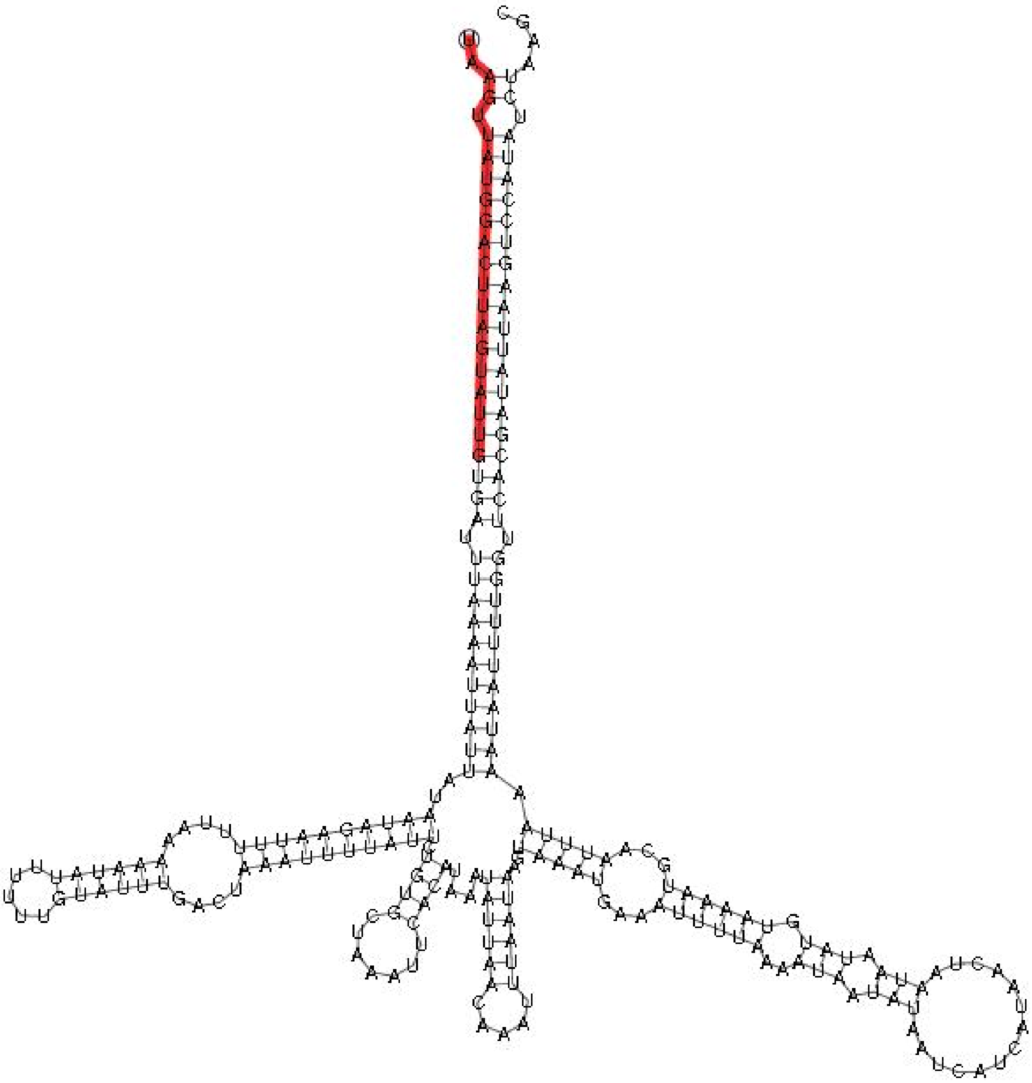

novel_75_novel_75

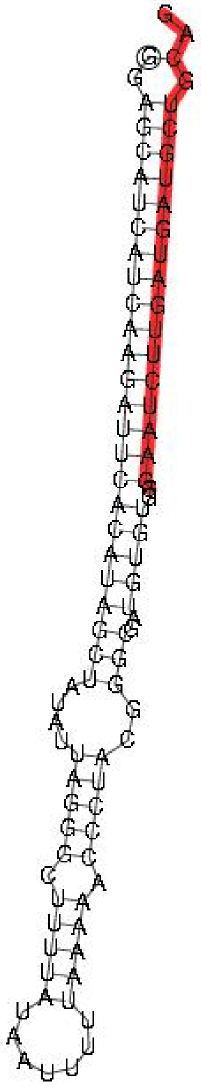

novel_79_novel_79

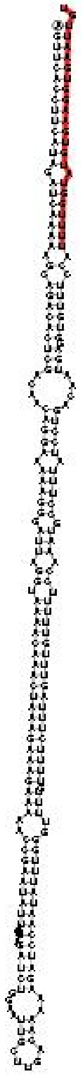

novel_80_novel_80

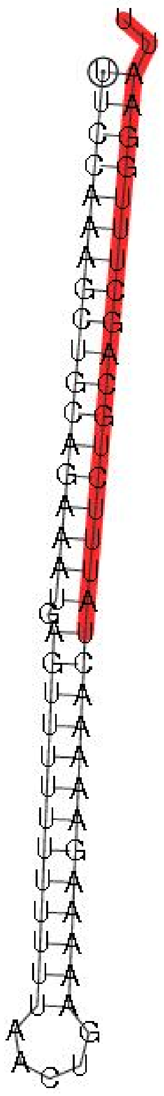

novel_83_novel_83

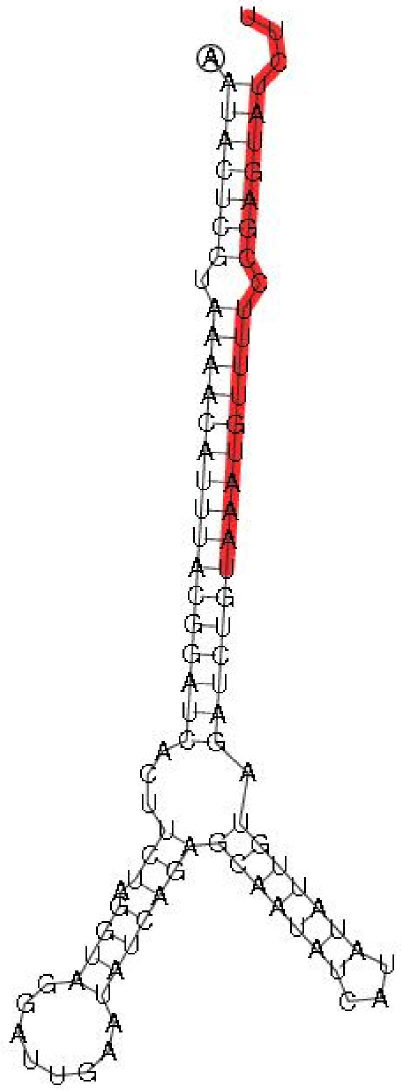

novel_84_novel_84

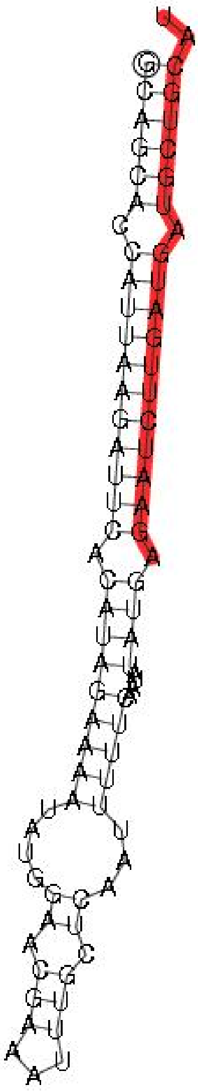

novel_85_novel_85

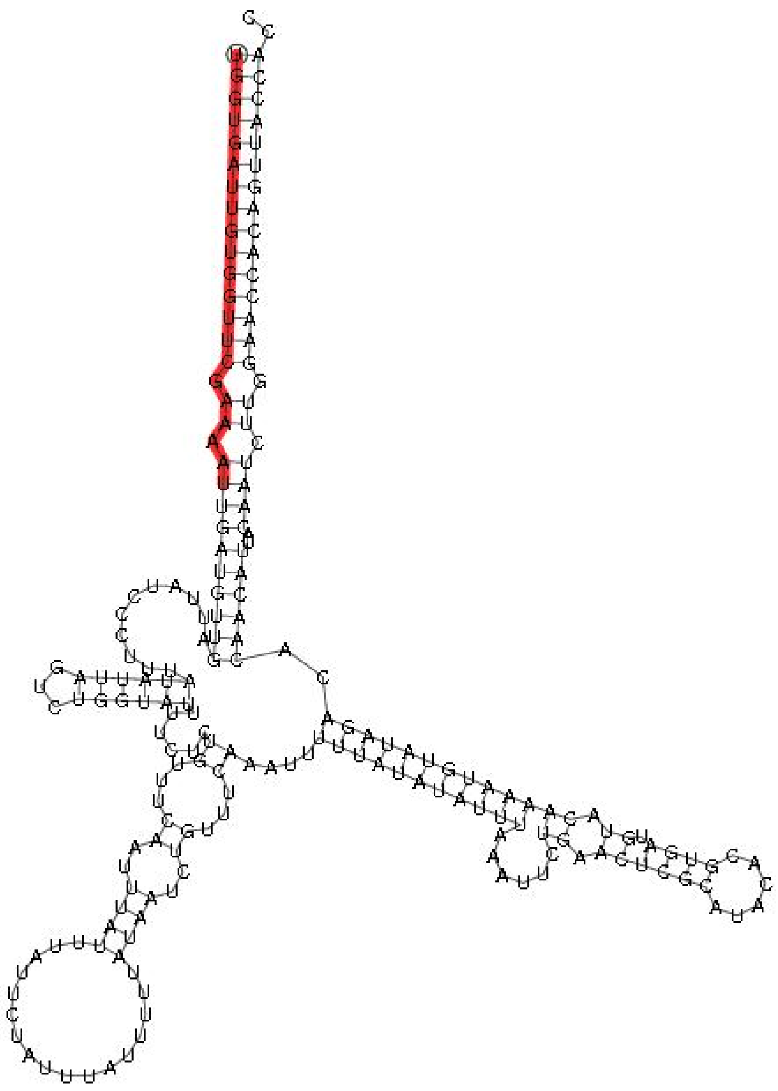

novel_88_novel_88

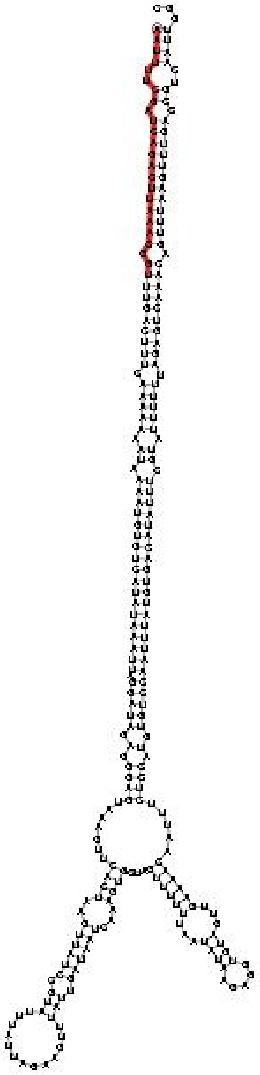

novel_89_novel_89

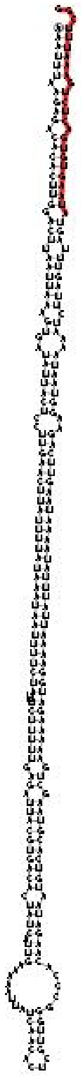

novel_93_novel_93

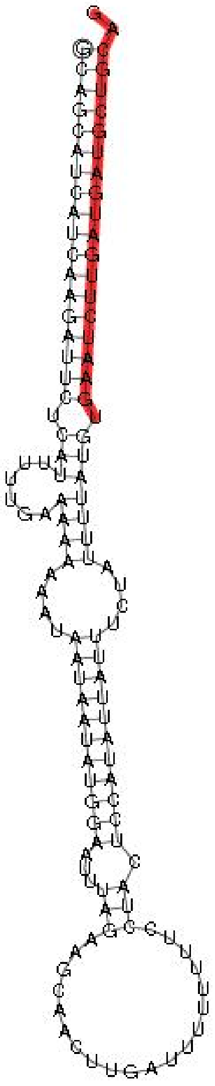

novel_100_novel_100

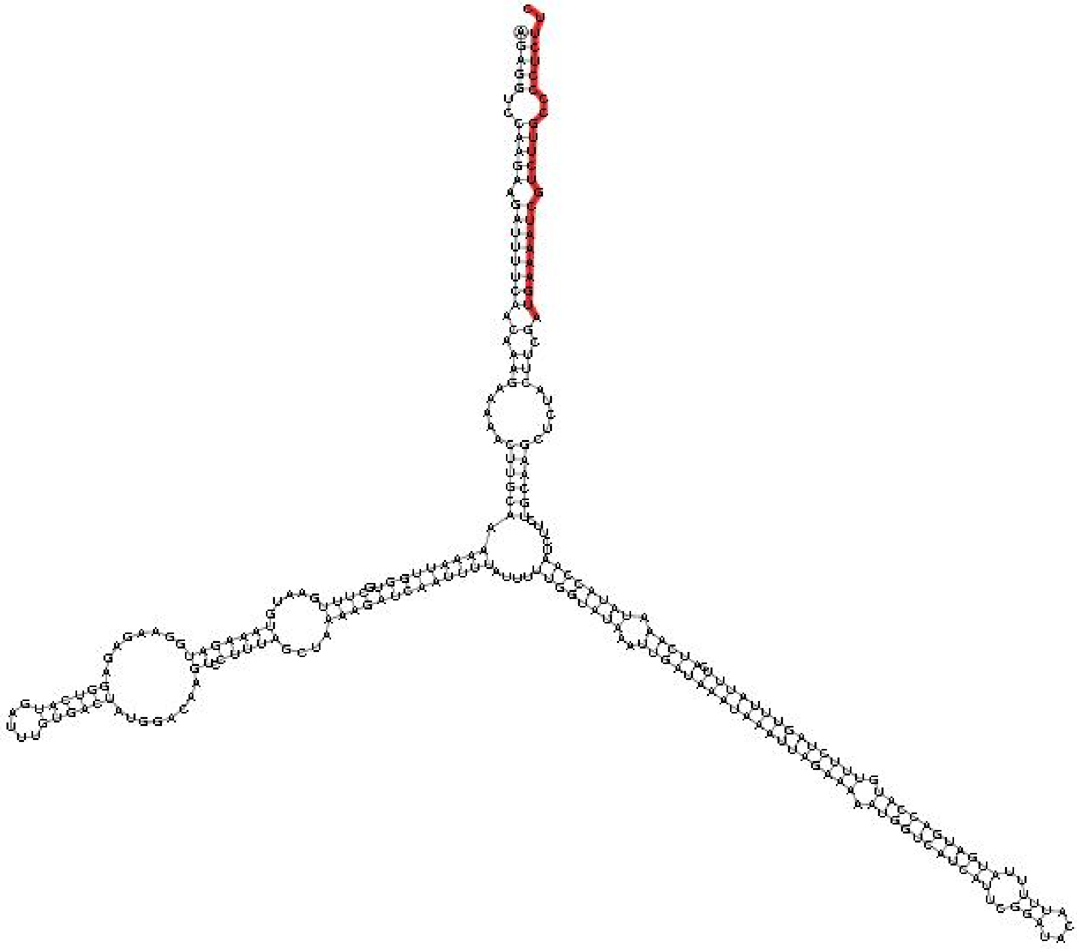

novel_101_novel_101

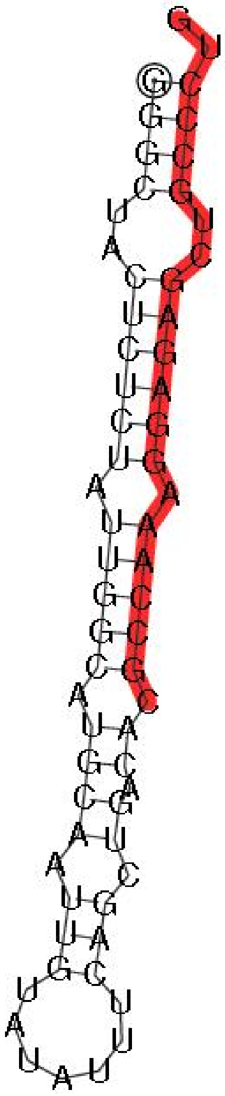

novel_105_novel_105

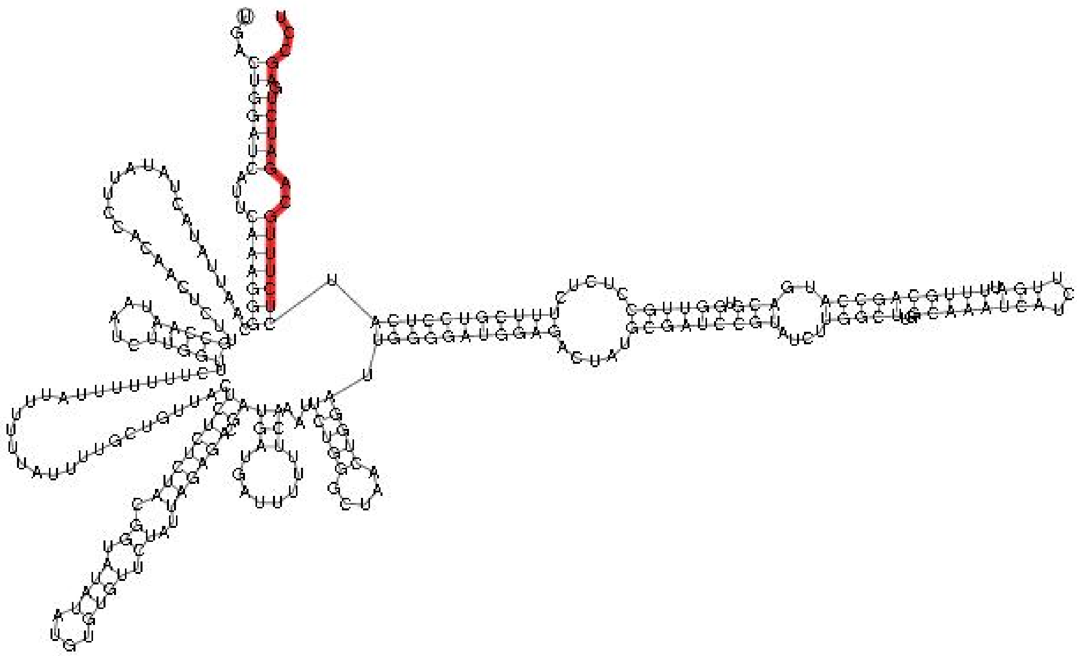

novel_106_novel_106

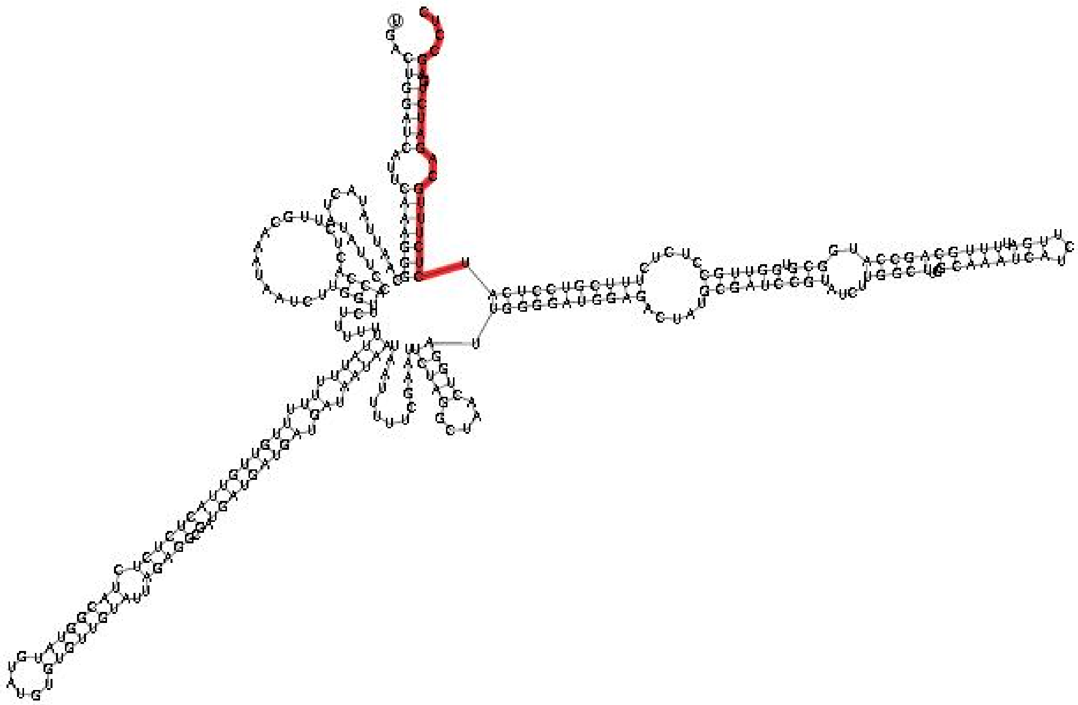

novel_107_novel_107

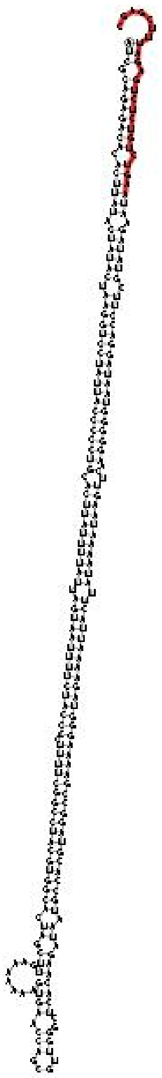

novel_108_novel_108

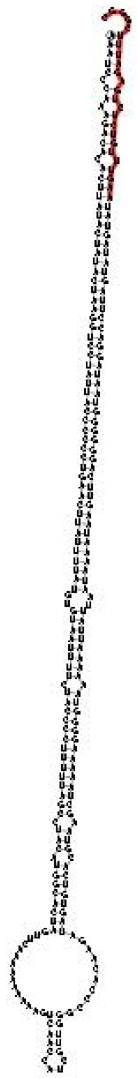

novel_109_novel_109

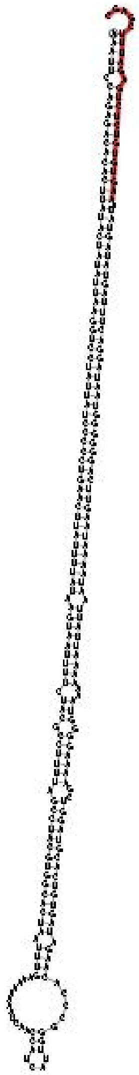

novel_110_novel_110

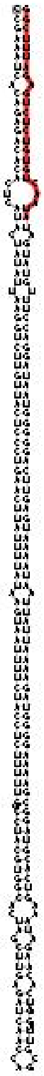

novel_111_novel_111

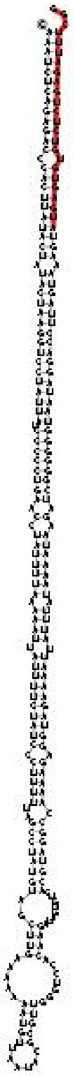

novel_112_novel_112

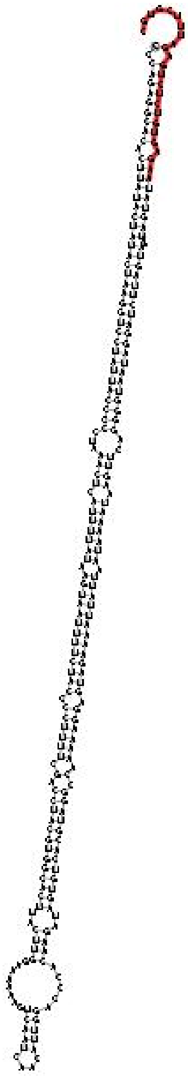

novel_113_novel_113

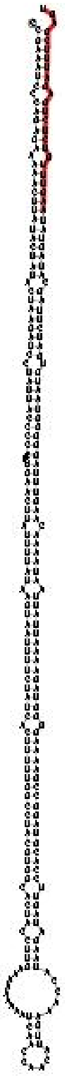

novel_114_novel_114

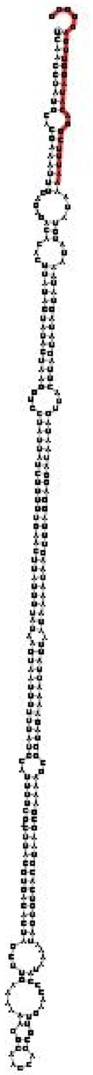

novel_116_novel_116

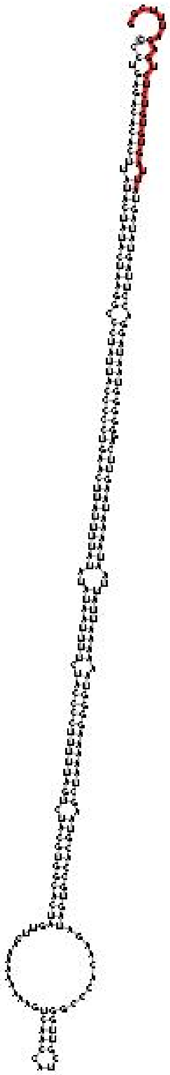

novel_117_novel_117

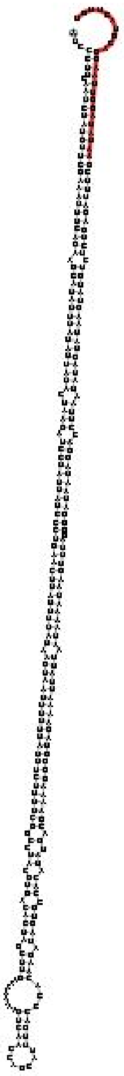

novel_118_novel_118

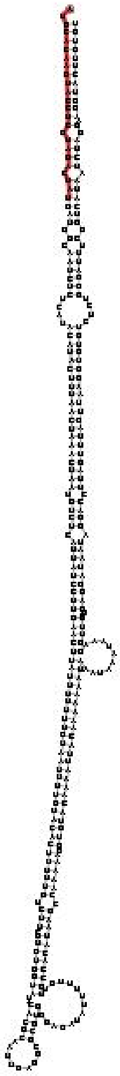

novel_119_novel_119

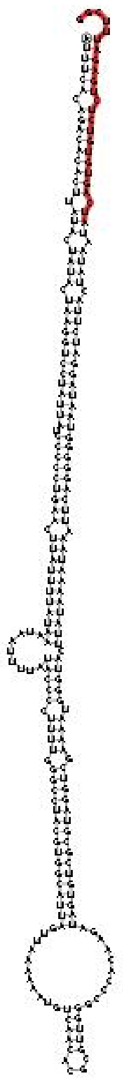

novel_120_novel_120

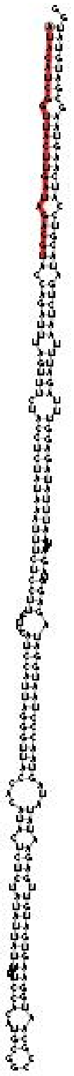

novel_121_novel_121

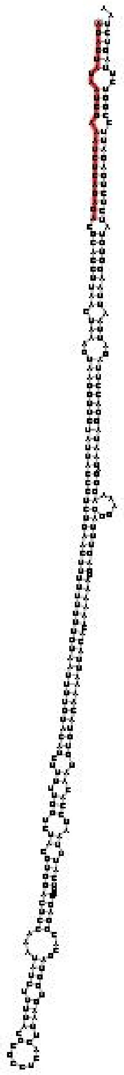

novel_125_novel_125

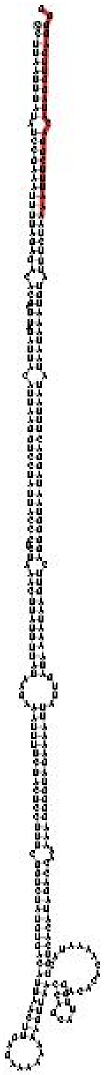

novel_127_novel_127

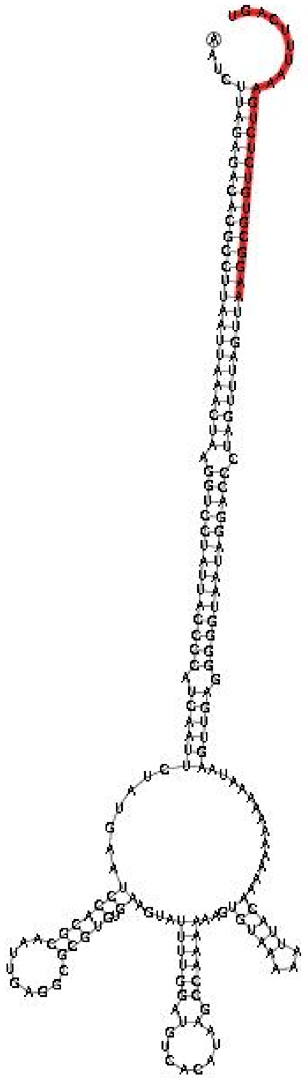

novel_128_novel_128

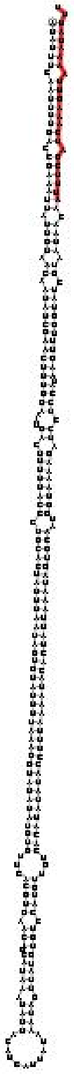

novel_129_novel_129

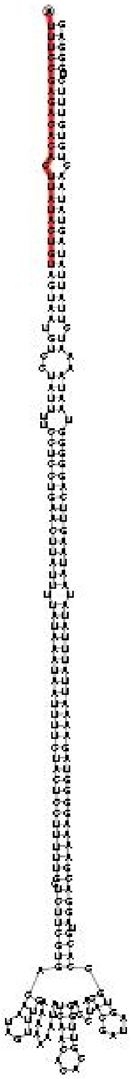

novel_130_novel_130

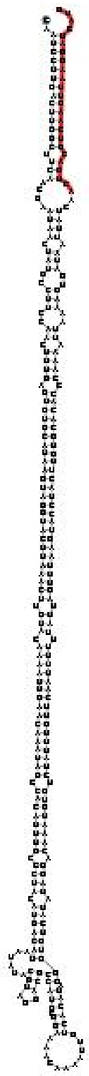

novel_131_novel_131

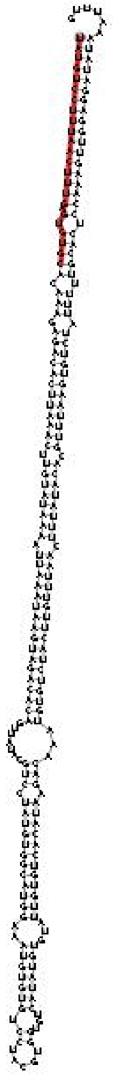

novel_133_novel_133

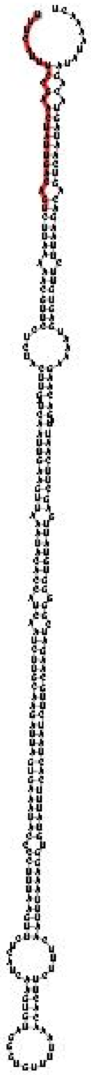

novel_134_novel_134

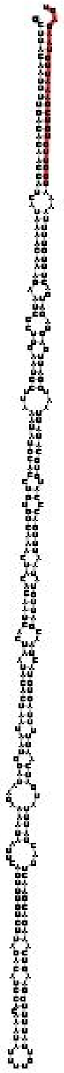

novel_136_novel_136

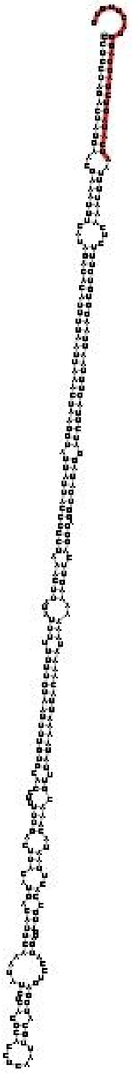

novel_137_novel_137

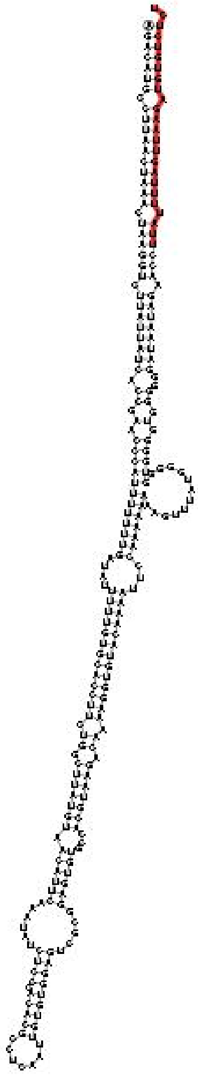

novel_139_novel_139

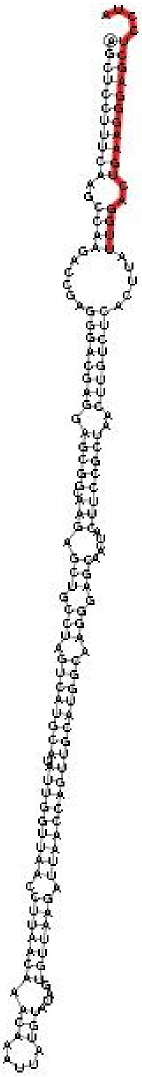

novel_140_novel_140

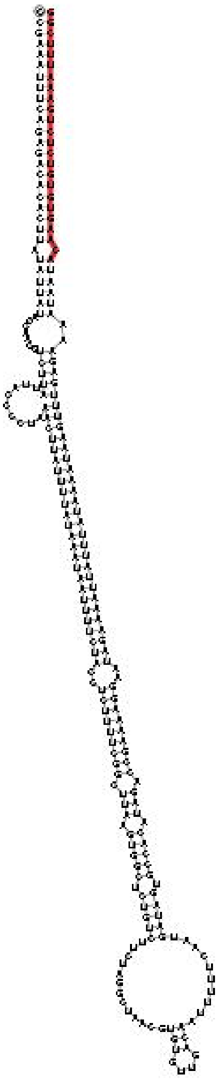

novel_141_novel_141

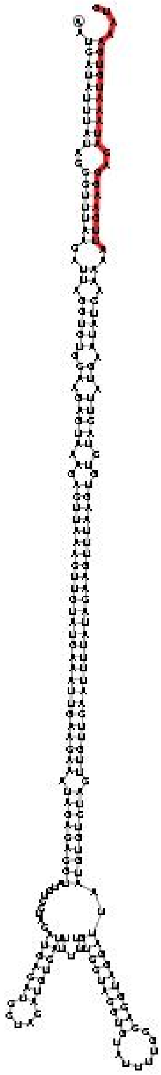

novel_142_novel_142

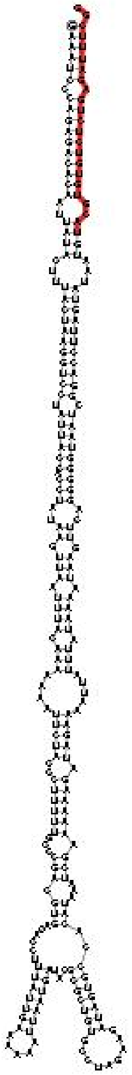

novel_144_novel_144

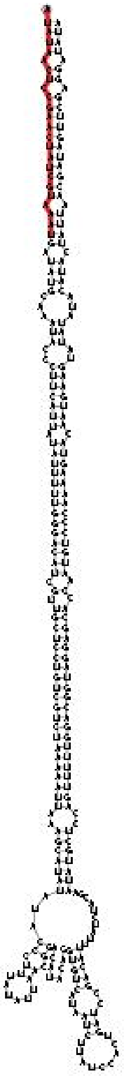

novel_145_novel_145

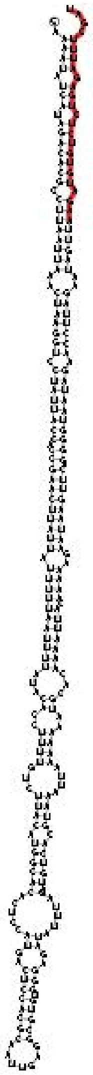

novel_148_novel_148

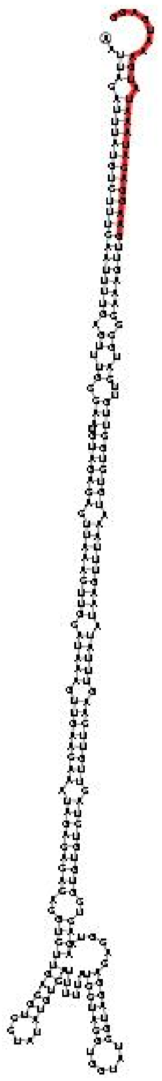

novel_149_novel_149

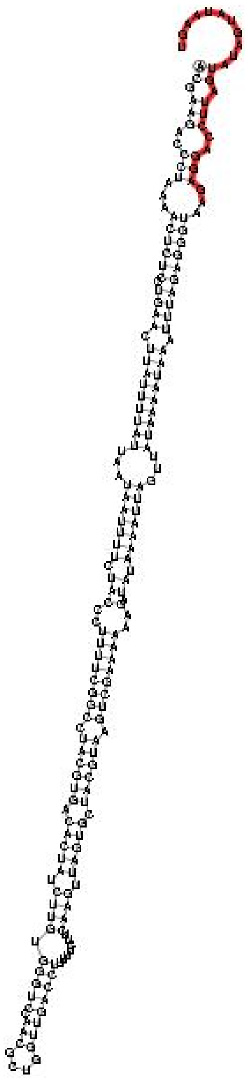

novel_150_novel_150

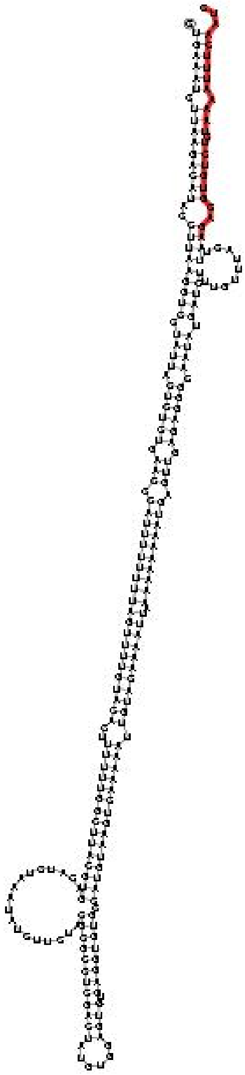

novel_152_novel_152

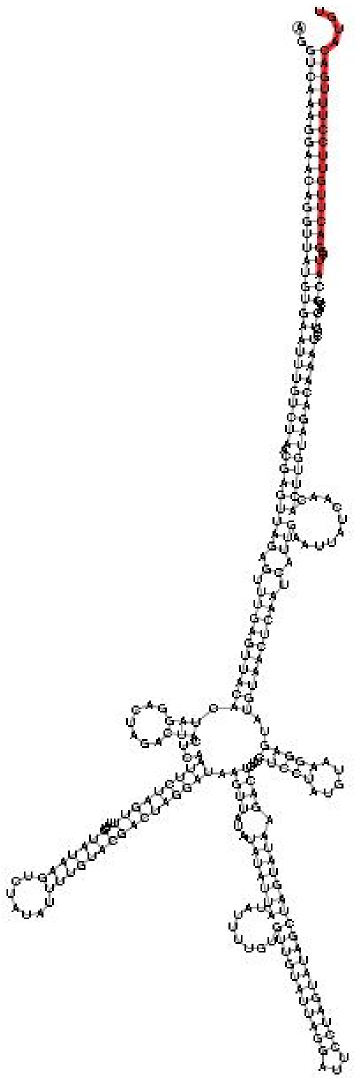

novel_153_novel_153

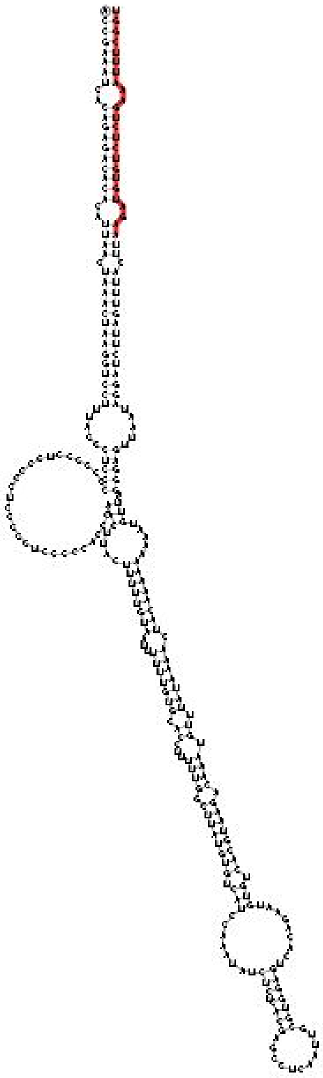

novel_154_novel_154

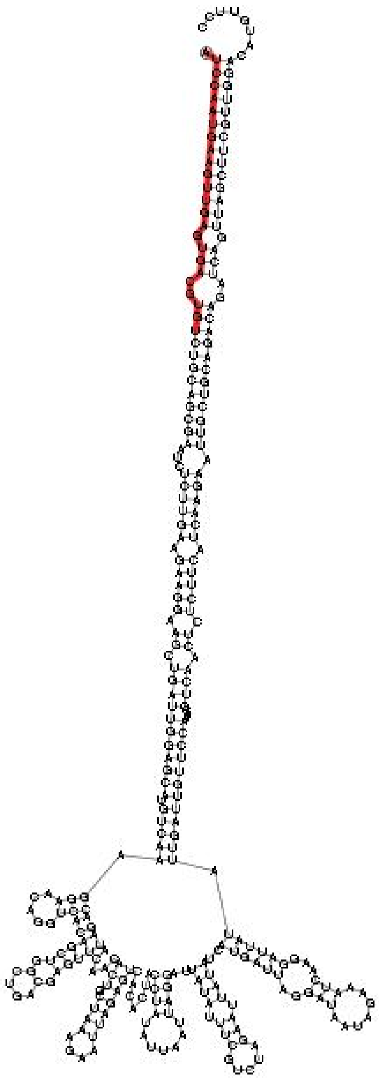

novel_156_novel_156

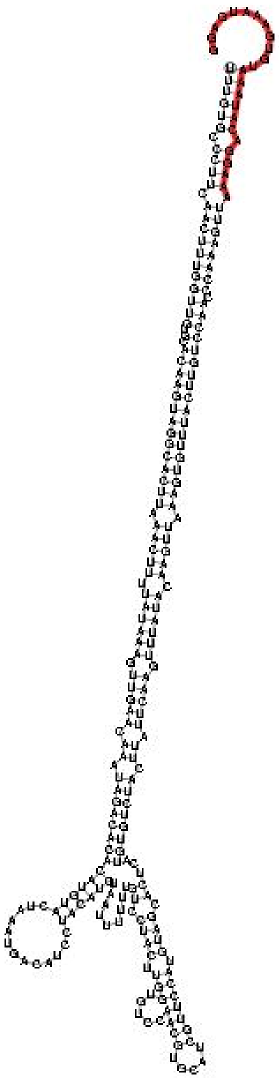

novel_159_novel_159

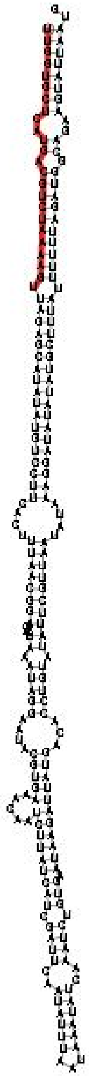

novel_160_novel_160

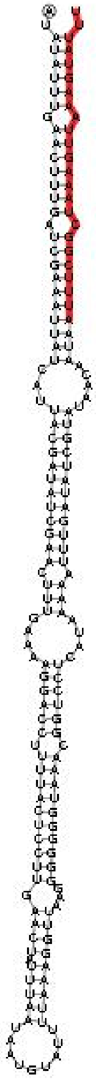

novel_161_novel_161

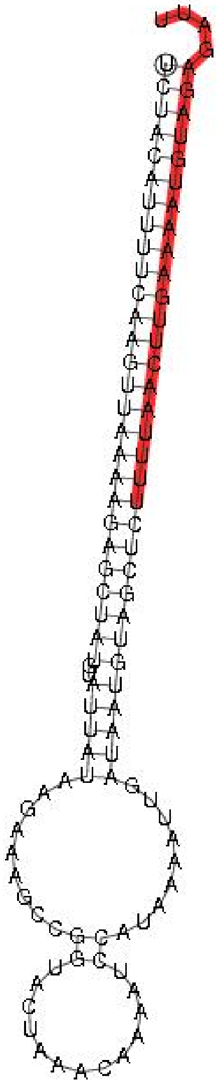

novel_162_novel_162

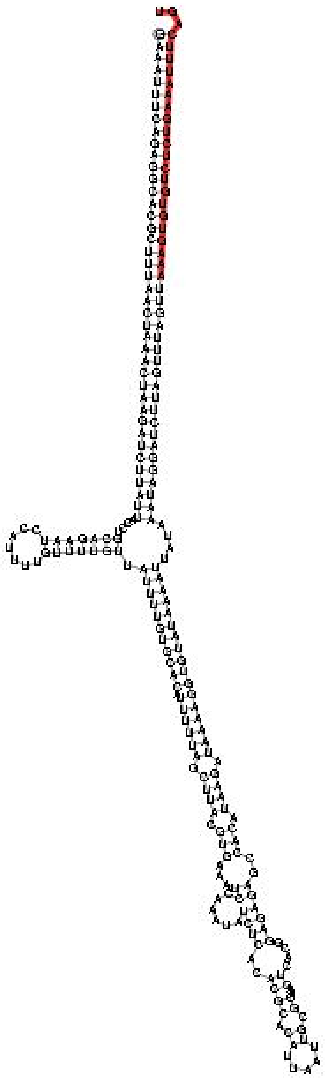

novel_164_novel_164

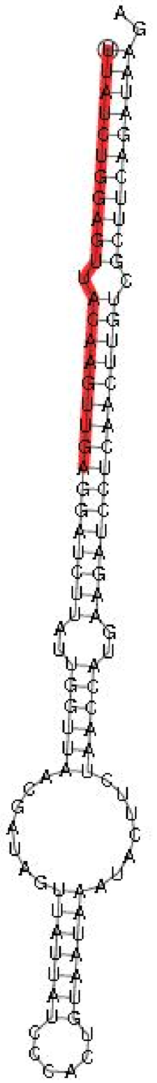

novel_166_novel_166

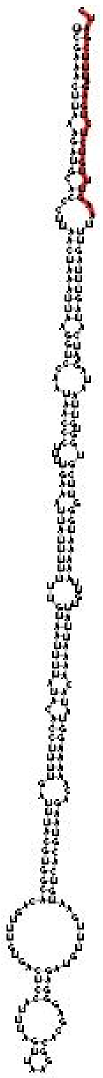

novel_167_novel_167

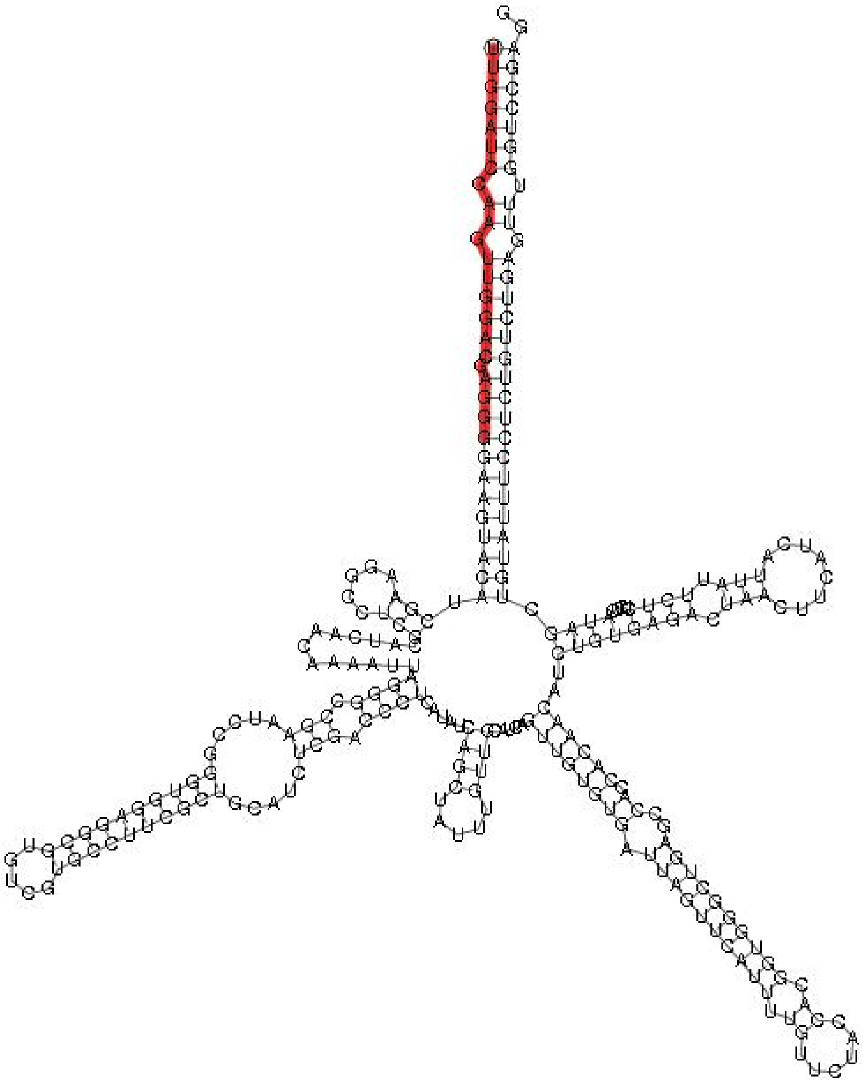

Supplement: S2 Fig — The whole sequences are miRNA precursors, and the red prominent parts are the mature sequences. (PDF) [file pone.0237690.s002.pdf]
