# Supplementary material for: miRNA expression profiling and zeatin dynamic changes in a new model system of in vivo indirect regeneration of tomato
Source: PLoS One. 2020 Dec 17;15(12):e0237690. doi: 10.1371/journal.pone.0237690 (PMC7745965; doi:10.1371/journal.pone.0237690)
Supplement: S1 Table — (DOCX) [file pone.0237690.s003.docx]

**Table S1 | Primers used in this study for qRT-PCR.**

| MiRNA | Primer(Forward) |
| --- | --- |
| Novel 128 | ACACTCCAGCTGGGATTTCGATCA |
| Novel 56 | ACACTCCAGCTGGGAAATATGTCT |
| Sly-miR166c | TAGATGGATCAGAAGGAGTAGC |
| Sly-miR167a | ACATCGTGCAGCACTAGCAG |
| Sly-miR396 | CGTTCCACAGCTTTCTTGAACTG |
| Sly-miR397 | GATTGAGTGCAGCGTTGATGA |
