# Supplementary material for: miRNA expression profiling and zeatin dynamic changes in a new model system of in vivo indirect regeneration of tomato
Source: PLoS One. 2020 Dec 17;15(12):e0237690. doi: 10.1371/journal.pone.0237690 (PMC7745965; doi:10.1371/journal.pone.0237690)
Supplement: S2 Table — a Trans-zeatin weight, calculated by the standard curve y = 0.0651x–3.3111, R2 = 0.9963 of HPLC. b Trans-zeatin content, obtained by dividing the weight by the fresh weight of the samples. (DOCX) [file pone.0237690.s004.docx]

**Table S2 | The content of trans-zeatin during *in vivo* regeneration of tomato micro-TOM.**

| **Days after decapitation**  **(d)** | **Fresh weight (g)** | **Trans-zeatin weight^a^ (μg)** | | | | **Trans-zeatin content^b^**  **(μg/g)** |
| --- | --- | --- | --- | --- | --- | --- |
|  |  | **Repeat 1** | **Repeat 2** | **Repeat 3** | **Average** |  |
| 0 | 1.5660 | 0 | 0 | 0 | 0 | 0 |
| 9 | 0.8183 | 10.9247 | 10.9298 | 10.9265 | 10.9270 | 13.3534 |
| 12 | 1.0367 | 7.7299 | 7.7354 | 7.7301 | 7.7318 | 7.4581 |
| 15 | 1.3041 | 20.0043 | 19.9825 | 19.9865 | 19.9911 | 15.3294 |
| 18 | 1.0415 | 9.1583 | 9.1581 | 9.1624 | 9.1596 | 8.7946 |
| 21 | 1.5484 | 24.2317 | 24.2360 | 24.2379 | 24.2352 | 15.6518 |
| 24 | 1.9145 | 96.6112 | 96.6042 | 96.6131 | 96.6095 | 50.4620 |
| 30 | 1.5600 | 72.1669 | 72.1683 | 72.1673 | 72.1675 | 46.2612 |

^a^ Trans-zeatin weight, calculated by the standard curve y=0.0651x–3.3111, R^2^=0.9963 of HPLC.

^b^ Trans-zeatin content, obtained by dividing the weight by the fresh weight of the samples.
