# Supplementary material for: miRNA expression profiling and zeatin dynamic changes in a new model system of in vivo indirect regeneration of tomato
Source: PLoS One. 2020 Dec 17;15(12):e0237690. doi: 10.1371/journal.pone.0237690 (PMC7745965; doi:10.1371/journal.pone.0237690)
Supplement: S3 Table — The samples were the cutting surface of stem at 0, 30, 37, 44, 51 and 58 d after decapitation, 10 tomato plants were contained in each sample. (DOCX) [file pone.0237690.s005.docx]

**Table S3 | The number of regenerated adventitious shoots in lovastatin and control treated tomato plants.**

| **Days after decapitation(d)** | **0** | **30** | **37** | **44** | **51** | **58** |
| --- | --- | --- | --- | --- | --- | --- |
| Lovastatin | 0 | 1.1 | 6.3 | 12.6 | 15.5 | 21.3 |
| Control | 0 | 1.1 | 7.1 | 11.7 | 15.9 | 20.7 |

The samples were the cutting surface of stem at 0, 30, 37, 44, 51 and 58 d after decapitation, 10 tomato plants were contained in each sample.
