# Supplementary material for: miRNA expression profiling and zeatin dynamic changes in a new model system of in vivo indirect regeneration of tomato
Source: PLoS One. 2020 Dec 17;15(12):e0237690. doi: 10.1371/journal.pone.0237690 (PMC7745965; doi:10.1371/journal.pone.0237690)
Supplement: S4 Table — a Q20, The percentage of bases with Phred value greater than 20 in the total bases. b Q30, The percentage of bases with Phred value greater than 30 in the total bases. (DOCX) [file pone.0237690.s006.docx]

**Table S4 | Quality of raw reads of two sRNA libraries produced from stem and callus.**

| **Sample** | **Raw reads** | **Bases** | **Error rate** | **Q20^a^** | **Q30^b^** | **GC content** |
| --- | --- | --- | --- | --- | --- | --- |
| Stem | 13563211 | 0.678G | 0.02% | 95.59% | 90.79% | 47.99% |
| Callus | 10988386 | 0.549G | 0.01% | 96.74% | 90.61% | 48.06% |

^a^ Q20, The percentage of bases with Phred value greater than 20 in the total bases.

^b^ Q30, The percentage of bases with Phred value greater than 30 in the total bases
