# Supplementary material for: miRNA expression profiling and zeatin dynamic changes in a new model system of in vivo indirect regeneration of tomato
Source: PLoS One. 2020 Dec 17;15(12):e0237690. doi: 10.1371/journal.pone.0237690 (PMC7745965; doi:10.1371/journal.pone.0237690)
Supplement: S5 Table — (DOCX) [file pone.0237690.s007.docx]

**Table S5 | Detailed information of known miRNAs in two small RNA libraries derived from stem and callus.**

| **miRNA** | **Sequence** | **Number of reads** | |
| --- | --- | --- | --- |
|  |  | **Stem** | **Callus** |
| Sly-miR156a | UUGACAGAAGAUAGAGAGCAC | 1321 | 1565 |
| Sly-miR156d-3p | UGACAGAAGAGAGUGAGCAC | 4 | 11 |
| Sly-miR156d-5p | GCUCACUGCUCUAUCUGUCACC | 127 | 258 |
| Sly-miR156e-3p | UGAUAGAAGAGAGUGAGCAC | 9 | 1 |
| Sly-miR156e-5p | GCUUACUCUCUAUCUGUCACC | 95 | 41 |
| Sly-miR159 | UUUGGAUUGAAGGGAGCUCUA | 45041 | 43407 |
| Sly-miR160a | UGCCUGGCUCCCUGUAUGCCA | 71 | 15 |
| Sly-miR162 | UCGAUAAACCUCUGCAUCCAG | 4728 | 4361 |
| Sly-miR164a-3p | CAUGUGCCUGUUUUCCCCAUC | 6 | 21 |
| Sly-miR164a-5p | UGGAGAAGCAGGGCACGUGCA | 153 | 343 |
| Sly-miR164b-3p | CACGUGUUCUCCUUCUCCAAC | 1 | 15 |
| Sly-miR166a | UCGGACCAGGCUUCAUUCCCC | 4211 | 3380 |
| Sly-miR166c-3p | GGGAUGUUGUCUGGCUCGACA | 4967 | 1737 |
| Sly-miR166c-5p | UCGGACCAGGCUUCAUUCCUC | 468 | 120 |
| Sly-miR167a | UGAAGCUGCCAGCAUGAUCUA | 11 | 194 |
| Sly-miR167b-3p | UAAAGCUGCCAGCAUGAUCUGG | 4 | 5 |
| Sly-miR167b-5p | AGGUCAUCUAGCAGCUUCAAU | 19 | 32 |
| Sly-miR168a-3p | UCGCUUGGUGCAGGUCGGGAC | 344 | 307 |
| Sly-miR168a-5p | CCUGCCUUGCAUCAACUGAAU | 651 | 268 |
| Sly-miR168b-3p | CCCGCCUUGCAUCAACUGAAU | 241 | 360 |
| Sly-miR169a | CAGCCAAGGAUGACUUGCCGG | 0 | 1 |
| Sly-miR169e-3p | UGGCAAGCAUCUUUGGCGACU | 143 | 20 |
| Sly-miR171a | UGAUUGAGCCGUGCCAAUAUC | 155 | 13 |
| Sly-miR171b | UUGAGCCGUGCCAAUAUCACG | 24 | 7 |
| Sly-miR171c | UAUUGGUGCGGUUCAAUGAGA | 4 | 6 |
| Sly-miR171d | UUGAGCCGCGCCAAUAUCAC | 21 | 3 |
| Sly-miR171e | UUGAGCCGCGUCAAUAUCUCU | 152 | 60 |
| Sly-miR172a | AGAAUCUUGAUGAUGCUGCAU | 189 | 84 |
| Sly-miR1916 | AUUUCACUUAGACACCUCAA | 5 | 3 |
| Sly-miR1917 | AUUAAUAAAGAGUGCUAAAGU | 14 | 14 |
| Sly-miR1918 | UGUUGGUGAGAGUUCGAUUCUC | 4 | 5 |
| Sly-miR1919a | ACGAGAGUCAUCUGUGACAGG | 106 | 62 |
| Sly-miR1919c-5p | UGUCGCAGAUGACUUUCGCCC | 185 | 150 |
| Sly-miR319a | CUUGGACUGAAGGGAGCUCC | 960 | 332 |
| Sly-miR319b | UUGGACUGAAGGGAGCUCCCU | 29473 | 12142 |
| Sly-miR319c-3p | UUGGACUGAAGGGAGCUCCUU | 15141 | 5625 |
| Sly-miR319c-5p | AGAGCUUCCUUCAGCCCACUC | 13 | 5 |
| Sly-miR390a-3p | AAGCUCAGGAGGGAUAGCACC | 15 | 29 |
| Sly-miR390a-5p | CGCUAUCCAUCCUGAGUUUUA | 80 | 69 |
| Sly-miR390b-3p | CGCUAUCCAUCCUGAGUUUCA | 7 | 13 |
| Sly-miR390b-5p | AAGCUCAGGAGGGAUAGCGCC | 19 | 20 |
| Sly-miR394-3p | AGGUGGGCAUACUGUCAACA | 10 | 3 |
| Sly-miR394-5p | UUGGCAUUCUGUCCACCUCC | 990 | 258 |
| Sly-miR395a | CUGAAGUGUUUGGGGGAACUCC | 30 | 35 |
| Sly-miR396a-3p | UUCCACAGCUUUCUUGAACUG | 862 | 44 |
| Sly-miR396a-5p | GUUCAAUAAAGCUGUGGGAAG | 1862 | 470 |
| Sly-miR396b | UUCCACAGCUUUCUUGAACUU | 7285 | 3305 |
| Sly-miR397 | AUUGAGUGCAGCGUUGAUGA | 414 | 51 |
| Sly-miR403-3p | CUAGAUUCACGCACAAGCUCG | 225 | 162 |
| Sly-miR403-5p | CGUUUGUGCGUGAAUCUAACA | 107 | 54 |
| Sly-miR4376 | ACGCAGGAGAGAUGAUGCUGGA | 909 | 2944 |
| Sly-miR477-3p | UGUCUCUCCCUCAAGGGCUCC | 279 | 104 |
| Sly-miR477-5p | AGUUCUUGUAGGGUGAGACAAC | 25 | 20 |
| Sly-miR482a | UUUCCAAUUCCACCCAUUCCUA | 1739 | 1407 |
| Sly-miR482b | UCUUGCCUACACCGCCCAUGCC | 491 | 537 |
| Sly-miR482c | UCUUGCCAAUACCGCCCAUUCC | 107 | 75 |
| Sly-miR482d-3p | GGAGUGGGUGGGAUGGAAAAA | 65 | 69 |
| Sly-miR482d-5p | UUUCCUAUUCCACCCAUGCCAA | 6 | 11 |
| Sly-miR482e-3p | UCUUUCCUACUCCUCCCAUACC | 2013 | 1808 |
| Sly-miR482e-5p | UGUGGGUGGGGUGGAAAGAUU | 341 | 708 |
| Sly-miR5300 | UCCCCAGUCCAGGCAUUCCAAC | 252 | 216 |
| Sly-miR5302b-5p | UGAAAUGCUAUAGUUGGAAAGU | 13 | 8 |
| Sly-miR5304 | UCAAUGCUACAUACUCAUCCC | 5 | 10 |
| Sly-miR6022 | UGGAAGGGAGAAUAUCCAGGA | 16423 | 16279 |
| Sly-miR6023 | UUCCAUGAAAGAGUUUUUGGAU | 8 | 5 |
| Sly-miR6024 | UUUUAGCAAGAGUUGUUUUACC | 311 | 321 |
| Sly-miR6026 | UUCUUGGCUAGAGUUGUAUUGC | 343 | 60 |
| Sly-miR6027-3p | AUGGGUAGCACAAGGAUUAAUG | 321 | 205 |
| Sly-miR6027-5p | UGAAUCCUUCGGCUAUCCAUAA | 735 | 1582 |
| Sly-miR9469-3p | AUUCGGUCUUCUUAUGUGGAC | 0 | 1 |
| Sly-miR9469-5p | CCACAUAAGAAGACCGAAUUC | 0 | 1 |
| Sly-miR9470-3p | UGAAAUCCAUGAGCCUAAACU | 432 | 615 |
| Sly-miR9470-5p | UUUGGCUCAUGGAUUUUAGC | 4 | 4 |
| Sly-miR9471a-3p | CAGGUGCUCACUCAGCUAAUA | 12332 | 6171 |
| Sly-miR9471a-5p | UUGGCUGAGUGAGCAUCACGG | 44 | 24 |
| Sly-miR9471b-3p | UUGGCUGAGUGAGCAUCACUG | 10625 | 7475 |
| Sly-miR9471b-5p | GAGGUGCUCACUCAGCUAAUA | 7 | 6 |
| Sly-miR9472-3p | UUUCAGUAGACGUUGUGAAUA | 3 | 1 |
| Sly-miR9472-5p | UUCACAAUCUCUGCUGAAAAA | 22 | 2609 |
| Sly-miR9473-5p | UGGCUGUAAAUCUAAACUCGU | 13 | 61 |
| Sly-miR9474-3p | UUUUGUUCGCAGAUACUACAGU | 83 | 20 |
| Sly-miR9474-5p | UGUAGAAGUCAUGAAUAAAAUG | 533 | 83 |
| Sly-miR9475-3p | AACGAUCUCUACAUUGUAGGC | 268 | 609 |
| Sly-miR9475-5p | CUACAAUGUAGAGAUCGUUUU | 269 | 694 |
| Sly-miR9476-3p | UCUAGUCCUGCAUCUUUUUUU | 63 | 60 |
| Sly-miR9476-5p | AAAAAGAUGCAGGACUAGACC | 33 | 22 |
| Sly-miR9477-3p | UAUCCGUUGUUCCCUUUUCCUACC | 1 | 11 |
| Sly-miR9477-5p | UUGGGAAAGGGAACAACUGAUAGU | 0 | 4 |
| Sly-miR9478-3p | GCUUAAAUAUGUAGAUCGAACU | 2 | 7 |
| Sly-miR9478-5p | UUCGAUGACAUAUUUGAGCCU | 2 | 0 |
| Sly-miR9479-3p | UCCAGUCCUCUACCCUUCUCCA | 11 | 8 |
| Sly-miR9479-5p | GAGAAUGGUAGAGGGUCGGACC | 1 | 2 |
