# Supplementary material for: miRNA expression profiling and zeatin dynamic changes in a new model system of in vivo indirect regeneration of tomato
Source: PLoS One. 2020 Dec 17;15(12):e0237690. doi: 10.1371/journal.pone.0237690 (PMC7745965; doi:10.1371/journal.pone.0237690)
Supplement: S6 Table — (DOCX) [file pone.0237690.s008.docx]

**Table S6 | Novel miRNAs of two small RNA libraries derived from stem and callus.**

| **MiRNA** | **Sequence** | **Number of reads** | |
| --- | --- | --- | --- |
|  |  | **Stem** | **Callus** |
| Novel 1 | UAUUCCAUGAGACUGUUUUUGG | 14269 | 9669 |
| Novel 100 | AUGAAAAUCGUCUUGCCCCUCUUC | 9 | 11 |
| Novel 101 | CGCCAAAGGAGAGCUGCCCUG | 13 | 0 |
| Novel 105 | UCUUUGCAGAUCUGAGCCU | 1 | 2 |
| Novel 106 | UCUCUUUGCAGAUCUGAGCCUC | 2 | 4 |
| Novel 107 | AAGUCUGUCUCUGAGAUUUCGAGC | 30 | 0 |
| Novel 108 | AAGUUUGUCUCUGAGAUUUCGGGC | 262 | 33 |
| Novel 109 | AAGUGUGUCUCUGAGAUUUCGAGC | 17 | 25 |
| Novel 110 | AUAACUGUGUCUCUGAGAUUUCGG | 202 | 69 |
| Novel 111 | AUGAGUGUGUCUCUGAGAUUUCGG | 144 | 100 |
| Novel 112 | AAGAGUGUCUCUGAGAUUUCGUGC | 43 | 79 |
| Novel 113 | AAGUUUGUCUCUGAGAUUUCGAGU | 154 | 37 |
| Novel 114 | AAUUUCGGACAUAGGUUGAGGGGG | 112 | 41 |
| Novel 116 | AUUAGUGUGUCUCUGAGAUUUCGG | 15 | 85 |
| Novel 117 | AAUAUAGGUUAAGGACGUACUUGU | 20 | 6 |
| Novel 118 | AUGCACAAGUACCUCGUAGACUAU | 28 | 61 |
| Novel 119 | AUCAGUGUGUCUCUGAGAUUUCGG | 159 | 0 |
| Novel 120 | AUACAUCGGUUACUUGAUAGACGU | 51 | 86 |
| Novel 121 | AGACUAUAAUCGAAAUCUCAGAGA | 10 | 10 |
| Novel 125 | AAAUUUCGGACAUAGGUUGAGGGG | 71 | 26 |
| Novel 127 | AAGGCGUGUCUCUGAAAUUUCAGU | 78 | 86 |
| Novel 128 | AUUUCGAUCAAAGUUAAAAUAUUU | 6 | 14 |
| Novel 129 | AUUUCCGAGACACACUUAUACUGU | 10 | 14 |
| Novel 13 | UCGGACCAGGCUUCAUUCCCC | 524 | 60 |
| Novel 130 | ACUGACGUCAAGUUAAGGAUCAUG | 12 | 6 |
| Novel 131 | UAUGUCCUUUAACUUUGAGUGUGC | 56 | 17 |
| Novel 133 | UUUCAUUUCCGAACUAUUGACAGU | 39 | 40 |
| Novel 134 | UGGUUGUGUCGAAAUUGUAAGAGU | 8 | 9 |
| Novel 136 | AUCAUAGUCUAGGAGGUACUUGUG | 9 | 7 |
| Novel 137 | UUAUUUUAGUUAAGAUGUGUCUCU | 8 | 9 |
| Novel 139 | UUGGACUGAAGGGAGCUCCUA | 163 | 204 |
| Novel 140 | ACAAGUGUGUCUCUGAAAUUUCGG | 14 | 16 |
| Novel 141 | AUUGAAGGACAUAAAUGUGAAAUG | 11 | 3 |
| Novel 142 | UACGUGUGUCUCUGAGAUUUCGG | 39 | 4 |
| Novel 144 | AUAUAACUCCGAACUAUCGUAAAU | 13 | 10 |
| Novel 145 | AAGAUGUGUCUCUGAGAUUUCGAU | 336 | 499 |
| Novel 148 | GAAGGACAUAAAUAUGAAAUGAGG | 15 | 11 |
| Novel 149 | AGAGGACCUUAGUAUAGUAUAAGU | 28 | 38 |
| Novel 150 | AGACGUGUCUCUAAAAUUUCAAUC | 22 | 18 |
| Novel 152 | AUUGGACUUGUUCCUUUGACAUGU | 59 | 142 |
| Novel 153 | AAGAUGUGUCUCUGAAAUUUCGGU | 77 | 97 |
| Novel 154 | AUCCAAUGAAGUUGAGUGACGUGU | 23 | 32 |
| Novel 156 | AAAGGACAUAAAUGUGAAAUGAGG | 2 | 0 |
| Novel 159 | UUGGUGCUCAUGACGUCUAAAAGU | 16 | 22 |
| Novel 160 | AUUUCGGCUAAAGUUAAAGUAUUU | 9 | 19 |
| Novel 161 | UUUUAACUUGAAAAUGUAGAGAUU | 22 | 18 |
| Novel 162 | AAAGUGUGUCUCUGAAAUUUCAGU | 7 | 26 |
| Novel 164 | UUAUCUGGAGUUACAAGUUGA | 6 | 5 |
| Novel 166 | AAGUUUGUCUCUGAGAUUUCGAUC | 130 | 29 |
| Novel 167 | UUGGAUCCAAGUUGGACGAGGG | 11 | 50 |
| Novel 22 | UUCCACAGCUUUCUUGAACUU | 3 | 0 |
| Novel 31 | ACUCAAAUCCGAGAUCUCUGGUUA | 737 | 544 |
| Novel 32 | UGUGUUCUCAGGUUACCCCUG | 653 | 84 |
| Novel 33 | CUCUUCCGUGAACUUCAUCAAAAU | 283 | 908 |
| Novel 35 | UUGAAUGUUGGGGAACGAACGUCU | 420 | 556 |
| Novel 38 | GCUCUCUAUGCUUCUGUCAUC | 141 | 239 |
| Novel 40 | GUUUGUUGCUCCGAUUCGU | 41 | 43 |
| Novel 42 | UCUGUGUUUGGGAAGUAUGAC | 255 | 178 |
| Novel 43 | UGCACUGCCUCUUCCCUGGCU | 457 | 37 |
| Novel 44 | AACUGUUUCUACAUUCUCAAUACU | 82 | 532 |
| Novel 46 | UUGACAGAAGAUAGAGAGCAC | 1 | 4 |
| Novel 51 | AGAGGUCUUUCGAGUGAGAGUG | 22 | 212 |
| Novel 54 | GGUCAUGCUCGGACAGCCUCACU | 1 | 374 |
| Novel 55 | AAGUGUGUCUCUGAAAUUUCGAGC | 11 | 11 |
| Novel 56 | AAAUAUGUCUCUGAGAUUUCGAGC | 16 | 1 |
| Novel 58 | AUUUCGGACAUAAGUUAAGGAGGU | 66 | 18 |
| Novel 59 | UGUCGCAGAUGACUUUCGCCC | 1 | 2 |
| Novel 63 | AUAUAAACGUGACUCUUAAGUUGG | 20 | 15 |
| Novel 64 | AUCGGACCUUAGUAUAGUAUAAGU | 1 | 0 |
| Novel 66 | ACACACUCUGCAUUCAAUUAAAUU | 60 | 131 |
| Novel 67 | GUUUAAUAGAAUUAUGACACGUGU | 51 | 86 |
| Novel 70 | UAAGUUAUGGACUUAGUAUUG | 129 | 42 |
| Novel 75 | GGAAUCUUGAUGAUGCUGCAG | 110 | 34 |
| Novel 79 | UUUUGGUAUGUGAAGGUGAAUUGU | 96 | 5 |
| Novel 80 | UAUUUCUGCAGCUUUGGAAUU | 39 | 42 |
| Novel 83 | UAAAUGUUUUCCGAGUAUCUU | 19 | 29 |
| Novel 84 | AGAAUCUUGAUGAUGCUGCAU | 1 | 0 |
| Novel 85 | UGGUGAUUGUGGUUCGAAAAU | 73 | 1 |
| Novel 88 | AAUUUUCGAUCAGACUUAAACGGU | 32 | 30 |
| Novel 89 | AUAAGUGUGACUCUGAAAUUUCGG | 11 | 6 |
| Novel 9 | CUAUGAGAUAAGUUCAACGUG | 3958 | 3210 |
| Novel 93 | UGAAUCUUGAUGAUGCUGCAG | 42 | 11 |
