# Supplementary material for: miRNA expression profiling and zeatin dynamic changes in a new model system of in vivo indirect regeneration of tomato
Source: PLoS One. 2020 Dec 17;15(12):e0237690. doi: 10.1371/journal.pone.0237690 (PMC7745965; doi:10.1371/journal.pone.0237690)
Supplement: S7 Table — (DOCX) [file pone.0237690.s009.docx]

**Table S7 | Differentially expressed known and novel miRNAs during callus formation of tomato in vivo regeneration.**

| **MiRNA** | **Stem** | **Callus** | **log_2_ (callus/stem)** | **P-value** |
| --- | --- | --- | --- | --- |
| Sly-miR156d-3p | 22.42126351 | 69.9857987 | 1.6422 | 4.02E-09 |
| Sly-miR156d-5p | 711.8751163 | 1641.485097 | 1.2053 | 4.40E-124 |
| Sly-miR156e-3p | 50.44784289 | 6.362345336 | -2.9872 | 1.45E-08 |
| Sly-miR156e-5p | 532.5050083 | 260.8561588 | -1.0295 | 3.16E-13 |
| Sly-miR160a | 397.9774272 | 95.43518005 | -2.0601 | 5.82E-35 |
| Sly-miR164a-3p | 33.63189526 | 133.6092521 | 1.9901 | 1.40E-19 |
| Sly-miR164a-5p | 857.6133291 | 2182.28445 | 1.3474 | 6.04E-188 |
| Sly-miR164b-3p | 5.605315877 | 95.43518005 | 4.0897 | 9.00E-25 |
| Sly-miR166c-3p | 27841.60396 | 11051.39385 | -1.333 | 0 |
| Sly-miR166c-5p | 2623.28783 | 763.4814404 | -1.7807 | 1.08E-173 |
| Sly-miR167a | 61.65847464 | 1234.294995 | 4.3232 | 3.71E-306 |
| Sly-miR168a-5p | 3649.060636 | 1705.10855 | -1.0977 | 8.36E-94 |
| Sly-miR169a | 0 | 6.362345336 | 3.6696 | 0.0099328 |
| Sly-miR169e-3p | 801.5601704 | 127.2469067 | -2.6552 | 4.66E-98 |
| Sly-miR171a | 868.8239609 | 82.71048937 | -3.3929 | 5.55E-140 |
| Sly-miR171b | 134.527581 | 44.53641736 | -1.5948 | 8.25E-09 |
| Sly-miR171d | 117.7116334 | 19.08703601 | -2.6246 | 1.33E-15 |
| Sly-miR171e | 852.0080133 | 381.7407202 | -1.1583 | 6.57E-26 |
| Sly-miR319a | 5381.103242 | 2112.298652 | -1.3491 | 4.63E-212 |
| Sly-miR319b | 165205.4748 | 77251.59708 | -1.0966 | 0 |
| Sly-miR319c-3p | 84870.08769 | 35788.19252 | -1.2458 | 0 |
| Sly-miR319c-5p | 72.8691064 | 31.81172668 | -1.1957 | 0.0014463 |
| Sly-miR390a-3p | 84.07973815 | 184.5080148 | 1.1339 | 2.07E-14 |
| Sly-miR390b-3p | 39.23721114 | 82.71048937 | 1.0758 | 7.19E-07 |
| Sly-miR394-3p | 56.05315877 | 19.08703601 | -1.5542 | 0.00028095 |
| Sly-miR394-5p | 5549.262718 | 1641.485097 | -1.7573 | 0 |
| Sly-miR396a-3p | 4831.782286 | 279.9431948 | -4.1093 | 0 |
| Sly-miR396a-5p | 10437.09816 | 2990.302308 | -1.8034 | 0 |
| Sly-miR397 | 2320.600773 | 324.4796122 | -2.8383 | 1.52E-304 |
| Sly-miR4376 | 5095.232132 | 18730.74467 | 1.8782 | 0 |
| Sly-miR477-3p | 1563.88313 | 661.683915 | -1.2409 | 3.22E-53 |
| Sly-miR482d-5p | 33.63189526 | 69.9857987 | 1.0572 | 6.48E-06 |
| Sly-miR482e-5p | 1911.412714 | 4504.540498 | 1.2367 | 0 |
| Sly-miR5304 | 28.02657938 | 63.62345336 | 1.1828 | 4.06E-06 |
| Sly-miR6026 | 1922.623346 | 381.7407202 | -2.3324 | 5.52E-195 |
| Sly-miR6027-5p | 4119.907169 | 10065.23032 | 1.2887 | 0 |
| Sly-miR9469-3p | 0 | 6.362345336 | 3.6696 | 0.0099328 |
| Sly-miR9469-5p | 0 | 6.362345336 | 3.6696 | 0.0099328 |
| Sly-miR9472-5p | 123.3169493 | 16599.35898 | 7.0726 | 0 |
| Sly-miR9473-5p | 72.8691064 | 388.1030655 | 2.4131 | 4.67E-65 |
| Sly-miR9474-3p | 465.2412178 | 127.2469067 | -1.8703 | 6.26E-35 |
| Sly-miR9474-5p | 2987.633362 | 528.0746629 | -2.5002 | 0 |
| Sly-miR9475-3p | 1502.224655 | 3874.66831 | 1.367 | 0 |
| Sly-miR9475-5p | 1507.829971 | 4415.467663 | 1.5501 | 0 |
| Sly-miR9477-3p | 5.605315877 | 69.9857987 | 3.6422 | 1.44E-17 |
| Sly-miR9477-5p | 0 | 25.44938135 | 5.6696 | 3.77E-08 |
| Sly-miR9478-3p | 11.21063175 | 44.53641736 | 1.9901 | 1.73E-07 |
| Sly-miR9478-5p | 11.21063175 | 0 | -4.4868 | 0.0014063 |
| Sly-miR9479-5p | 5.605315877 | 12.72469067 | 1.1828 | 0.039323 |
| novel 54 | 5.605315877 | 2379.517156 | 8.7297 | 0 |
| novel 44 | 459.6359019 | 3384.767719 | 2.8805 | 0 |
| novel 33 | 1586.304393 | 5777.009565 | 1.8647 | 0 |
| novel 43 | 2561.629356 | 235.4067774 | -3.4438 | 0 |
| novel 13 | 2937.185519 | 381.7407202 | -2.9438 | 0 |
| novel 32 | 3660.271267 | 534.4370083 | -2.7759 | 0 |
| novel 51 | 123.3169493 | 1348.817211 | 3.4513 | 6.57E-297 |
| novel 108 | 1468.59276 | 209.9573961 | -2.8063 | 8.28E-191 |
| novel 119 | 891.2452244 | 0 | -10.8 | 5.27E-125 |
| novel 79 | 538.1103242 | 31.81172668 | -4.0803 | 3.96E-102 |
| novel 116 | 84.07973815 | 540.7993536 | 2.6853 | 3.96E-99 |
| novel 85 | 409.188059 | 6.362345336 | -6.0071 | 2.27E-90 |
| novel 152 | 330.7136367 | 903.4530378 | 1.4499 | 5.61E-86 |
| novel 66 | 336.3189526 | 833.4672391 | 1.3093 | 1.69E-70 |
| novel 113 | 863.218645 | 235.4067774 | -1.8746 | 1.51E-63 |
| novel 166 | 728.691064 | 184.5080148 | -1.9816 | 6.56E-59 |
| novel 167 | 61.65847464 | 318.1172668 | 2.3672 | 1.08E-52 |
| novel 110 | 1132.273807 | 439.0018282 | -1.3669 | 3.55E-47 |
| novel 112 | 241.0285827 | 502.6252816 | 1.0603 | 9.41E-34 |
| novel 70 | 723.0857481 | 267.2185041 | -1.4361 | 1.03E-33 |
| novel 107 | 168.1594763 | 0 | -8.3937 | 1.12E-33 |
| novel 118 | 156.9488445 | 388.1030655 | 1.3061 | 1.14E-33 |
| novel 142 | 218.6073192 | 25.44938135 | -3.1026 | 1.86E-33 |
| novel 75 | 616.5847464 | 216.3197414 | -1.5111 | 8.45E-32 |
| novel 162 | 39.23721114 | 165.4209787 | 2.0758 | 6.88E-25 |
| novel 58 | 369.9508479 | 114.5222161 | -1.6917 | 6.67E-24 |
| novel 114 | 627.7953782 | 260.8561588 | -1.267 | 2.77E-23 |
| novel 56 | 89.68505403 | 6.362345336 | -3.8172 | 1.39E-17 |
| novel 131 | 313.8976891 | 108.1598707 | -1.5371 | 1.77E-17 |
| novel 101 | 72.8691064 | 0 | -7.1872 | 6.41E-17 |
| novel 93 | 235.4232668 | 69.9857987 | -1.7501 | 1.17E-16 |
| novel 125 | 397.9774272 | 165.4209787 | -1.2665 | 2.55E-15 |
| novel 160 | 50.44784289 | 120.8845614 | 1.2608 | 3.97E-11 |
| novel 128 | 33.63189526 | 89.07283471 | 1.4052 | 1.41E-09 |
| novel 117 | 112.1063175 | 38.17407202 | -1.5542 | 2.79E-07 |
| novel 46 | 5.605315877 | 25.44938135 | 2.1828 | 3.35E-05 |
| novel 141 | 61.65847464 | 19.08703601 | -1.6917 | 3.87E-05 |
| novel 22 | 16.81594763 | 0 | -5.0718 | 5.74E-05 |
| novel 156 | 11.21063175 | 0 | -4.4868 | 0.0014063 |
| novel 106 | 11.21063175 | 25.44938135 | 1.1828 | 0.0035635 |
| novel 105 | 5.605315877 | 12.72469067 | 1.1828 | 0.039323 |
| novel 59 | 5.605315877 | 12.72469067 | 1.1828 | 0.039323 |
| novel 64 | 5.605315877 | 0 | -3.4868 | 0.040382 |
| novel 84 | 5.605315877 | 0 | -3.4868 | 0.040382 |
