# Supplementary material for: miRNA expression profiling and zeatin dynamic changes in a new model system of in vivo indirect regeneration of tomato
Source: PLoS One. 2020 Dec 17;15(12):e0237690. doi: 10.1371/journal.pone.0237690 (PMC7745965; doi:10.1371/journal.pone.0237690)
Supplement: S8 Table — (DOCX) [file pone.0237690.s010.docx]

**Table S8 | Target genes for differentially expressed known and novel miRNAs.**

| **MiRNA** | **Target gene ID** |
| --- | --- |
| Sly-miR156d-5p | Solyc01g005600.2 |
| Sly-miR156d-5p | Solyc01g008850.2 |
| Sly-miR156d-5p | Solyc01g010040.2 |
| Sly-miR156d-5p | Solyc01g010050.2 |
| Sly-miR156d-5p | Solyc01g011100.2 |
| Sly-miR156d-5p | Solyc01g068480.2 |
| Sly-miR156d-5p | Solyc01g073640.2 |
| Sly-miR156d-5p | Solyc01g094660.2 |
| Sly-miR156d-5p | Solyc01g095890.2 |
| Sly-miR156d-5p | Solyc01g096580.2 |
| Sly-miR156d-5p | Solyc01g096590.2 |
| Sly-miR156d-5p | Solyc01g098940.2 |
| Sly-miR156d-5p | Solyc01g099490.1 |
| Sly-miR156d-5p | Solyc01g101070.2 |
| Sly-miR156d-5p | Solyc01g105270.2 |
| Sly-miR156d-5p | Solyc01g111710.2 |
| Sly-miR156d-5p | Solyc02g032830.1 |
| Sly-miR156d-5p | Solyc02g067380.2 |
| Sly-miR156d-5p | Solyc02g071220.2 |
| Sly-miR156d-5p | Solyc02g077920.2 |
| Sly-miR156d-5p | Solyc02g084610.1 |
| Sly-miR156d-5p | Solyc02g084760.2 |
| Sly-miR156d-5p | Solyc03g005060.2 |
| Sly-miR156d-5p | Solyc03g005710.1 |
| Sly-miR156d-5p | Solyc03g097670.2 |
| Sly-miR156d-5p | Solyc03g114850.2 |
| Sly-miR156d-5p | Solyc03g117540.2 |
| Sly-miR156d-5p | Solyc04g009020.2 |
| Sly-miR156d-5p | Solyc04g010220.2 |
| Sly-miR156d-5p | Solyc04g045560.2 |
| Sly-miR156d-5p | Solyc04g077500.2 |
| Sly-miR156d-5p | Solyc05g012040.2 |
| Sly-miR156d-5p | Solyc05g015510.2 |
| Sly-miR156d-5p | Solyc05g015840.2 |
| Sly-miR156d-5p | Solyc05g052460.2 |
| Sly-miR156d-5p | Solyc06g051900.2 |
| Sly-miR156d-5p | Solyc06g072330.2 |
| Sly-miR156d-5p | Solyc07g005640.2 |
| Sly-miR156d-5p | Solyc07g045330.2 |
| Sly-miR156d-5p | Solyc07g054720.1 |
| Sly-miR156d-5p | Solyc07g062980.2 |
| Sly-miR156d-5p | Solyc08g069180.2 |
| Sly-miR156d-5p | Solyc08g076540.2 |
| Sly-miR156d-5p | Solyc09g055250.2 |
| Sly-miR156d-5p | Solyc09g059520.2 |
| Sly-miR156d-5p | Solyc09g063030.2 |
| Sly-miR156d-5p | Solyc09g064320.2 |
| Sly-miR156d-5p | Solyc09g074110.2 |
| Sly-miR156d-5p | Solyc09g091480.2 |
| Sly-miR156d-5p | Solyc10g009080.2 |
| Sly-miR156d-5p | Solyc10g039240.1 |
| Sly-miR156d-5p | Solyc10g047490.1 |
| Sly-miR156d-5p | Solyc10g078700.1 |
| Sly-miR156d-5p | Solyc10g080810.1 |
| Sly-miR156d-5p | Solyc11g011390.1 |
| Sly-miR156d-5p | Solyc11g012810.1 |
| Sly-miR156d-5p | Solyc11g065030.1 |
| Sly-miR156d-5p | Solyc12g010740.1 |
| Sly-miR156d-5p | Solyc12g019890.1 |
| Sly-miR156d-5p | Solyc12g038520.1 |
| Sly-miR156d-5p | Solyc12g082730.1 |
| Sly-miR156e-3p | Solyc01g050040.2 |
| Sly-miR156e-3p | Solyc01g110700.2 |
| Sly-miR156e-3p | Solyc01g111130.2 |
| Sly-miR156e-3p | Solyc01g111180.2 |
| Sly-miR156e-3p | Solyc11g013280.1 |
| Sly-miR156e-5p | Solyc01g005080.2 |
| Sly-miR156e-5p | Solyc01g005600.2 |
| Sly-miR156e-5p | Solyc01g011100.2 |
| Sly-miR156e-5p | Solyc01g079710.2 |
| Sly-miR156e-5p | Solyc01g080880.2 |
| Sly-miR156e-5p | Solyc01g086750.2 |
| Sly-miR156e-5p | Solyc01g095890.2 |
| Sly-miR156e-5p | Solyc01g105120.2 |
| Sly-miR156e-5p | Solyc01g105270.2 |
| Sly-miR156e-5p | Solyc02g032830.1 |
| Sly-miR156e-5p | Solyc02g067380.2 |
| Sly-miR156e-5p | Solyc02g069370.2 |
| Sly-miR156e-5p | Solyc02g070290.2 |
| Sly-miR156e-5p | Solyc02g079040.2 |
| Sly-miR156e-5p | Solyc02g081930.2 |
| Sly-miR156e-5p | Solyc02g091530.2 |
| Sly-miR156e-5p | Solyc03g005060.2 |
| Sly-miR156e-5p | Solyc03g005710.1 |
| Sly-miR156e-5p | Solyc03g007600.2 |
| Sly-miR156e-5p | Solyc03g097670.2 |
| Sly-miR156e-5p | Solyc03g114850.2 |
| Sly-miR156e-5p | Solyc03g116660.2 |
| Sly-miR156e-5p | Solyc03g116820.2 |
| Sly-miR156e-5p | Solyc03g121670.2 |
| Sly-miR156e-5p | Solyc03g121700.2 |
| Sly-miR156e-5p | Solyc04g009020.2 |
| Sly-miR156e-5p | Solyc04g045560.2 |
| Sly-miR156e-5p | Solyc04g045580.2 |
| Sly-miR156e-5p | Solyc04g074130.2 |
| Sly-miR156e-5p | Solyc05g005330.2 |
| Sly-miR156e-5p | Solyc05g012040.2 |
| Sly-miR156e-5p | Solyc05g013340.2 |
| Sly-miR156e-5p | Solyc05g015510.2 |
| Sly-miR156e-5p | Solyc05g015840.2 |
| Sly-miR156e-5p | Solyc05g052460.2 |
| Sly-miR156e-5p | Solyc06g035790.2 |
| Sly-miR156e-5p | Solyc06g060120.2 |
| Sly-miR156e-5p | Solyc06g075020.2 |
| Sly-miR156e-5p | Solyc07g005640.2 |
| Sly-miR156e-5p | Solyc07g045330.2 |
| Sly-miR156e-5p | Solyc07g054720.1 |
| Sly-miR156e-5p | Solyc07g061970.2 |
| Sly-miR156e-5p | Solyc07g062980.2 |
| Sly-miR156e-5p | Solyc08g061560.2 |
| Sly-miR156e-5p | Solyc08g069180.2 |
| Sly-miR156e-5p | Solyc08g078560.2 |
| Sly-miR156e-5p | Solyc08g081420.2 |
| Sly-miR156e-5p | Solyc08g083300.2 |
| Sly-miR156e-5p | Solyc09g059520.2 |
| Sly-miR156e-5p | Solyc09g074110.2 |
| Sly-miR156e-5p | Solyc10g005450.1 |
| Sly-miR156e-5p | Solyc10g009080.2 |
| Sly-miR156e-5p | Solyc10g078700.1 |
| Sly-miR156e-5p | Solyc10g080810.1 |
| Sly-miR156e-5p | Solyc11g005340.1 |
| Sly-miR156e-5p | Solyc11g012810.1 |
| Sly-miR156e-5p | Solyc11g013460.1 |
| Sly-miR156e-5p | Solyc12g010740.1 |
| Sly-miR156e-5p | Solyc12g038520.1 |
| Sly-miR160a | Solyc01g011350.2 |
| Sly-miR160a | Solyc03g117090.2 |
| Sly-miR160a | Solyc05g050770.2 |
| Sly-miR160a | Solyc06g075150.2 |
| Sly-miR160a | Solyc09g007810.2 |
| Sly-miR160a | Solyc09g075770.1 |
| Sly-miR160a | Solyc10g086130.1 |
| Sly-miR160a | Solyc11g013470.1 |
| Sly-miR160a | Solyc11g069500.1 |
| Sly-miR164a-3p | Solyc01g057900.2 |
| Sly-miR164a-3p | Solyc01g081140.1 |
| Sly-miR164a-3p | Solyc09g082560.2 |
| Sly-miR164a-5p | Solyc01g088690.2 |
| Sly-miR164a-5p | Solyc01g090590.2 |
| Sly-miR164a-5p | Solyc06g051810.2 |
| Sly-miR164a-5p | Solyc06g069710.2 |
| Sly-miR164a-5p | Solyc07g062840.2 |
| Sly-miR164a-5p | Solyc07g066330.2 |
| Sly-miR164a-5p | Solyc09g005810.2 |
| Sly-miR164a-5p | Solyc09g008600.2 |
| Sly-miR164a-5p | Solyc09g075120.2 |
| Sly-miR164a-5p | Solyc11g022540.1 |
| Sly-miR164a-5p | Solyc11g065620.1 |
| Sly-miR164a-5p | Solyc11g066150.1 |
| Sly-miR164b-3p | Solyc00g154980.1 |
| Sly-miR164b-3p | Solyc03g111790.2 |
| Sly-miR164b-3p | Solyc07g051940.2 |
| Sly-miR166c-3p | Solyc05g008940.2 |
| Sly-miR166c-3p | Solyc09g018370.2 |
| Sly-miR166c-5p | Solyc01g112300.2 |
| Sly-miR166c-5p | Solyc05g005890.2 |
| Sly-miR166c-5p | Solyc05g005900.2 |
| Sly-miR167a | Solyc03g117700.1 |
| Sly-miR167a | Solyc05g055770.2 |
| Sly-miR167a | Solyc06g035710.1 |
| Sly-miR167a | Solyc06g036780.2 |
| Sly-miR167a | Solyc08g065360.2 |
| Sly-miR167a | Solyc11g073300.1 |
| Sly-miR167a | Solyc12g044880.1 |
| Sly-miR168a-5p | Solyc00g030110.1 |
| Sly-miR168a-5p | Solyc03g098280.2 |
| Sly-miR168a-5p | Solyc05g053230.2 |
| Sly-miR168a-5p | Solyc06g072300.2 |
| Sly-miR168a-5p | Solyc08g005690.1 |
| Sly-miR169a | Solyc01g008490.2 |
| Sly-miR169a | Solyc01g087240.2 |
| Sly-miR169a | Solyc03g121940.2 |
| Sly-miR169a | Solyc06g068930.1 |
| Sly-miR169a | Solyc07g042160.2 |
| Sly-miR169a | Solyc08g007030.2 |
| Sly-miR169a | Solyc08g062210.2 |
| Sly-miR169a | Solyc08g075730.2 |
| Sly-miR169a | Solyc10g012030.2 |
| Sly-miR169a | Solyc10g078390.1 |
| Sly-miR169a | Solyc10g085720.1 |
| Sly-miR169e-3p | Solyc00g016470.1 |
| Sly-miR169e-3p | Solyc01g057570.2 |
| Sly-miR169e-3p | Solyc03g058470.1 |
| Sly-miR169e-3p | Solyc03g124000.1 |
| Sly-miR169e-3p | Solyc04g055180.2 |
| Sly-miR169e-3p | Solyc06g051520.2 |
| Sly-miR169e-3p | Solyc06g082560.1 |
| Sly-miR169e-3p | Solyc07g008450.2 |
| Sly-miR169e-3p | Solyc11g013030.1 |
| Sly-miR169e-3p | Solyc11g050840.1 |
| Sly-miR169e-3p | Solyc11g067110.1 |
| Sly-miR171a | Solyc01g090950.2 |
| Sly-miR171a | Solyc02g085600.1 |
| Sly-miR171a | Solyc08g078800.1 |
| Sly-miR171a | Solyc08g081890.2 |
| Sly-miR171b | Solyc01g090950.2 |
| Sly-miR171b | Solyc02g085600.1 |
| Sly-miR171b | Solyc03g113470.1 |
| Sly-miR171b | Solyc08g008310.2 |
| Sly-miR171b | Solyc08g078800.1 |
| Sly-miR171b | Solyc11g013150.1 |
| Sly-miR171d | Solyc01g090950.2 |
| Sly-miR171d | Solyc02g079100.2 |
| Sly-miR171d | Solyc08g078800.1 |
| Sly-miR171d | Solyc11g013150.1 |
| Sly-miR171e | Solyc01g090950.2 |
| Sly-miR171e | Solyc05g055610.2 |
| Sly-miR171e | Solyc06g075460.2 |
| Sly-miR171e | Solyc06g075480.2 |
| Sly-miR171e | Solyc06g075500.2 |
| Sly-miR171e | Solyc08g078800.1 |
| Sly-miR171e | Solyc11g013150.1 |
| Sly-miR319a | Solyc01g008770.2 |
| Sly-miR319a | Solyc01g009070.2 |
| Sly-miR319a | Solyc02g079010.2 |
| Sly-miR319a | Solyc04g056280.2 |
| Sly-miR319a | Solyc06g073640.2 |
| Sly-miR319a | Solyc09g009340.1 |
| Sly-miR319a | Solyc11g072060.1 |
| Sly-miR319a | Solyc12g096420.1 |
| Sly-miR319b | Solyc02g079010.2 |
| Sly-miR319b | Solyc04g056280.2 |
| Sly-miR319c-3p | Solyc01g009070.2 |
| Sly-miR319c-3p | Solyc04g050790.2 |
| Sly-miR319c-3p | Solyc04g056280.2 |
| Sly-miR319c-5p | Solyc01g109080.2 |
| Sly-miR319c-5p | Solyc02g069490.2 |
| Sly-miR319c-5p | Solyc03g095360.2 |
| Sly-miR319c-5p | Solyc06g051760.2 |
| Sly-miR319c-5p | Solyc08g080190.2 |
| Sly-miR319c-5p | Solyc12g099990.1 |
| Sly-miR390a-3p | Solyc00g052940.2 |
| Sly-miR390b-3p | Solyc03g116150.2 |
| Sly-miR394-3p | Solyc01g090440.2 |
| Sly-miR394-3p | Solyc04g082290.2 |
| Sly-miR394-3p | Solyc06g059760.2 |
| Sly-miR394-3p | Solyc06g076200.1 |
| Sly-miR394-3p | Solyc06g076210.1 |
| Sly-miR394-3p | Solyc09g074110.2 |
| Sly-miR394-3p | Solyc12g009020.1 |
| Sly-miR394-3p | Solyc12g077670.1 |
| Sly-miR394-3p | Solyc12g094510.1 |
| Sly-miR394-5p | Solyc01g044300.1 |
| Sly-miR394-5p | Solyc01g066060.2 |
| Sly-miR394-5p | Solyc05g015520.2 |
| Sly-miR394-5p | Solyc06g009220.2 |
| Sly-miR394-5p | Solyc08g042140.2 |
| Sly-miR394-5p | Solyc11g007270.1 |
| Sly-miR396a-3p | Solyc01g091150.2 |
| Sly-miR396a-3p | Solyc03g006650.1 |
| Sly-miR396a-3p | Solyc04g050470.2 |
| Sly-miR396a-3p | Solyc06g036440.1 |
| Sly-miR396a-3p | Solyc06g063080.1 |
| Sly-miR396a-3p | Solyc06g083620.2 |
| Sly-miR396a-3p | Solyc08g079810.2 |
| Sly-miR396a-3p | Solyc09g065010.2 |
| Sly-miR396a-3p | Solyc10g038190.1 |
| Sly-miR396a-3p | Solyc11g071840.1 |
| Sly-miR396a-3p | Solyc12g038400.1 |
| Sly-miR396a-3p | Solyc12g098630.1 |
| Sly-miR396a-5p | Solyc00g105750.1 |
| Sly-miR396a-5p | Solyc01g009010.2 |
| Sly-miR396a-5p | Solyc01g066730.2 |
| Sly-miR396a-5p | Solyc01g067450.1 |
| Sly-miR396a-5p | Solyc01g081050.2 |
| Sly-miR396a-5p | Solyc01g108340.2 |
| Sly-miR396a-5p | Solyc02g062740.2 |
| Sly-miR396a-5p | Solyc02g071140.2 |
| Sly-miR396a-5p | Solyc02g083190.1 |
| Sly-miR396a-5p | Solyc03g110900.2 |
| Sly-miR396a-5p | Solyc03g117960.2 |
| Sly-miR396a-5p | Solyc04g076390.2 |
| Sly-miR396a-5p | Solyc05g054840.2 |
| Sly-miR396a-5p | Solyc06g007320.2 |
| Sly-miR396a-5p | Solyc06g054340.1 |
| Sly-miR396a-5p | Solyc07g019640.1 |
| Sly-miR396a-5p | Solyc07g052390.2 |
| Sly-miR396a-5p | Solyc08g066140.2 |
| Sly-miR396a-5p | Solyc08g067250.2 |
| Sly-miR396a-5p | Solyc08g068020.1 |
| Sly-miR396a-5p | Solyc08g082010.2 |
| Sly-miR396a-5p | Solyc09g014840.1 |
| Sly-miR396a-5p | Solyc09g082510.2 |
| Sly-miR396a-5p | Solyc12g008590.1 |
| Sly-miR396a-5p | Solyc12g044440.1 |
| Sly-miR396a-5p | Solyc12g088700.1 |
| Sly-miR397 | Solyc01g104280.2 |
| Sly-miR397 | Solyc01g108920.2 |
| Sly-miR397 | Solyc02g049070.2 |
| Sly-miR397 | Solyc02g062650.2 |
| Sly-miR397 | Solyc02g078690.1 |
| Sly-miR397 | Solyc02g085110.2 |
| Sly-miR397 | Solyc02g085120.2 |
| Sly-miR397 | Solyc03g095220.2 |
| Sly-miR397 | Solyc05g012250.1 |
| Sly-miR397 | Solyc05g043360.1 |
| Sly-miR397 | Solyc06g048860.1 |
| Sly-miR397 | Solyc06g050530.2 |
| Sly-miR397 | Solyc06g074350.2 |
| Sly-miR397 | Solyc06g076330.2 |
| Sly-miR397 | Solyc06g076760.1 |
| Sly-miR397 | Solyc06g082240.2 |
| Sly-miR397 | Solyc07g049460.2 |
| Sly-miR397 | Solyc07g063920.2 |
| Sly-miR397 | Solyc09g009350.2 |
| Sly-miR397 | Solyc09g010990.2 |
| Sly-miR397 | Solyc09g011000.2 |
| Sly-miR397 | Solyc09g011050.2 |
| Sly-miR397 | Solyc09g011960.1 |
| Sly-miR397 | Solyc09g011970.1 |
| Sly-miR397 | Solyc09g014240.2 |
| Sly-miR397 | Solyc09g014820.2 |
| Sly-miR397 | Solyc10g076710.1 |
| Sly-miR397 | Solyc10g076830.1 |
| Sly-miR397 | Solyc10g082000.1 |
| Sly-miR397 | Solyc11g006650.1 |
| Sly-miR397 | Solyc11g012860.1 |
| Sly-miR4376 | Solyc02g085810.2 |
| Sly-miR4376 | Solyc07g008320.2 |
| Sly-miR4376 | Solyc07g009150.2 |
| Sly-miR477-3p | Solyc00g007260.2 |
| Sly-miR477-3p | Solyc01g096010.2 |
| Sly-miR477-3p | Solyc02g032330.2 |
| Sly-miR477-3p | Solyc10g075030.1 |
| Sly-miR482d-5p | Solyc03g115200.2 |
| Sly-miR482d-5p | Solyc04g054460.2 |
| Sly-miR482d-5p | Solyc05g012260.2 |
| Sly-miR482d-5p | Solyc06g076860.2 |
| Sly-miR482d-5p | Solyc07g008450.2 |
| Sly-miR482d-5p | Solyc07g015860.2 |
| Sly-miR482d-5p | Solyc09g065670.2 |
| Sly-miR482d-5p | Solyc10g077120.1 |
| Sly-miR482d-5p | Solyc11g005100.1 |
| Sly-miR482d-5p | Solyc11g017000.1 |
| Sly-miR482d-5p | Solyc11g069400.1 |
| Sly-miR482e-5p | Solyc01g087420.2 |
| Sly-miR482e-5p | Solyc02g032960.2 |
| Sly-miR482e-5p | Solyc02g094500.2 |
| Sly-miR482e-5p | Solyc03g118680.2 |
| Sly-miR482e-5p | Solyc04g008680.2 |
| Sly-miR482e-5p | Solyc04g008730.2 |
| Sly-miR482e-5p | Solyc04g040110.2 |
| Sly-miR482e-5p | Solyc04g050150.2 |
| Sly-miR482e-5p | Solyc04g056410.2 |
| Sly-miR482e-5p | Solyc05g043320.1 |
| Sly-miR482e-5p | Solyc06g084590.2 |
| Sly-miR482e-5p | Solyc07g063330.2 |
| Sly-miR482e-5p | Solyc07g063350.2 |
| Sly-miR482e-5p | Solyc08g075510.2 |
| Sly-miR482e-5p | Solyc09g007390.2 |
| Sly-miR482e-5p | Solyc09g065650.2 |
| Sly-miR5304 | Solyc07g022880.2 |
| Sly-miR6026 | Solyc01g006200.2 |
| Sly-miR6026 | Solyc02g050190.1 |
| Sly-miR6026 | Solyc02g070750.1 |
| Sly-miR6026 | Solyc05g012890.1 |
| Sly-miR6026 | Solyc05g012910.2 |
| Sly-miR6026 | Solyc09g018220.1 |
| Sly-miR6026 | Solyc09g092290.1 |
| Sly-miR6026 | Solyc10g051170.1 |
| Sly-miR6026 | Solyc12g017800.1 |
| Sly-miR6027-5p | Solyc04g077020.2 |
| Sly-miR6027-5p | Solyc07g008950.2 |
| Sly-miR6027-5p | Solyc07g047990.1 |
| Sly-miR6027-5p | Solyc08g067090.2 |
| Sly-miR6027-5p | Solyc10g049340.1 |
| Sly-miR9469-3p | Solyc01g091090.2 |
| Sly-miR9469-3p | Solyc01g091150.2 |
| Sly-miR9469-3p | Solyc01g111550.2 |
| Sly-miR9469-3p | Solyc02g084620.2 |
| Sly-miR9469-3p | Solyc03g005410.2 |
| Sly-miR9469-3p | Solyc03g006500.2 |
| Sly-miR9469-3p | Solyc03g044430.2 |
| Sly-miR9469-3p | Solyc03g110880.2 |
| Sly-miR9469-3p | Solyc04g077650.2 |
| Sly-miR9469-3p | Solyc06g053750.2 |
| Sly-miR9469-3p | Solyc06g072340.2 |
| Sly-miR9469-3p | Solyc07g040740.2 |
| Sly-miR9469-3p | Solyc08g029230.2 |
| Sly-miR9469-3p | Solyc08g061500.1 |
| Sly-miR9469-3p | Solyc09g074110.2 |
| Sly-miR9469-3p | Solyc12g098160.1 |
| Sly-miR9469-5p | Solyc01g006610.1 |
| Sly-miR9469-5p | Solyc03g112950.2 |
| Sly-miR9469-5p | Solyc03g118890.2 |
| Sly-miR9469-5p | Solyc04g045470.2 |
| Sly-miR9469-5p | Solyc04g051570.2 |
| Sly-miR9469-5p | Solyc06g065730.2 |
| Sly-miR9469-5p | Solyc06g066790.2 |
| Sly-miR9469-5p | Solyc11g044530.1 |
| Sly-miR9469-5p | Solyc12g027800.1 |
| Sly-miR9472-5p | Solyc01g107750.2 |
| Sly-miR9472-5p | Solyc02g085400.2 |
| Sly-miR9472-5p | Solyc03g097990.2 |
| Sly-miR9472-5p | Solyc03g119250.2 |
| Sly-miR9472-5p | Solyc06g060320.2 |
| Sly-miR9472-5p | Solyc11g070170.1 |
| Sly-miR9472-5p | Solyc12g014530.1 |
| Sly-miR9473-5p | Solyc01g108460.1 |
| Sly-miR9473-5p | Solyc03g026190.2 |
| Sly-miR9473-5p | Solyc04g015310.1 |
| Sly-miR9473-5p | Solyc07g008310.2 |
| Sly-miR9473-5p | Solyc08g041950.1 |
| Sly-miR9473-5p | Solyc08g042030.1 |
| Sly-miR9473-5p | Solyc10g054680.1 |
| Sly-miR9473-5p | Solyc12g035680.1 |
| Sly-miR9474-3p | Solyc01g107870.2 |
| Sly-miR9474-3p | Solyc03g032120.2 |
| Sly-miR9474-3p | Solyc11g045610.1 |
| Sly-miR9474-5p | Solyc01g056990.2 |
| Sly-miR9474-5p | Solyc01g080120.2 |
| Sly-miR9474-5p | Solyc01g108290.2 |
| Sly-miR9474-5p | Solyc03g121500.2 |
| Sly-miR9474-5p | Solyc05g009470.2 |
| Sly-miR9474-5p | Solyc05g016690.2 |
| Sly-miR9474-5p | Solyc06g036620.1 |
| Sly-miR9474-5p | Solyc06g050880.2 |
| Sly-miR9474-5p | Solyc07g042790.1 |
| Sly-miR9474-5p | Solyc08g006870.2 |
| Sly-miR9474-5p | Solyc08g077520.2 |
| Sly-miR9474-5p | Solyc10g049480.1 |
| Sly-miR9474-5p | Solyc12g019310.1 |
| Sly-miR9475-3p | Solyc00g231680.1 |
| Sly-miR9475-3p | Solyc03g006900.1 |
| Sly-miR9475-3p | Solyc08g016160.2 |
| Sly-miR9475-3p | Solyc09g064240.2 |
| Sly-miR9475-3p | Solyc09g092470.2 |
| Sly-miR9475-3p | Solyc10g086390.1 |
| Sly-miR9475-5p | Solyc05g008340.2 |
| Sly-miR9475-5p | Solyc08g078250.2 |
| Sly-miR9475-5p | Solyc09g064240.2 |
| Sly-miR9477-3p | Solyc01g079240.2 |
| Sly-miR9477-3p | Solyc03g006070.2 |
| Sly-miR9477-3p | Solyc03g032110.1 |
| Sly-miR9477-3p | Solyc05g047700.1 |
| Sly-miR9477-3p | Solyc05g053860.2 |
| Sly-miR9477-3p | Solyc06g034070.1 |
| Sly-miR9477-3p | Solyc06g071150.2 |
| Sly-miR9477-3p | Solyc07g006150.2 |
| Sly-miR9477-3p | Solyc07g006510.2 |
| Sly-miR9477-3p | Solyc07g049500.2 |
| Sly-miR9477-3p | Solyc09g008120.2 |
| Sly-miR9477-3p | Solyc09g090280.1 |
| Sly-miR9477-3p | Solyc10g006440.2 |
| Sly-miR9477-3p | Solyc10g009520.2 |
| Sly-miR9477-3p | Solyc11g011530.1 |
| Sly-miR9477-3p | Solyc11g045680.1 |
| Sly-miR9477-3p | Solyc11g069270.1 |
| Sly-miR9477-3p | Solyc12g006850.1 |
| Sly-miR9477-3p | Solyc12g009450.1 |
| Sly-miR9477-3p | Solyc12g010110.1 |
| Sly-miR9477-3p | Solyc12g049500.1 |
| Sly-miR9477-3p | Solyc12g056020.1 |
| Sly-miR9477-5p | Solyc01g006720.2 |
| Sly-miR9477-5p | Solyc01g007950.2 |
| Sly-miR9477-5p | Solyc01g056720.2 |
| Sly-miR9477-5p | Solyc04g009030.2 |
| Sly-miR9477-5p | Solyc06g005660.1 |
| Sly-miR9477-5p | Solyc06g034070.1 |
| Sly-miR9477-5p | Solyc06g071150.2 |
| Sly-miR9477-5p | Solyc07g006150.2 |
| Sly-miR9477-5p | Solyc07g061920.2 |
| Sly-miR9477-5p | Solyc08g006000.2 |
| Sly-miR9477-5p | Solyc08g077860.2 |
| Sly-miR9477-5p | Solyc08g081200.2 |
| Sly-miR9477-5p | Solyc09g083180.2 |
| Sly-miR9477-5p | Solyc09g097900.2 |
| Sly-miR9477-5p | Solyc10g005450.1 |
| Sly-miR9477-5p | Solyc10g078340.1 |
| Sly-miR9477-5p | Solyc10g078700.1 |
| Sly-miR9477-5p | Solyc11g010350.1 |
| Sly-miR9477-5p | Solyc11g010840.1 |
| Sly-miR9477-5p | Solyc11g011530.1 |
| Sly-miR9477-5p | Solyc11g067260.1 |
| Sly-miR9477-5p | Solyc11g069270.1 |
| Sly-miR9477-5p | Solyc11g072580.1 |
| Sly-miR9477-5p | Solyc12g087830.1 |
| Sly-miR9477-5p | Solyc12g088830.1 |
| Sly-miR9477-5p | Solyc12g095810.1 |
| Sly-miR9478-3p | Solyc01g006620.2 |
| Sly-miR9478-3p | Solyc01g007890.1 |
| Sly-miR9478-3p | Solyc01g010770.2 |
| Sly-miR9478-3p | Solyc01g058520.2 |
| Sly-miR9478-3p | Solyc01g068370.2 |
| Sly-miR9478-3p | Solyc01g080360.2 |
| Sly-miR9478-3p | Solyc02g005410.1 |
| Sly-miR9478-3p | Solyc02g031750.2 |
| Sly-miR9478-3p | Solyc02g061780.2 |
| Sly-miR9478-3p | Solyc02g093550.2 |
| Sly-miR9478-3p | Solyc03g005820.2 |
| Sly-miR9478-3p | Solyc03g062890.2 |
| Sly-miR9478-3p | Solyc04g007070.2 |
| Sly-miR9478-3p | Solyc04g007590.1 |
| Sly-miR9478-3p | Solyc04g007960.2 |
| Sly-miR9478-3p | Solyc04g024710.2 |
| Sly-miR9478-3p | Solyc04g072380.2 |
| Sly-miR9478-3p | Solyc04g076290.2 |
| Sly-miR9478-3p | Solyc05g052450.2 |
| Sly-miR9478-3p | Solyc05g053240.2 |
| Sly-miR9478-3p | Solyc06g010200.2 |
| Sly-miR9478-3p | Solyc06g071040.2 |
| Sly-miR9478-3p | Solyc06g084570.2 |
| Sly-miR9478-3p | Solyc07g008130.2 |
| Sly-miR9478-3p | Solyc07g008690.2 |
| Sly-miR9478-3p | Solyc07g014680.2 |
| Sly-miR9478-3p | Solyc07g017920.1 |
| Sly-miR9478-3p | Solyc08g014420.2 |
| Sly-miR9478-3p | Solyc10g005490.2 |
| Sly-miR9478-3p | Solyc10g006930.2 |
| Sly-miR9478-3p | Solyc10g018120.1 |
| Sly-miR9478-3p | Solyc10g018390.1 |
| Sly-miR9478-3p | Solyc10g080930.1 |
| Sly-miR9478-3p | Solyc10g085740.1 |
| Sly-miR9478-3p | Solyc11g044320.1 |
| Sly-miR9478-3p | Solyc11g045380.1 |
| Sly-miR9478-3p | Solyc11g071600.1 |
| Sly-miR9478-3p | Solyc12g006510.1 |
| Sly-miR9478-3p | Solyc12g017830.1 |
| Sly-miR9478-3p | Solyc12g019000.1 |
| Sly-miR9478-3p | Solyc12g019050.1 |
| Sly-miR9478-5p | Solyc04g025260.2 |
| Sly-miR9478-5p | Solyc07g062230.2 |
| Sly-miR9478-5p | Solyc10g055450.1 |
| Sly-miR9478-5p | Solyc12g042580.1 |
| Sly-miR9479-5p | Solyc01g008120.2 |
| Sly-miR9479-5p | Solyc01g086710.2 |
| Sly-miR9479-5p | Solyc01g095210.2 |
| Sly-miR9479-5p | Solyc01g095480.2 |
| Sly-miR9479-5p | Solyc01g096480.2 |
| Sly-miR9479-5p | Solyc01g096890.2 |
| Sly-miR9479-5p | Solyc01g100360.2 |
| Sly-miR9479-5p | Solyc01g107510.2 |
| Sly-miR9479-5p | Solyc01g109540.2 |
| Sly-miR9479-5p | Solyc02g069820.2 |
| Sly-miR9479-5p | Solyc02g079880.2 |
| Sly-miR9479-5p | Solyc03g007100.2 |
| Sly-miR9479-5p | Solyc03g095580.1 |
| Sly-miR9479-5p | Solyc03g117010.2 |
| Sly-miR9479-5p | Solyc03g118530.2 |
| Sly-miR9479-5p | Solyc03g119650.2 |
| Sly-miR9479-5p | Solyc04g005190.2 |
| Sly-miR9479-5p | Solyc04g015320.1 |
| Sly-miR9479-5p | Solyc04g050540.2 |
| Sly-miR9479-5p | Solyc04g082640.2 |
| Sly-miR9479-5p | Solyc05g006640.2 |
| Sly-miR9479-5p | Solyc05g048860.2 |
| Sly-miR9479-5p | Solyc06g007380.1 |
| Sly-miR9479-5p | Solyc06g070980.2 |
| Sly-miR9479-5p | Solyc07g042980.2 |
| Sly-miR9479-5p | Solyc07g055290.2 |
| Sly-miR9479-5p | Solyc07g066290.2 |
| Sly-miR9479-5p | Solyc09g055760.2 |
| Sly-miR9479-5p | Solyc09g064840.2 |
| Sly-miR9479-5p | Solyc10g005920.2 |
| Sly-miR9479-5p | Solyc10g007290.2 |
| Sly-miR9479-5p | Solyc11g006620.1 |
| Sly-miR9479-5p | Solyc11g013280.1 |
| Sly-miR9479-5p | Solyc12g008710.1 |
| Sly-miR9479-5p | Solyc12g088010.1 |
| Sly-miR9479-5p | Solyc12g088960.1 |
| Novel 46 | Solyc04g045560.2 |
| Novel 46 | Solyc05g012040.2 |
| Novel 46 | Solyc05g015510.2 |
| Novel 46 | Solyc05g015840.2 |
| Novel 46 | Solyc10g078700.1 |
| Novel 46 | Solyc12g038520.1 |
